# Development of Fluorescent AF64394 Analogues Enables Real-Time Binding Studies for the Orphan Class A GPCR GPR3

**DOI:** 10.1021/acs.jmedchem.3c01707

**Published:** 2023-10-31

**Authors:** Merlin Bresinsky, Aida Shahraki, Peter Kolb, Steffen Pockes, Hannes Schihada

**Affiliations:** † Institute of Pharmacy, University of Regensburg, Universitätsstraße 31, 93053 Regensburg, Germany; ‡ Department of Pharmaceutical Chemistry, University of Marburg, Marbacher Weg 8, 35032 Marburg, Germany; § Department of Medicinal Chemistry, Institute for Therapeutics Discovery and Development, University of Minnesota, Minneapolis, Minnesota 55414, United States

## Abstract

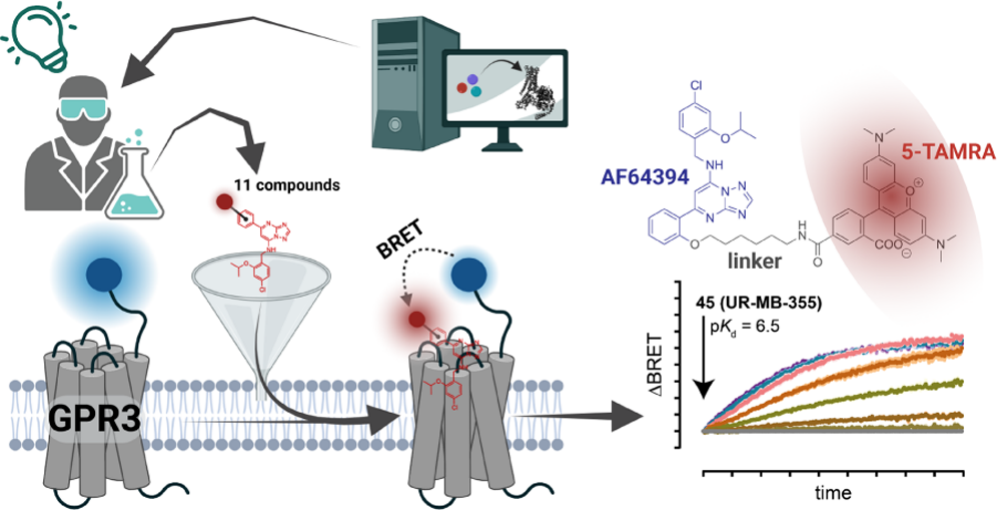

The orphan G protein-coupled receptor (oGPCR) GPR3 represents a potential drug target for the treatment of Alzheimer’s disease and metabolic disorders. However, the limited toolbox of pharmacological assays hampers the development of advanced ligands. Here, we developed a signaling pathway-independent readout of compound–GPR3 interaction. Starting from computational binding pose predictions of the most potent GPR3 ligand, we designed a series of fluorescent AF64394 analogues and assessed their suitability for BRET-based binding studies. The most potent ligand, **45** (UR-MB-355), bound to GPR3 and closely related receptors, GPR6 and GPR12, with similar submicromolar affinities. Furthermore, we found that **45** engages GPR3 in a distinct mode compared to AF64394, and coincubation studies with the GPR3 agonist diphenyleneiodonium chloride revealed allosteric modulation of **45** binding. These insights provide new cues for the pharmacological manipulation of GPR3 activity. This novel binding assay will foster the development of future drugs acting through these pharmacologically attractive oGPCRs.

## Introduction

The orphan class A G protein-coupled receptor (GPCR) GPR3 is a transmembrane (TM) protein that was first described in 1994 and is encoded by the *GPR3* gene.^1^ GPR3 is predominantly expressed in the brain, but it is also detected in the testis, ovary, eye, and other peripheral organs.^2,3^ Expression of GPR3 results in constitutive stimulation of adenylate cyclase (AC) and, thus, elevated levels of the second messenger cAMP.^2^ GPR3, together with GPR6 and GPR12, is part of a cluster of class A orphan GPCRs that are phylogenetically related to lipid receptors such as CB_1/2_, LPAR_1–5_, S1PR_1–5_, and melanocortin receptors.^4−6^ In addition to constitutive activation of heterotrimeric G_s_ by all three orphan GPCRs, GPR3 and GPR12 have also been suggested to activate inhibitory G proteins of the G_i/o_ family in a ligand-independent manner.^4,7,8^

Physiologically, GPR3 is involved in a variety of central and peripheral processes. For instance, GPR3 maintains meiotic arrest in oocytes by keeping increased levels of cAMP, and agonists of this receptor could therefore provide clues to treat reproductive disorders.^9,10^ Likewise, advanced GPR3 agonists could represent promising drugs against metabolic disorders because upregulated expression of the receptor in adipocytes drives energy expenditure and amplifies the physiological response to caloric excess.^11^ In the central nervous system, GPR3/6/12―GPR3 in particular―is associated with neurite outgrowth, neuronal cell survival, and axonal regeneration.^12−15^ Furthermore, GPR3 mediates the formation of amyloid-β peptides in neurons by interacting with β-arrestin2 and stimulating γ-secretase activity, hinting at the therapeutic potential for tackling Alzheimer’s disease with GPR3 antagonists or inverse agonists.^16−18^

**Figure 1:**
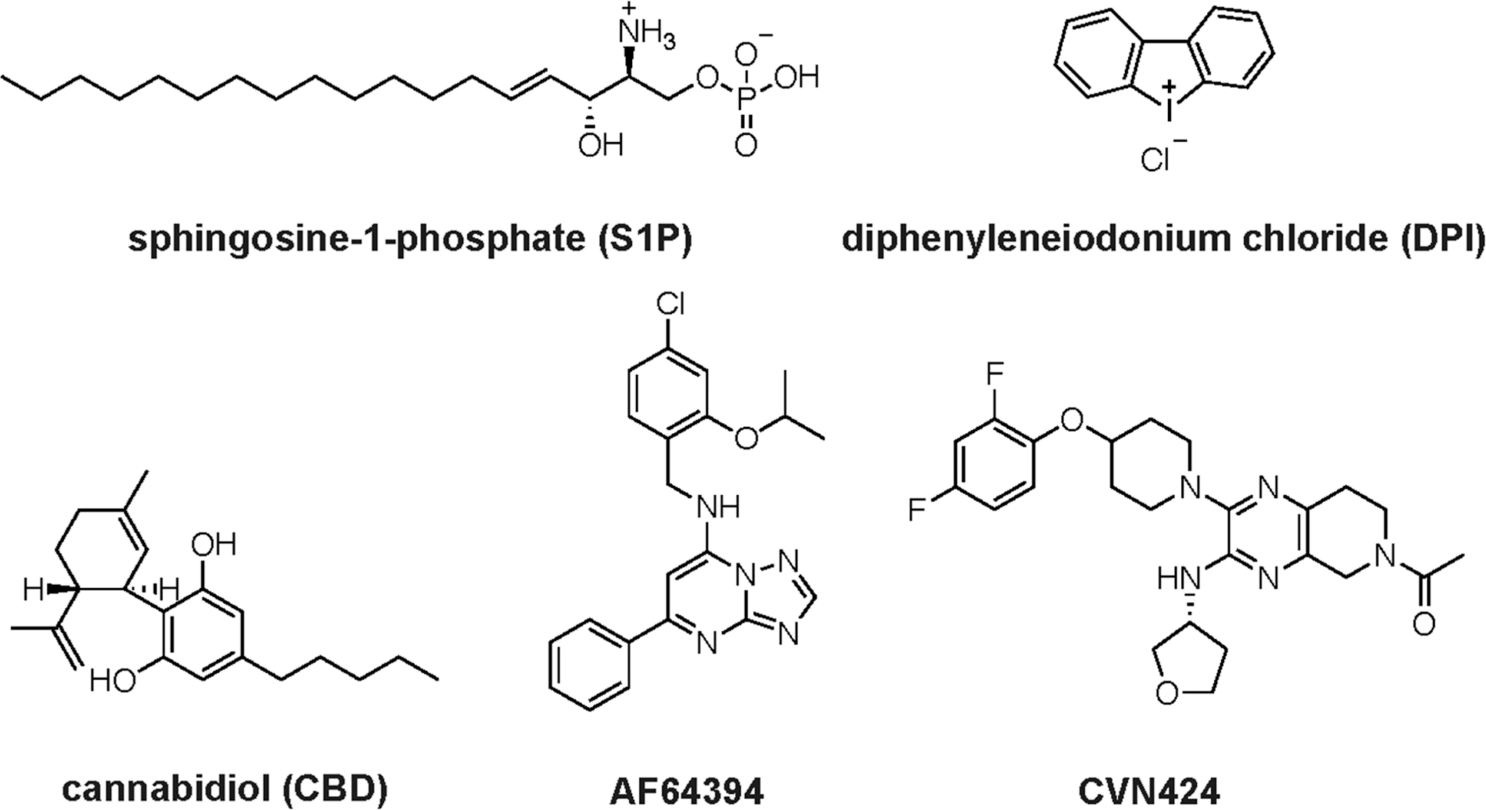
Chemical structures of sphingosine-1-phosphate (S1P), diphenyleneiodonium chloride (DPI), cannabidiol (CBD), AF64394, and CVN424.

Although a couple of ligands for the GPR3/6/12 cluster have been described, GPR3 is still considered orphan. Sphingosine-1-phosphate (S1P, Figure 1), originally put forth as the endogenous GPR3 agonist,^4^ did not elicit a response in later β-arrestin recruitment and cAMP-based studies.^19,20^ Instead, diphenyleneiodonium chloride (DPI, Figure 1) was identified as a synthetic agonist of GPR3.^20^ Although GPR3 and GPR6 were claimed to be novel molecular targets for cannabidiol (CBD, Figure 1),^21,22^ the inhibitory effect of CBD on proinflammatory cytokine production was independent of GPR3 expression.^23^ In 2014, a small-molecule screen against GPR3 identified the first compound that inhibits GPR3 function with submicromolar potency, the inverse agonist AF64394 (Figure 1).^24^ The activity of AF64394 on GPR3 was also recently confirmed in an attempt to develop an HTS-compatible cAMP-based GPR3 assay.^25^

The limited number of available GPR3 ligands and their, in part, contradicting reported activities (see examples for S1P and CBD mentioned above) indicate that we are in need of advanced pharmacological assays detecting compound-GPR3 interactions. Due to the fact that the endogenous ligand for GPR3 is still unknown, the overall portfolio of intracellular signaling pathways promoted by GPR3 remains elusive. Thus, assays detecting compound–GPR3 interactions should preferentially be independent of distinct intracellular signaling pathways. However, all previous GPR3 ligand discovery studies relied on either measuring changes in cAMP or GPR3-arrestin interaction.^4,20,24,25^

Conformational GPCR biosensors that have been optimized for microtiter plate assay formats,^26−30^ as well as direct ligand binding assays based on fluorescence anisotropy or bioluminescence resonance energy transfer (BRET), provide such signaling pathway-independent readouts of compound–receptor engagement.^31−34^ The technique of NanoBRET binding assays, relying on fluorescently labeled ligands and NanoLuciferase (Nluc)-tagged proteins, has gained great importance in recent years as it represents an attractive alternative to radioligand binding assays. It offers the important advantages of monitoring binding kinetics in real time on living cells, working under nonradioactive conditions and has been successfully adapted for numerous GPCRs.^35−45^

In the present study, we have developed a NanoBRET-based GPR3 ligand binding assay by designing, synthesizing, and pharmacologically validating a panel of fluorescently labeled GPR3 ligands. This assay provides the first signaling pathway-independent readout of compound–GPR3 interaction and enables us to discover an allosteric modulatory crosstalk between an AF64394-based fluorescent GPR3 ligand and DPI.

## Results

### Design Rationale

Here, we report the development of a BRET-based binding assay for the orphan receptor GPR3 and the synthesis of 11 fluorescent ligands that are based on either 5-TAMRA or DY-549P1 fluorophores and AF64394 as the pharmacophore scaffold. Previous molecular docking studies of a close analogue of AF64394 to GPR3 suggested that the phenyl ring of AF64394 points toward the extracellular space from the receptor’s binding pocket.^46^ Based on these studies, this moiety was considered the optimal attachment point for a linker to introduce the fluorescent dye. Our interpretation of the docking poses by Bharathi and Roy indicated that an *ortho* attachment of the linker is most likely to prevent clashes with the receptor.^46^ However, to allow for a valid statement about SAR, fluorescent ligands with linkages at the *ortho*, *meta*, and *para* positions of the phenyl ring were designed. In addition to an alkylic linker, PEG-based linkers were chosen due to their improved water solubility, chemical stability, and reduced interaction with the cell membrane.^47^

**Figure 2:**
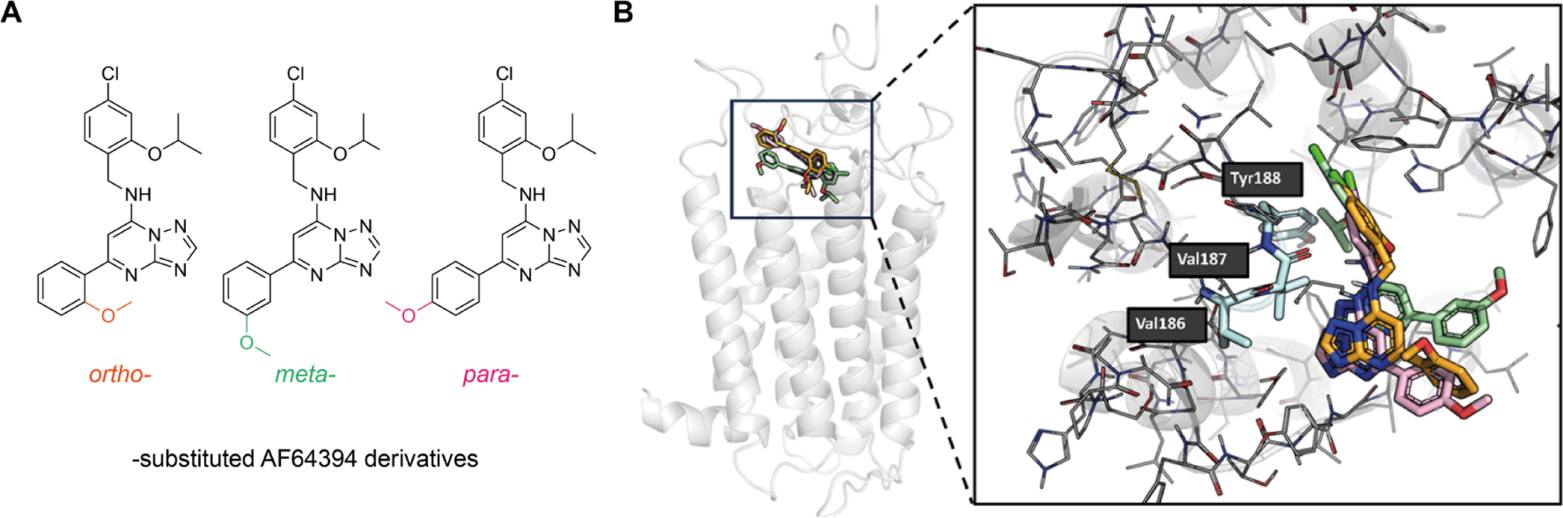
Docking poses of AF64394 derivatives to the inactive-state model of GPR3. (A) Chemical 2D structures of docked AF64394 derivatives with methoxy substituents in *ortho*, *meta*, or *para* positions of the phenyl ring. (B) Side (left) and zoomed-in top view (right) of the docking poses of the three molecules when docked to the GPR3 model developed based on PDB ID 7YXA. Orange: *ortho*-, green: *meta*-, and pink: *para*-substituted AF64394 derivative.

To ensure that the pose of the fluorescent versions of AF64394 and the pose of the molecule depicted in ref (46) (where the isopropoxy and chlorine substituents are positioned in *meta*/*meta* instead of *ortho*/*para* on the benzyl ring) are indeed consistent, we decided to repeat the docking calculations. To account for the linked fluorophores, we docked close analogues of AF64394 with a methoxy group in *ortho*, *meta*, and *para* positions on the phenyl ring, respectively (Figure 2A), to inactive and active models of GPR3. In our docking calculations, we did not consider the full-length linkers, as the extraordinary flexibility of this part would essentially lead to random conformations and poses. Our docking calculations of AF64394 including linker surrogates to the inactive model of GPR3 yielded poses that were similar to the ones obtained by Bharathi et al. (Figure 2B). In our pose, the distance between the secondary amine and the backbone carbonyl of Val187 is larger than for a typical hydrogen bond, even after force-field-based energy minimization of the ligand and the pocket, although the nitrogen does point toward the backbone of Val187. The phenyl moiety carrying the linkers is oriented toward the outside of the receptor binding site, consistent with expectation.

In the synthesis, we employed linkers of different lengths (C_6_-alkyl, four, or six poly(ethylene glycol) units) in order to meet the optimal distance between the fluorophore and pharmacophore. The optimal linker length is distinct for every GPCR and depends on the distance between the ligand binding pocket and the receptor’s extracellular N-terminus. Likewise, the structural flexibility of the employed linker can aid in avoiding unfavorable clashes of the receptor and the fluorescent ligand and can thus have a decisive influence on the affinity of the fluorescent ligand.

The selection of the fluorophore depends on the desired application, i.e., the measurement principle of the assay system. In our case, the chosen fluorophore should be an appropriate BRET acceptor for the energy donor Nluc. 5-TAMRA has been successfully employed as the energy acceptor in numerous NanoBRET studies^32,40,44,45^ and was therefore chosen here for the development of AF64394-based fluorescent ligands of GPR3. Due to the similarity of its excitation and emission spectra, we tested DY-549P1 as an alternative chromophore.

### Chemistry

**Scheme 1:**
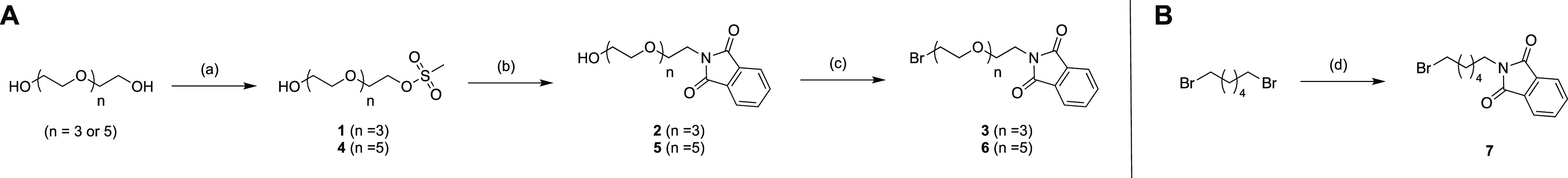
Synthesis of Linker Building Blocks **3**, **6**, and **7** Reagents and conditions: (A) (a) methanesulfonyl chloride, NEt_3_, DCM, (1) 0 °C, 10 h; (2) rt, overnight, 35:44%; (b) potassium phthalimide, DMF, 80 °C, overnight, 66:100%; (c) triphenylphosphine, *N*-bromosuccinimide, DCM, 0 °C, 2 h, 69:64%; (B) (d) potassium phthalimide, DMF, 40 °C, 24 h, 91%.

**Scheme 2:**
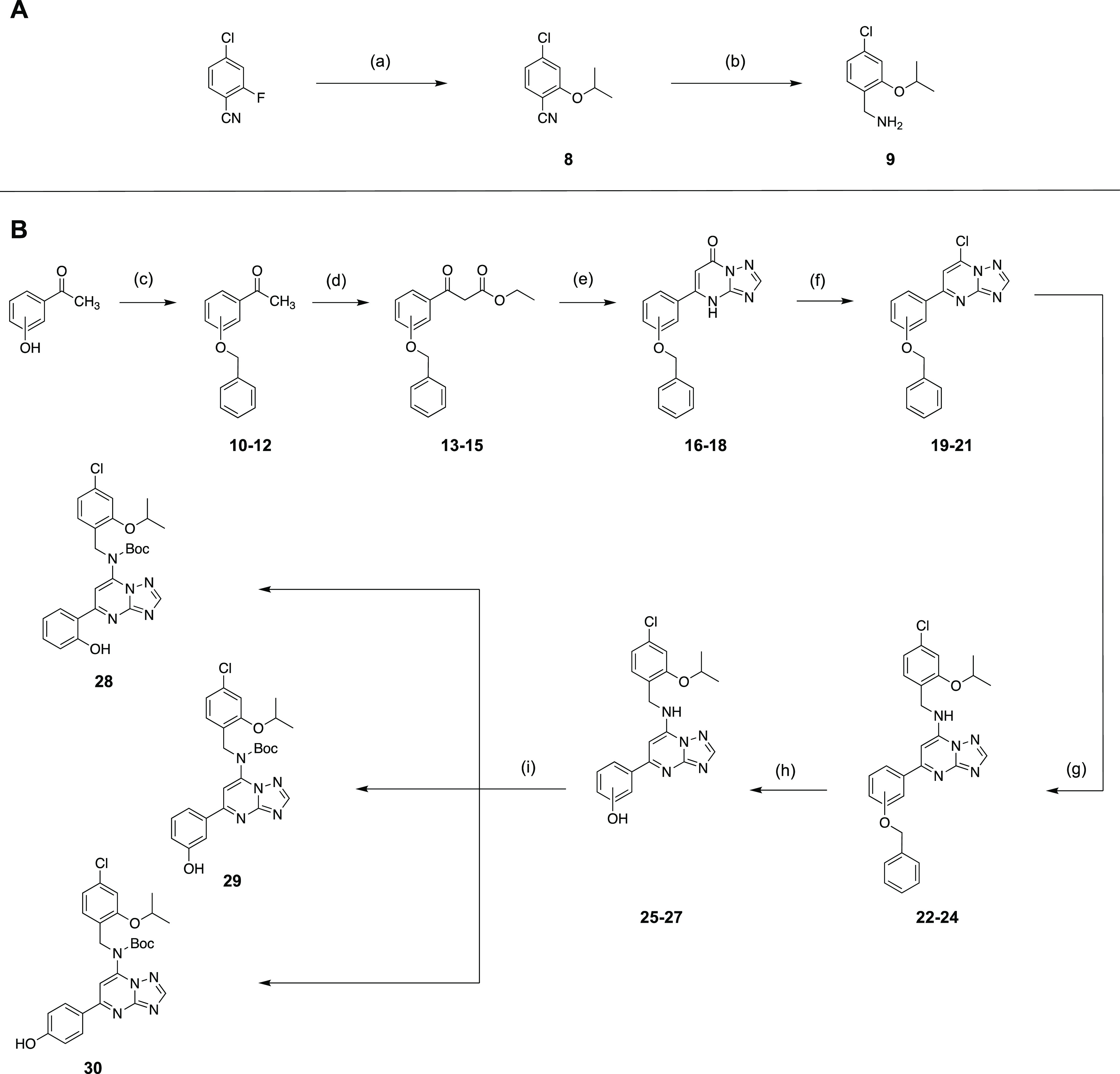
Synthesis of AF64394 Analogues **28–30** as Precursors Reagents and conditions: (A) (a) isopropanol, KHMDS (0.5 M), THF, 0 °C to rt, overnight, 99%; (b) LiAlH_4_, THF, 0 °C, 87%; (B) (c) benzyl bromide, K_2_CO_3_, rt, 4 h, 100:100:100%; (d) NaH, diethyl carbonate, DMF, argon, 0 °C to rt, 8 h, 63:45:66%; (e) 3-amino-1,2,4-triazole, HOAc, argon, 110 °C, 16 h, 31:21:19%; (f) POCl_3_, 105 °C, 1 h, 58:69:63%; (g) **9**, NEt_3_, DCM, argon, rt, 2 d, 35:100:64%; (h) H_2_ (1 atm), Pd/C, MeOH, reflux, TLC monitoring, 91:87:62%; (i) Boc_2_O, NEt_3_, DMAP (cat.), CHCl_3_, 0 °C, overnight, 64:77:80%.

**Scheme 3:**
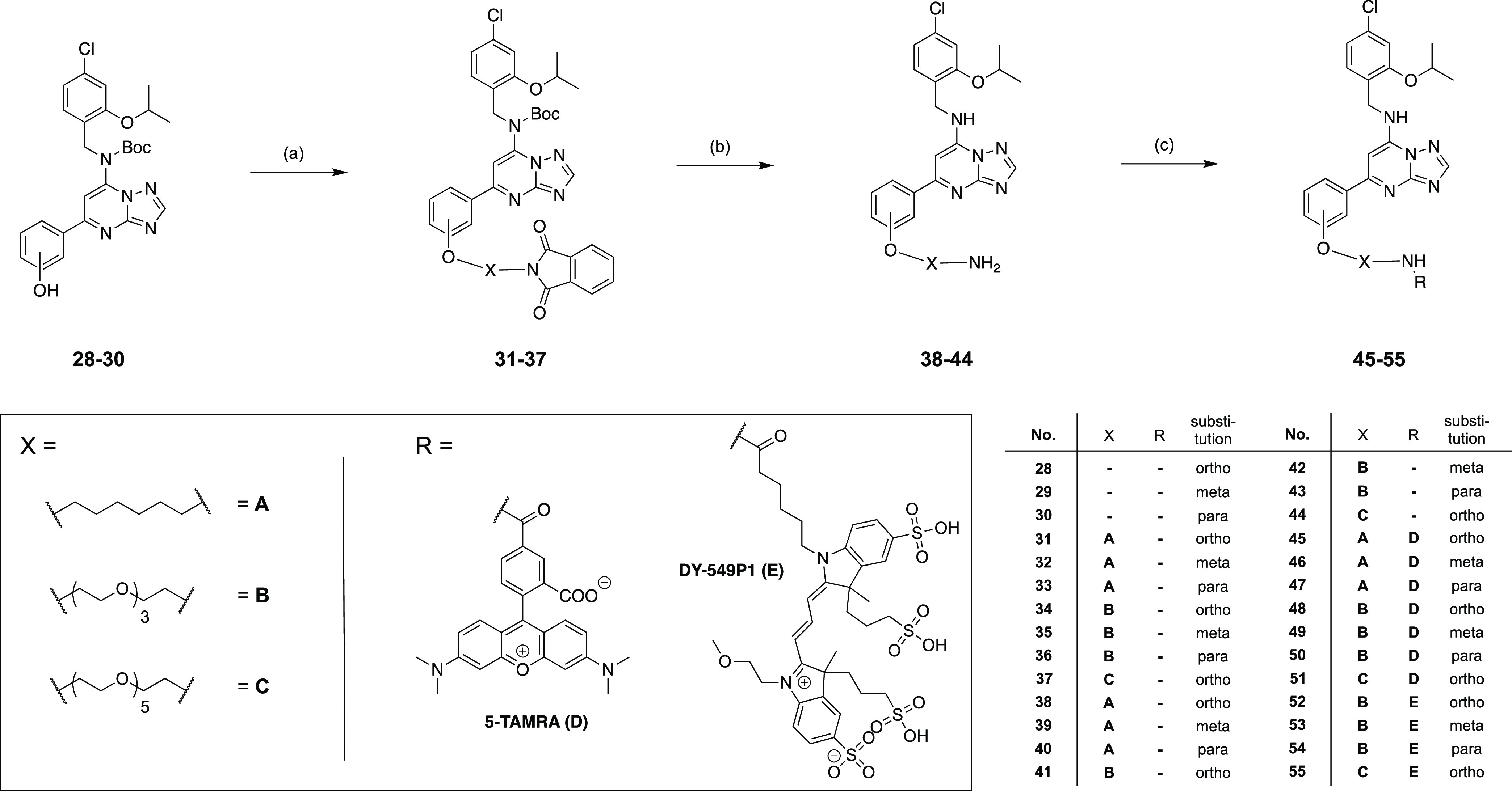
Synthesis of Fluorescent Ligands **45–55** Reagents and conditions: (a) **3**, **6**, or **7**, K_2_CO_3_, DMF, 40 °C, 5–6 d, 83:91:51:27:37:41:85%; (b) N_2_H_4_*x*H_2_O, *n*-butanol, rt, 16 h, 5:9:16:36:8:20:36%; (c) (1) 5-TAMRA NHS ester or DY-549P1 NHS ester, NEt_3_, DMF, rt, 3 h; (2) 10% aq. TFA, rt, 15 min, 42:18:65:86:59:88:66:84:100:97:100%.

The synthesis of the fluorescent ligands can be divided into three parts and is shown in Schemes 1–3, starting with the synthesis of linker building blocks **3**, **6**, and **7** (Scheme 1). For the synthesis of the PEG linkers (Scheme 1A), we started with the reaction of tetraethylene glycol (PEG-4) or hexaethylene glycol (PEG-6) with methanesulfonyl chloride to yield the corresponding methyl sulfonates **1** and **4**. Reaction with potassium phthalimide yielded phthalimides **2** and **5**. This functionality served as a protecting group for the primary amine needed later to introduce the fluorophore. The terminal alcohol group of **2** and **5** was brominated in a nucleophilic substitution reaction using triphenylphosphine and *N*-bromosuccinimide to yield the PEG linker building blocks **3** and **6**. In addition, in a one-step synthesis with 1,6-dibromohexane and potassium phthalimide, the alkyl linker building block **7** was also prepared in a substitution reaction (Scheme 1B).

Due to the implementation of a linker structure via a phenolic group, the AF64394-like pharmacophore had to be prepared in a way contrary to the described syntheses.^24^ Starting from 4-chloro-2-fluorobenzonitrile, the first step was to insert the isopropoxy side chain with the help of the strong non-nucleophilic base KHMDS for the northern part of the molecule (**8**), followed by reduction of the nitrile function to the primary amine **9** (Scheme 2A).^48^ In the reaction to **9**, a slight tendency toward chlorine elimination was observed by MS. The synthesis for the southern part of the molecule (Scheme 2B) was started from 2-, 3-, or 4-hydroxy acetophenone, respectively, to allow linker attachment in *ortho*, *meta*, and *para* positions. In a first step, the phenolic group required for this was benzyl-protected using benzyl bromide to afford **10**–**12**. The subsequent conversion to β-keto-ester compounds **13**–**15** was carried out using sodium hydride and diethyl carbonate.^49^ With the help of 3-amino-1,2,4-triazole under acidic conditions, the formation of the essential [1,2,4]triazolo[1,5-*a*]pyrimidine bicycle and compounds **16**–**18** subsequently occurred. After chlorination with POCl_3_, the 7-chloro-[1,2,4]triazolo[1,5-*a*]pyrimidines **19**–**21** were ready for simple chloride displacement using **9** to afford **22**–**24**. Benzyl deprotection to **25**–**27** and subsequent Boc protection of the amino function yielded AF64394 analogues **28**–**30** ready for linker introduction.

These precursors were subsequently converted to **31**–**37** with linkers **3**, **6**, or **7** (described in Scheme 1), introducing alkyl- and PEG-based side chains of different lengths (Scheme 3). Subsequent hydrazinolysis resulted in cleavage of not only the phthalimide protecting group but, unexpectedly, also the Boc-protecting group, yielding primary amines **38**–**44**. In the final step, in a nucleophilic substitution reaction of the primary amines **38**–**44**, the fluorophores could be introduced using the 5-TAMRA NHS ester and DY-549P1 NHS ester, respectively, yielding fluorescent ligands **45**–**55**.

The fluorescent ligands were further tested for purity (Figures S16–S37) and chemical stability (Figures S38–S41) in aqueous solution or DMSO and showed no degradation within an incubation period of 104 days. The fluorescence properties of **45**–**55** (in DMSO) were determined by recording the excitation and emission spectra shown in Figure S42. The 5-TAMRA-labeled ligands exhibited an excitation maximum at around 564 nm and an emission maximum at around 599 nm, while the DY-549P1-labeled ligands showed maxima at around 568 and 591 nm, respectively (Table S1).

### Pharmacological Characterization

**Figure 3:**
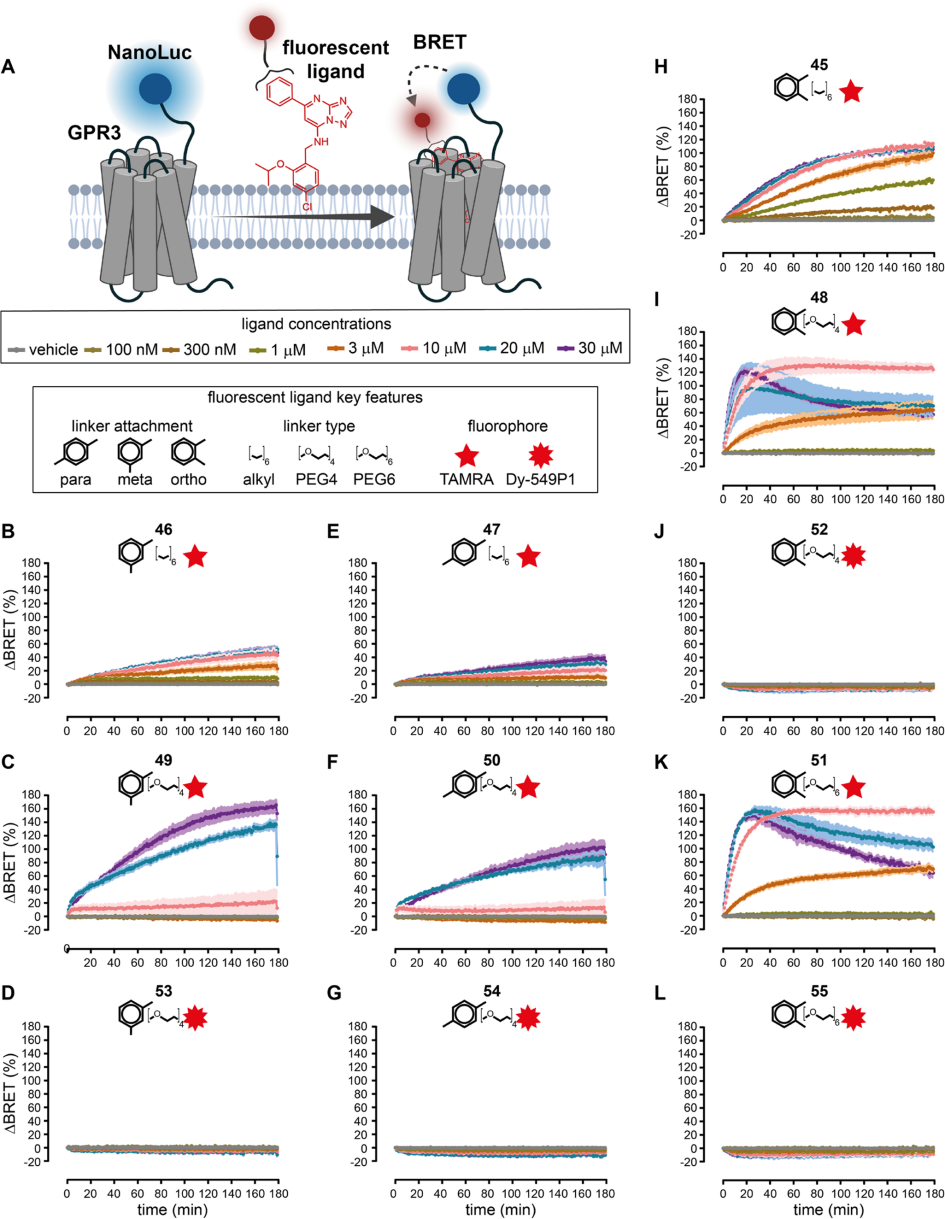
NanoBRET saturation binding time courses of fluorescent AF64394 analogues. (A) Scheme of the assay principle. (B–L) NanoBRET time courses of *meta*- (B–D), *para*- (E–G), and *ortho*-labeled (H–L) fluorescent AF64394 analogues. The indicated fluorescent compound was added to cells stably expressing Nluc-GPR3 before the first time point. Data represent the mean ± SEM of three independent experiments. Experiments were conducted with HEK293A cells stably expressing Nluc-GPR3.

To assess the suitability of synthesized fluorescent AF64394 analogues for BRET-based binding studies to GPR3 (Figure 3A), we developed a HEK293-based cell line stably expressing N-terminally Nluc-tagged GPR3. The luminescence emission spectra obtained with this cell line showed a characteristic intensity peak around 460 nm (Figure S42).

Treatment of these cells with each of the 11 fluorescent AF64394 analogues (Figure 3B–L) revealed time- and concentration-dependent increases in BRET for seven compounds: **46**, **49**, **47**, **50**, **45**, **48**, and **51**. Of note, none of the compounds bearing the DY-549P1 fluorophore (**52**–**55**) showed increasing BRET ratios. In contrast, all 5-TAMRA-labeled compounds induced significant increases in BRET at concentrations of 30 μM or lower. The time courses of **49** and **50** (Figure 3C,F) suggested low ligand affinities since no BRET response was obtained below 10 μM and both compounds were therefore excluded from further characterization. Intriguingly, the addition of 20 and 30 μM of compounds **48** and **51** to Nluc-GPR3 resulted in an initial increase and a subsequent delayed decrease in BRET, suggesting a second pharmacological process following compound–GPR3 binding (Figure 3I,3K).

**Figure 4:**
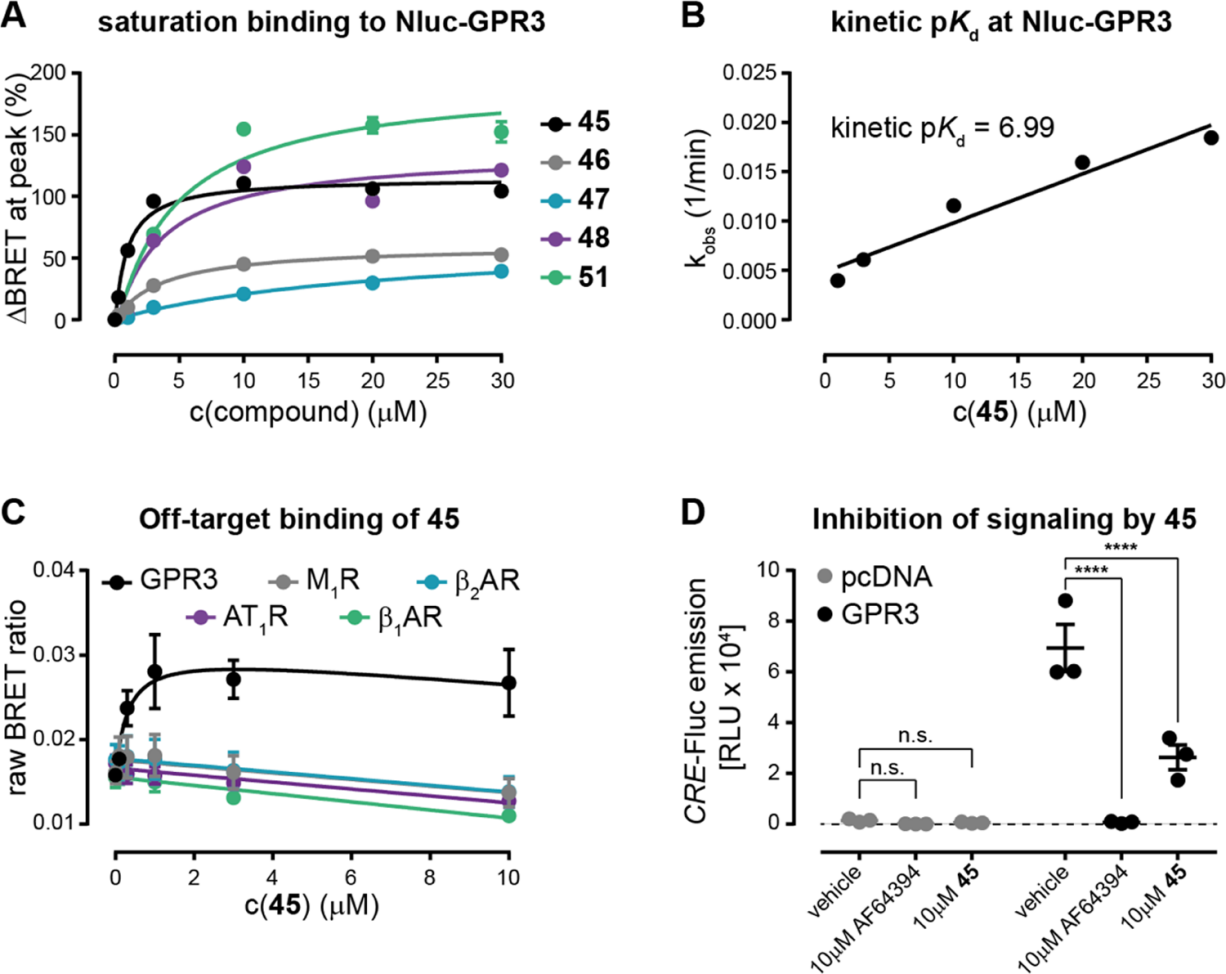
Characterization of **45**. (A) Concentration–response curves of five fluorescent AF64394 analogues (**45**–**48** and **51**) binding to Nluc-GPR3. (B) Kinetic *K*_d_ determination of **45** for binding to Nluc-GPR3. For the kinetic determination of *K*_d_, *k*_on_ and *k*_off_ rates were calculated from linear fitting of the observed on-rates, *k*_obs_, presented in Figure 3 at different ligand concentrations. (C) Concentration–response curves of **45** for binding to transiently expressed Nluc-GPCR constructs. (D) Functional characterization of **45** in a luminescence-based CRE reporter gene assay. Data represent mean ± SEM of three independent experiments conducted in stably Nluc-GPR3 (A, B) or transiently transfected HEK293A cells. Linear vs exponential correlation of the data in panel (C) was tested using the extra-sum-of-squares *F*-test (*p* < 0.05). Significance in panel (D) was assessed using the two-way-ANOVA followed by Dunnett’s multiple comparison (****p < 0.0001).

To obtain a tentative estimation of the binding affinities of the five remaining 5-TAMRA-labeled compounds (**45**, **46**, **47**, **48**, and **51**), their half-maximal effective concentrations were analyzed at the peak of the BRET response recorded in these total binding experiments in which no unspecific binding control was included (Figure 4A and Table 1). These calculations suggested the lowest affinity for compound **47** with an alkylic linker in the *para* position, while **46** (alkylic linker in *meta*), **48** (PEG-4 linker in *ortho*), and **51** (PEG-6 linker in *ortho*) all had very similar pEC_50_ values indicating binding affinities in the low micromolar range. Compound **45** (UR-MB-355), composed of the AF64394 pharmacophore, an alkylic linker in the *ortho* position of the phenyl ring, and 5-TAMRA revealed the highest affinity for binding to stably expressed Nluc-GPR3. The kinetic determination of **45**’s dissociation constant (p*K*_d_ = 6.99, based on the BRET time-course data shown in Figure 3H) further confirmed the submicromolar affinity of this fluorescent GPR3 ligand (Figure 4B) and, therefore, we selected this analogue of AF64394 for subsequent pharmacological characterization. In our docking poses, the methoxy-substituted phenyl ring points toward the extracellular part of the pocket. However, the resolution of docking calculations does not allow us to pinpoint exactly why the *ortho*-substituted **45** shows the highest affinity.

**Table 1:** Binding Affinities of Fluorescent AF64394 Derivatives and GPR3/6/12 Ligands

	pEC_50_ at Nluc-GPR3 (total binding)^a^		p*K*_d_ at GPR3/6/12 (or p*K*_i_ at GPR3) (specific binding)	
compound	mean ± SEM	*N*	mean ± SEM	*N*
**45**	6.05 ± 0.03	3	6.52 ± 0.09^n.s.^/7.18 ± 0.04^n.s.^/7.12 ± 0.04^n.s.^	3/3/3
**46**	5.35 ± 0.07	3	n.d.	
**47**	4.96 ± 0.26	3	n.d.	
**48**	5.47 ± 0.07	3	n.d.	
**49**	<5	3	n.d.	
**50**	<5	3	n.d.	
**51**	5.35 ± 0.04	3	n.d.	
**52**	<4.5	3	n.d.	
**53**	<4.5	3	n.d.	
**54**	<4.5	3	n.d.	
**55**	<4.5	3	n.d.	
AF64394	n.d.		(6.53 ± 0.20)^b^	4
DPI	5.85 ± 0.09^b^	6	n.d.	
			p*K*_i_ at GPR6 (specific binding)	
CVN424			7.88 ± 0.17^b^	5

a Values are derived from the total binding data shown in Figure 4A. “<5” or “<4.5” is indicated for compounds that did not show a response at 10 or 3 μM, respectively, in the time-course experiments shown in Figure 4

b Values derived from coincubation, total binding experiments with 1 μM **45**. n.s.: No statistical difference between p*K*_d_ values as determined by extra-sum-of-squares *F*-test. Values are derived from specific binding experiments using Nluc-β_1_AR as a negative control shown in Figure 5A. Data shown are mean values ± SEM of *N* independent experiments.

To confirm the specificity of the obtained BRET increase, we next incubated increasing concentrations of **45** for 3 h with HEK293A cells transiently transfected with four different N-terminally Nluc-labeled GPCR constructs, which had been validated in previous BRET-based ligand binding studies^32,44^ and compared the resulting BRET ratios to those obtained with HEK293A cells transiently expressing Nluc-GPR3. Here, the BRET ratios obtained with the Nluc-labeled constructs of muscarinic acetylcholine receptor M1 (M_1_R), β-adrenergic receptors β_1_AR and β_2_AR, and type-1 angiotensin II receptor (AT_1_R) resembled a linear concentration–response correlation. Only the data obtained with Nluc-GPR3 showed an exponential increase in BRET with increasing ligand concentrations, confirming the specificity of the BRET response observed with **45** and its selectivity for GPR3 over those of other class A GPCRs (Figure 4C). The decrease in BRET obtained with 3 and 10 μM as compared to 1 μM **45** is probably due to the overproportional quenching of light in the red- versus the blue-shifted portion of Nluc’s emission by 5-TAMRA.

Next, we wanted to investigate whether **45** preserves the inverse agonistic activity of its parent compound, AF64394. Therefore, we quantified cyclic AMP response element (CRE) reporter gene activity in cells transiently transfected with wildtype GPR3 or empty vector (pcDNA) (Figure 4D). The elevated CRE response in vehicle-treated pcDNA- vs GPR3-transfected cells is in accordance with the high level of constitutive GPR3 activity leading to ligand-independent activation of the G_s_-adenylyl cyclase-cAMP signaling cascade. Notably, CRE activity in GPR3-transfected cells was significantly reduced not only in the presence of 10 μM AF64394 but also in the presence of 10 μM **45**. These results demonstrate that **45** still acts as a partial inverse agonist on GPR3. Furthermore, this finding indicates that labeling AF64394 in the *ortho* position of its phenyl ring with 5-TAMRA via an alkylic linker does not abolish the ligand efficacy at GPR3.

Following the confirmation that **45** specifically binds to GPR3, we next probed the suitability of this fluorescent GPR3 ligand for the investigation of ligand binding to the closely related class A orphan GPCRs GPR6 and GPR12 and quantified the affinity of **45** by including Nluc-β_1_AR, allowing us to correct for unspecific binding. Upon transient transfection of either Nluc-GPR3, -GPR6, or -GRP12 and incubation with increasing concentrations of **45**, we obtained very similar exponential concentration–response correlations for all three orphan GPCRs (Figure 5A). Statistical comparison of the computed *K*_d_ values indicated that **45** exhibits a similar binding affinity for GPR3, GPR6, and GPR12 (Table 1). In line with this observation, **45** reduced the constitutive CRE reporter gene response with similar potency in GPR3, GPR6, or GPR12 cotransfected cells (Figure S43). Of note, the p*K*_d_ value measured in these (only specific) saturation binding experiments with GPR3 (mean p*K*_d_ ± SEM = 6.52 ± 0.09) was very similar to that obtained from the kinetic p*K*_d_ (6.99) calculations (Figure 4B).

**Figure 5:**
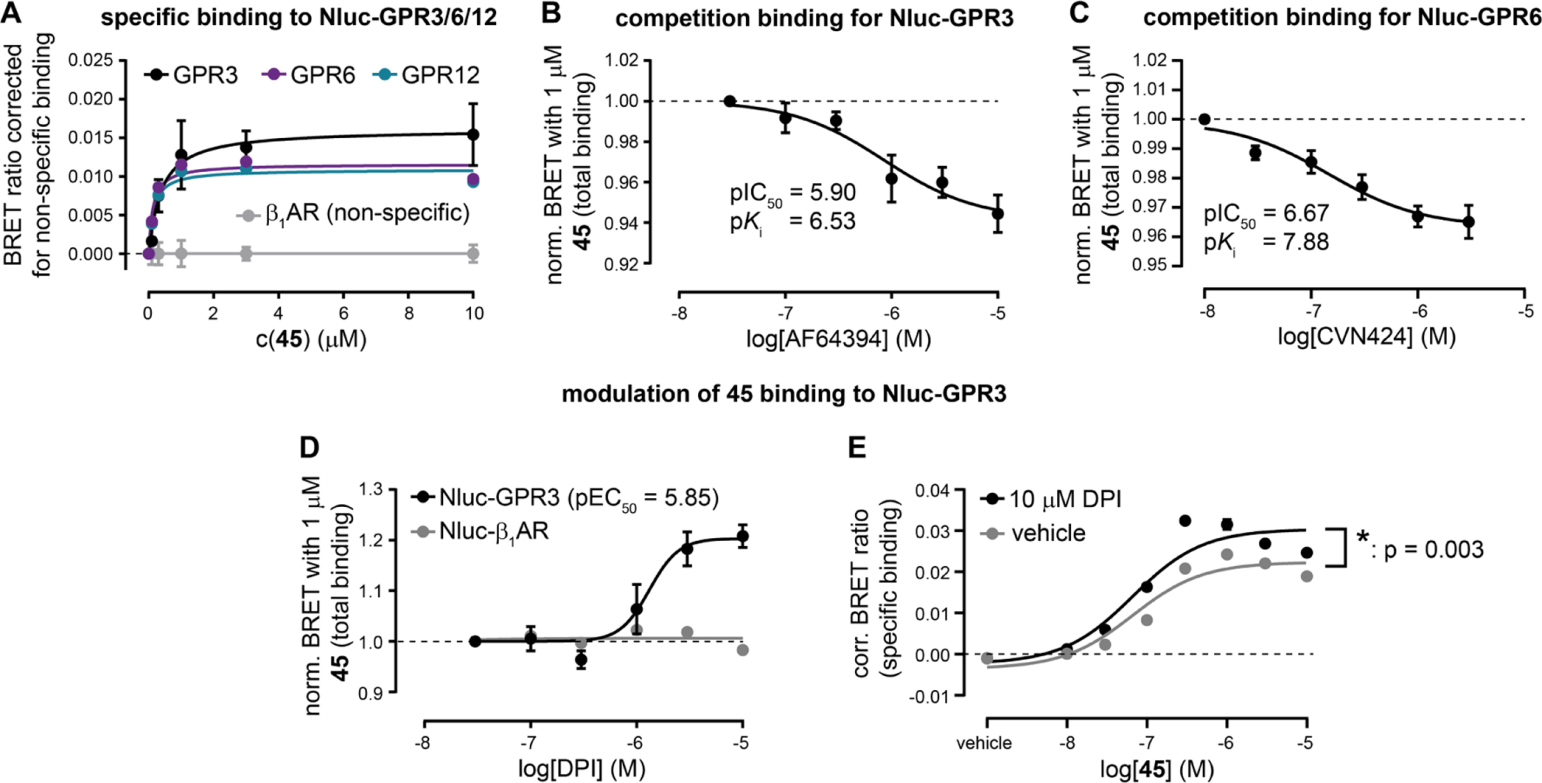
Application of **45** for NanoBRET binding studies with GPR3/6/12. (A) Concentration–response curves of **45** for binding to transiently expressed Nluc-GPR3, Nluc-GPR6, Nluc-GPR12, and Nluc-β_1_AR after 3 h of ligand incubation. The raw BRET ratios obtained with Nluc-β_1_AR (shown in Figure 4C) were averaged and deducted from raw BRET ratios obtained with Nluc-β_1_AR and Nluc-GPR3/6/12 to correct for nonspecific effects. Extra-sum-of-squares *F*-test revealed no statistical difference between the *K*_d_ values obtained with Nluc-GPR3, -GPR6, or -GPR12 (*p* < 0.05). (B) Concentration–response curve of AF64394 inhibiting BRET between Nluc-GPR3 and 1 μM **45**. (C) Concentration–response curve of CVN424 inhibiting BRET between Nluc-GPR6 and 1 μM **45**. (D) Concentration–response curves of DPI enhancing BRET between 1 μM **45** and Nluc-GPR3 but not Nluc-β_1_AR. (E) Concentration–response curves of **45** in the presence or absence of 10 μM DPI. Statistical difference of the BRET maxima and pEC_50_ values was tested using the extra-sum-of-squares *F*-test. Data represent mean ± SEM of three (A), four (B, Nluc-β_1_AR in D), five (C), or six (Nluc-GPR3 in D and E) independent experiments conducted in HEK293A cells transiently (A, C, Nluc-β_1_AR in D) or stably expressing (B, Nluc-GPR3 in D and E) the indicated Nluc-GPCR construct.

Ultimately, we wanted to investigate whether the developed NanoBRET binding assay is suitable to assess in a signaling pathway-independent manner whether unlabeled compounds interact with these orphan GPCRs. Therefore, we conducted competition binding experiments in which cells expressing Nluc-GPR3 or Nluc-GPR6 were coincubated with 1 μM **45**, along with increasing concentrations of either AF64394 or DPI (GPR3) or with the recently developed GPR6 inverse agonist CVN424^50^ (Figure 1).

The experiments with AF64394 revealed a modest (∼5%), yet competitor concentration-dependent, decrease in BRET between **45** and Nluc-GPR3 (Figure 5B). The measured inhibitory constant allowed us, for the first time, to calculate the affinity of AF64394 to GPR3 (p*K*_i_ = 6.53 ± 0.20), which was similar to the previously reported potency of AF64394 in inhibiting constitutive, GPR3-mediated cAMP elevation (pIC_50_ = 7.3).^24^ This affinity is almost identical to that of its fluorescently labeled analogue **45** (p*K*_d_ = 6.52 ± 0.09, see above), indicating that our design strategy had no negative effect on ligand affinity toward GPR3. Likewise, competition experiments of **45** with CVN424 for binding to Nluc-GPR6 revealed a modest, yet CVN424 concentration-dependent decline in energy transfer (Figure 5C). In line with previous radioligand displacement experiments,^50^ CVN424 bound to Nluc-GPR6 with an affinity of about 10 nM (mean ± SEM p*K*_i_ = 7.88 ± 0.17; Table 1).

Surprisingly, our coincubation experiments with DPI revealed a concentration-dependent, receptor-specific increase in BRET between **45** and Nluc-GPR3 (Figure 5D) and the obtained potency of DPI in modulating this binding event (mean pEC_50_ ± SEM = 5.85 ± 0.09) was very similar to its potency in GPR3-mediated cAMP signaling (pEC_50_ = 6.0).^20^ Testing the reverse experimental setup (i.e., fixed concentration of 10 μM DPI coincubated with increasing concentrations of **45**) revealed that DPI does not alter the affinity of **45** for GPR3 but enhances BRET between Nluc and the fluorescent ligand (Figure 5E). These findings indicate that DPI modulates the binding pose of **45** in GPR3 and/or the receptor’s overall conformation in a way that allows more energy transfer to occur from Nluc to 5-TAMRA.

### Compound **45** Exhibits a Binding Mode to GPR3 Distinct from its Parent Compound

The small BRET reduction observed with 1 μM **45** and increasing concentrations of AF64394 led us to the hypothesis that the binding poses of **45** and AF64394 only partially overlap in Nluc-GPR3. Based on our previous computational binding pose predictions of methoxy-substituted AF64394, we reasoned that introducing bulky residues in ECL2 of GPR3 by mutating Val186, Val187, and Tyr188―separately or in combination―to tryptophan (Nluc-GPR3 V186W/V187W/Y188W) should hamper AF64394 and/or **45** binding because of a reduction of the putative ligand binding pocket volume (Figure S45). Additionally, we made use of a recently published cryo-EM structure of the phylogenetically related oGPCR GPR12.^51^ We generated a new homology model of GPR3, which showed a helical extension of TM7 toward the extracellular space, as compared to our previous homology models (Table S4 and Figure S46). By studying this GPR12-based model, we identified an additional potential interaction site of AF64394 and **45** involving aromatic stacking of AF64394’s pyrimidine ring and Tyr280^7.36^ and a hydrogen bond with Thr279^7.35^ (Figure S45). In a second mutagenesis round, we hence mutated both Thr279^7.35^ and Tyr280^7.36^ to alanine in Nluc-GPR3 (Nluc-GPR3 T279A/Y280A).

**Figure 6:**
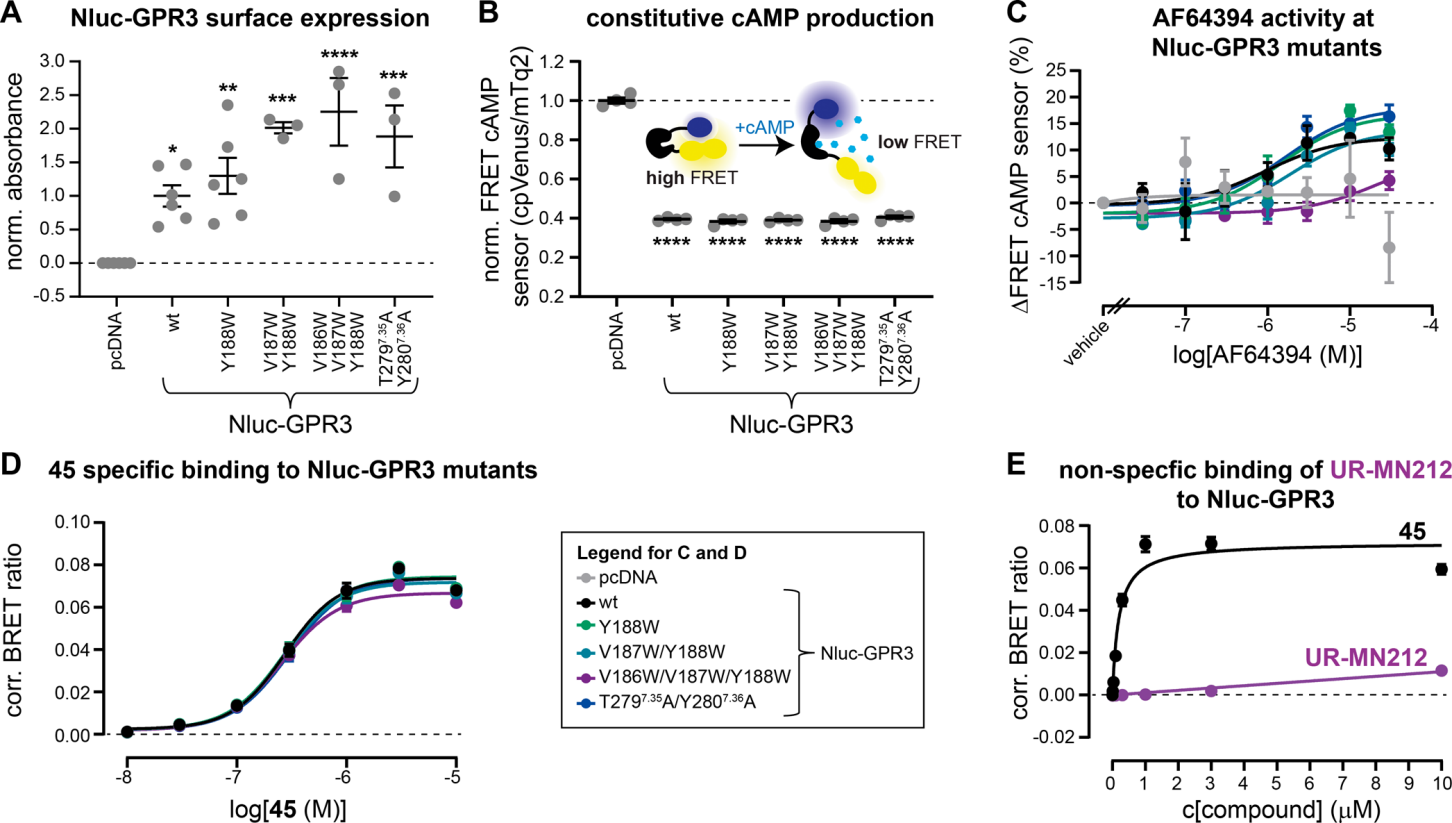
Assessing the binding modes of **45** and AF64394 in GPR3. (A, B) Surface expression levels (A) and constitutive cAMP production (B) of indicated Nluc-GPR3 mutants. (C) FRET concentration–response curves of AF64394 reducing intracellular cAMP levels. (D) Specific binding concentration response curves of **45** to Nluc-GPR3 mutants. (E) Concentration–response curves of **45** and UR-MN212 for binding to transiently expressed Nluc-GPR3. Statistical difference to pcDNA in panels (A, B) was tested using one-way ANOVA followed by Dunnett’s multiple comparison (*p* < 0.05). Statistical significance of the pEC_50_ values in panels (C, D) compared to Nluc-GPR3 wt was tested using extra-sum-of-squares *F*-test. Data represent mean ± SEM of three (A), pcDNA, Nluc-GPR wt, and Nluc-GPR3 Y188W (C, E), four (B, D), or six (Nluc-GPR3 V187W/Y188W, Nluc-GPR3 V186W/V187W/Y188W, and Nluc-GPR3 T279A/Y280A) (A) independent experiments conducted in transiently transfected HEK293A cells.

Prior to the assessment of AF64394/**45** activity at these mutated receptor variants, we confirmed that all mutants were expressed at the surface of intact HEK293 cells at similar levels as wildtype Nluc-GPR3 (Figure 6A). Additionally, we observed that all mutants showed the same levels of constitutive cAMP production (Figure 6B), indicating that the introduced mutations do not affect the signaling capacity of GPR3.

To understand how these mutations affect AF64394 activity, we cotransfected a FRET-based cAMP biosensor^52^ along with either of the mutants and assessed the potency of AF64394 in reducing the constitutive activity of Nluc-GPR3 (reflected by an increase in FRET of the cAMP biosensor). As shown in Figure 6C, only the introduction of three consecutive tryptophan mutations in ECL2 (Nluc-GPR3 V186W/V187W/Y188W) caused a reduction in AF64394 potency, indicating that the reduction of GPR3′s binding pocket volume hampers ligand–receptor engagement. In contrast, none of these mutations affected binding of the fluorescent AF64394 analogue **45** to Nluc-GPR3 (Figure 6D).

These data confirm that AF64394 and **45** exhibit distinct binding modes to GPR3, providing a rationale for the weak displacement of **45** by AF64394 in our competition binding experiments. The chemical fusion of a C6-linker and 5-TAMRA to AF64394 altered the interaction pattern of the AF64394 pharmacophore with GPR3 and we sought to examine whether the C6–5-TAMRA conjugate is the main driver for the BRET increase between **45** and Nluc-GPR3. Thus, we conducted a BRET-based binding experiment with a similar linker–fluorophore fusion compound, UR-MN212^53^ (Table S2), which harbors the dopamine D_2_ receptor pharmacophore spiperone instead of AF64394. In contrast to **45**, incubating Nluc-GPR3-expressing cells with increasing concentrations of UR-MN212 resulted in a linear―thus nonspecific―increase in BRET (Figure 6E), demonstrating that the AF64394 pharmacophore in **45** is essential for the interaction of this fluorescent ligand with GPR3.

## Discussion and Conclusions

Orphan GPCRs provide immense potential for drug discovery but represent challenging targets for pharmacological research. The search for novel ligands of these receptors is often hampered by a limited toolset of appropriate screening assays because the receptor’s complete signaling portfolio remains elusive due to the lack of known endogenous agonists. Thus, signaling pathway-independent readouts of compound–receptor interactions (e.g., ligand binding assays or conformational GPCR biosensors) provide many advantages due to the unbiased nature of the detection principle. GPR3/6/12, in particular, suffers from a very limited assay toolbox. All GPR3-targeting small-molecule screens have been conducted with either cAMP- or β-arrestin-based methods,^20−22,24,25^ although it is still unknown whether GPR3 also stimulates distinct intracellular signaling pathways upon activation.

In the present study, we describe the development of a signaling pathway-independent approach to assess compound–GPR3 interaction in real time and in living cells. Starting from the most potent currently available GPR3 ligand, we designed and synthesized a series of 11 fluorescent analogues of AF64394 for BRET-based ligand binding studies to Nluc-tagged GPR3. In line with computational predictions of AF64394’s binding pose in GPR3, the attachment of a linker with a 5-TAMRA fluorophore to the ligand’s phenyl ring was tolerated, most in the *ortho* variant with an alkylic linker, compound **45**, which exhibits submicromolar affinity for Nluc-GPR3. Compared to attachments of the same linker–fluorophore combination in the *meta* (**46**) or *para* position (**47**), **45** showed 4- and 24-fold higher binding affinity, respectively. Interestingly, replacing the alkylic linker in **45** with a PEG-4 (**48**) or PEG-6 (**51**) led to a similar 4- to 5-fold reduction in affinity, indicating that hydrophilic substituents in this position hamper ligand engagement with GPR3 or enhance the dissociation rates of these compounds. The latter model is supported by a comparison of off-rates calculated for **45** and its PEG-4/6 analogues **48** and **51**, revealing that the alkylic linker indeed reduces the off-rate of fluorescent AF64394 derivatives (Figure S44). Conversely―and in agreement with this hypothesis―competition experiments showed only very modest displacement of **45** by AF64394 from GPR3. This suggests that the structural modifications in **45** serve as a lipophilic anchor that slows the dissociation of this fluorescent ligand from GPR3. Additionally, only partially overlapping ligand binding sites in GPR3 could be the reason for the limited displacement of **45** by AF64394.

An interesting observation from our docking calculations was that using a GPR3 model built based on an active structure, we did not obtain the same pose as shown in Figure 2B. In contrast, we observed one where the phenyl ring carrying the extension points deeper inside the helix bundle in the receptor (Figure S47). Such a pose is incompatible with the attachment of linkers and emphasizes the importance of choosing a conformational state of the receptor that is compatible with the pharmacological effect of the ligand to be docked (agonist, antagonist, or inverse agonist). In conclusion, our docking calculations allowed us to (i) identify a tolerated binding site for the chemical modification of AF64394 and (ii) find, for the first time, the GPR3 residues involved in the binding mode of AF64394. In contrast, our computational approaches failed to predict the distinct binding mode of **45**, probably due to the replacement of the bulky and conformationally flexible **45** molecule by a substantially truncated derivative lacking the C6-alkyl linker and the 5-TAMRA fluorophore in our docking calculations.

Compound **45** further exhibits similar, submicromolar affinity to Nluc-GPR6 and -GPR12. This is in accordance with the high sequence similarity of these three orphan GPCRs (about 60%^5^) but surprising in view of the reported about 100-fold reduced potency of AF64394 at GPR6 and GPR12.^24^ This discrepancy suggests that the attachment of lipophilic moieties in the *ortho* position of the phenyl ring of AF64394 enhances binding to GPR6 and GPR12 and reduces ligand selectivity for GPR3 over GPR6/12.

From a pharmacological perspective, perhaps the most surprising finding of our study is that **45** binds to GPR3 differently than does its parent pharmacophore AF64394. We propose that the binding poses of these compounds only partially overlap, which is supported by our observations that (1) AF64394 only partially displaces **45** from GPR3 and (2) the incorporation of bulky tryptophan residues into ECL2 significantly reduces AF64394 activity but has no effect on **45** binding. It is remarkable that the chemical attachment of a―presumably pharmacologically inert―C6-linker fused to the 5-TAMRA fluorophore has such a dramatic effect on ligand binding. Notably, the same C6-linker-5-TAMRA moiety attached to a different pharmacophore of an unrelated GPCR shows no binding to GPR3.

Collectively, our results indicate that the combination of the AF64394 pharmacophore with the C6-linker and 5-TAMRA fluorophore results in a unique GPR3 ligand with a distinct binding profile. Its only partial displacement by currently available GPR3 (AF64394) and GPR6 (CVN424) ligands may limit the use of **45** for large-scale ligand screening or deorphanization campaigns, but it remains to be assessed in further studies whether **45**’s binding pockets show a greater overlap with future ligands or yet to be identified endogenous ligands of GPR3, 6, and 12. Furthermore, follow-up studies combining this NanoBRET binding assay with blind docking to the GPCR pocketome^54^ could provide valuable insights for future design strategies of orphan GPCR ligands.

Ultimately, our case study highlights the complexity of fluorescent (GPCR) ligand development and demonstrates how the fusion of chemicals with distinct bioactivities can result in mechanistically novel ligands.

## Experimental Section

### Chemistry

Unless otherwise stated, chemicals and solvents were procured from commercial suppliers and used as received. All of the solvents were of analytical grade or distilled prior to use. Anhydrous solvents were stored over a molecular sieve under protective gas. The fluorescent dye 5-TAMRA NHS ester was purchased from Lumiprobe (Hannover, Germany). The fluorescent dye DY-549P1 NHS ester was purchased from Dyomics (Jena, Germany). Tetraethylene glycol, *N*-bromosuccinimide, and diethyl carbonate were purchased from Alfa Aesar (Kandel, Germany). Hexaethylene glycol, di-*tert*-butyl dicarbonate, and trifluoroacetic acid were purchased from abcr (Karlsrube, Germany). 1,6-Dibromohexane, benzyl bromide, and 4-dimethylamino pyridine were purchased from Acros Organics (Geel, Belgium). Acetic acid, isopropanol, triethylamine, methanesulfonyl chloride,1-butanol, 0.5 M potassium bis(trimethylsilyl)amide solution in toluene, sodium hydride, phosphoryl chloride, and hydrazine monohydrate were purchased from Sigma-Aldrich (Taufkirchen, Germany). Dichloromethane, methanol, tetrahydrofuran, dimethylformamide, diethyl ether, chloroform, ethyl acetate, and potassium carbonate were purchased from Fisher Scientific Chemicals (Schwerte, Germany). Potassium phthalimide, 4-chloro-2-fluorobenzonitrile, 2-hydroxy acetophenone, 3-hydroxy acetophenone, 4-hydroxy acetophenone, and 3-amino-1,2,4-triazole were purchased from TCI (Eschborn, Germany). Triphenylphosphine, lithium aluminum hydride, and 1 M hydrochloric acid were purchased from Merck (Darmstadt, Germany). Acetonitrile was purchased from VWR Chemicals (Darmstadt, Germany). Sodium sulfate and ammonia solution 25% were purchased from Carl Roth (Karlsruhe, Germany). Deuterated solvents for nuclear magnetic resonance (NMR) spectroscopy were purchased from Deutero (Kastellaun, Germany). For the preparation of buffers and HPLC eluents, Millipore-grade water was used. Column chromatography was carried out using silica gel 60 (0.040–0.063 mm, Merck (Darmstadt, Germany)). Reactions were monitored by thin layer chromatography (TLC) on silica gel 60 F254 aluminum sheets (Merck) and compounds were detected under UV light at 254 nm by potassium permanganate or ninhydrin staining (0.8 g of ninhydrin, 200 mL of *n*-butanol, 6 mL of acetic acid). NMR spectra (^1^H NMR, ^13^C NMR, and ^19^F NMR, DEPT, 2D NMR) were recorded on a Bruker Avance-300 (7.05 T, ^1^H: 300 MHz, ^13^C: 75.5 MHz, ^19^F: 188), Avance-400 (9.40 T, ^1^H: 400 MHz, ^13^C: 100.6 MHz, ^19^F: 282), or Avance-600 (14.1 T; ^1^H: 600 MHz, ^13^C: 150.9 MHz; cryogenic probe) NMR spectrometer (Bruker, Karlsruhe, Germany). Multiplicities are specified with the following abbreviations: s (singlet), d (doublet), t (triplet), q (quartet), quint (quintet), m (multiplet), br (broad), as well as combinations thereof. High-resolution mass spectrometry (HRMS) was performed on an AccuTOF GCX GC/MS system (Jeol, Peabody, MA) using an EI source or a Q-TOF 6540 UHD LC or GC/MS system (Agilent Technologies, Santa Clara) using an ESI (in the case of LC coupling) or an APCI (in the case of GC coupling) source. Preparative HPLC was performed with a system from Knauer (Berlin, Germany) consisting of two K-1800 pumps and a K-2001 detector or with a Prep 150 LC system from Waters (Eschborn, Germany) consisting of a 2545 binary gradient module, a 2489 UV/visible detector, and a fraction collector III. The following columns were used: Nucleodur 100–5 C18 (5 μm, 250 mm × 21 mm, Macherey-Nagel, Düren, Germany), Kinetex XB-C18 100A (5 μm, 250 mm × 21.2 mm, Phenomenex, Aschaffenburg, Germany), and a Gemini NX-C18 (5 μm, 250 mm × 21 mm; Phenomenex). Solvent flow rates of either 15–20 mL/min (Nucleodur, Kinetex and Gemini columns) or 30 mL/min (Interchim column) at room temperature were employed. A detection wavelength of 220 nm and mixtures of acetonitrile (MeCN) and 0.05–0.1% aqueous TFA were used as mobile phases. MeCN was removed from the eluates under reduced pressure prior to freeze-drying (Christ α 2–4 LD freeze-dryer (Martin Christ, Osterode am Harz, Germany) or ScanVac CoolSafe 4–15L freeze-dryer from Labogene (LMS, Brigachtal, Germany), both equipped with an RZ 6 rotary vane vacuum pump (Vacuubrand, Wertheim, Germany)). Analytical purity and stability control were performed on a 1100 HPLC system from Agilent Technologies equipped with an Instant Pilot controller, a G1312A binary pump, a G1329A ALS autosampler, a G1379A vacuum degasser, a G1316A column compartment, and a G1315B diode array detector (DAD). The column was a Phenomenex Kinetex XB-C18 column (250 mm × 4.6 mm, 5 μm) (Phenomenex, Aschaffenburg, Germany) or a Phenomenex Gemini NX-C18 column (250 mm × 4.6 mm, 5 μm). The oven temperature during HPLC analysis was 30 °C. As a mobile phase, mixtures of MeCN/aqueous TFA or MeCN/aqueous NH_3_ were used. Absorbance was detected at 220 and 543 or 560 nm. The injection volume was 20–80 μL at compound concentrations of 1 mM. The following linear gradient was applied: MeCN/TFA (0.05%) (v/v) 0 min: 10:90, 25 min: 95:5, 35 min: 95:5 (compounds **45**–**51**), or MeCN/NH_3_ (0.05%) (v/v) 0 min: 10:90, 25 min: 95:5, 35 min: 95:5 (compounds **52**–**55**); flow rate: 1.0 mL/min, *t*_0_ (Kinetex XB-C18) = 2.75 min, *t*_0_ (Gemini NX-C18) = 3.00 min (*t*_0_ = dead time). Retention (capacity) factors *k* were calculated from the retention times *t*_R_ according to *k* = (*t*_R_ – *t*_0_)/*t*_0_. The dead time was experimentally determined by injecting a sample of unretained thiourea (200 μM, 10 μL) and recording of the retention time. The purities of the compounds were calculated by the percentage of peak areas in the chromatograms.

Compounds **45**–**55** were characterized by using the following methods: HRMS, ^1^H NMR, ^13^C NMR, and 2D NMR spectroscopies. The purities of target compounds **45**–**55** used for pharmacological investigation were ≥95%. For biological testing, the target compounds (trifluoroacetate or ammonium salts) were dissolved in DMSO to get a final concentration of 10 mM. The tested compounds have been screened for PAINS and aggregation by publicly available filters (http://zinc15.docking.org/patterns/home, http://advisor.docking.org).^55,56^ The compounds have not been previously reported as PAINS or aggregator. None of the data showed abnormalities, e.g., high Hill slopes, which could be a hint for PAINS.^56^

### Synthesis and Analytical Data

#### Preparation of the Fluorescent Ligand **45**

##### 2-(6-Bromohexyl)isoindoline-1,3-dione (**7**)^57^

Potassium phthalimide (3.72 g, 20.09 mmol, 1 equiv) and 1,6-dibromohexane (24.50 g, 100.42 mmol, 5 equiv) were dissolved in 20 mL of anhydrous DMF. The mixture was heated to 40 °C, and stirring was continued for 24 h. The reaction progress was monitored by TLC (*R*_f_ = 0.80, EtOAc/PE 1:3). Subsequently, the solvent was removed under reduced pressure and the crude product was purified by column chromatography (EtOAc/PE 1:20–1:3), yielding a colorless oil (5.68 g, 91%). ^1^H NMR (300 MHz, CDCl_3_) δ 7.83–7.70 (m, 2H), 7.69–7.57 (m, 2H), 3.60 (t, *J* = 7.2 Hz, 2H), 3.31 (t, *J* = 6.8 Hz, 2H), 1.77 (quin, *J* = 6.6 Hz, 2H), 1.61 (quin, *J* = 7.6 Hz, 2H), 1.48–1.20 (m, 4H). ^13^C NMR (75 MHz, CDCl_3_) δ 167.37, 132.87, 131.08, 122.14, 36.78, 32.72, 31.56, 27.38, 26.67, 24.98. HRMS (ESI-MS) *m*/*z*: [M + H^+^] calcd for C_14_H_17_BrNO_2_^+^: 310.0437; found: 310.0439; C_14_H_16_BrNO_2_ (310.19).

##### 4-Chloro-2-isopropoxybenzonitrile (**8**)

4-Chloro-2-fluorobenzonitrile (3.00 g, 19.29 mmol, 1 equiv) was dissolved in 30 mL of THF. Subsequently, isopropanol (1.78 mL, 23.14 mmol, 1.2 equiv) was added to the solution, and the mixture was cooled to 0 °C. 0.5 M potassium bis(trimethylsilyl)amide solution in toluene (KHMDS) (46.28 mL, 23.14 mmol, 1.2 equiv) was added dropwise to the stirring solution. After completion, the ice bath was removed, and stirring was continued overnight. Subsequently, the reaction was quenched with 50 mL of water, and the product was extracted with ethyl acetate. The organic layer was washed with brine and dried over Na_2_SO_4_. The concentration of the organic solvent yielded 4-chloro-2-isopropoxybenzonitrile as a yellow solid (3.73 g, 99%). The product was used without further purification. ^1^H NMR (300 MHz, CDCl3) δ 7.43–7.39 (m, 1H), 6.94–6.88 (m, 2H), 4.58 (m, 1H), 1.36 (d, *J* = 6.1 Hz, 6H). ^13^C NMR (75 MHz, CDCl3) δ 160.42, 140.32, 134.48, 120.98, 114.33, 101.56, 72.54, 21.73. HRMS (EI-MS) *m*/*z*: [M^•+^] calcd for C_10_H_10_ClNO^+^: 195.0450; found: 195.0448; C_10_H_10_ClNO (195.65).

##### 4-Chloro-2-isopropoxyphenylmethanamine (**9**)^24^

**8** (3.73 g, 19.06 mmol, 1 equiv) was dissolved in THF and cooled to 0 °C. Subsequently, lithium aluminum hydride (LiAlH_4_) (2.89 g, 76.26 mmol, 4 equiv) was added stepwise to the solution. The progress of the reaction was monitored with TLC (*R*_f_ (**9**) = 0.00, *R*_f_ (**8**) = 0.80, EtOAc/PE 1:6). After completion, the solution was poured into ice water, and the product was extracted with ethyl acetate. The organic layer was washed with water, dried over Na_2_SO_4_, and concentrated under reduced pressure, yielding the title compound as a yellowish oil (3.33 g, 87%). The product was used without further purification. ^1^H NMR (300 MHz, CDCl_3_) δ 7.21–7.09 (m, 1H), 6.91–6.81 (m, 2H), 4.64–4.48 (m, 1H), 3.74 (s, 2H), 1.58 (bs, 2H), 1.35 (d, *J* = 6.0 Hz, 6H). ^13^C NMR (75 MHz, CDCl_3_) δ 156.28, 133.04, 131.33, 129.35, 120.16, 113.08, 70.35, 42.38, 22.06. HRMS (ESI-MS) *m/*z: [M + H^+^] calcd for C_10_H_15_ClNO^+^: 200.0837; found: 200.0837; C_10_H_14_ClNO (199.68).

#### General Procedure A

The corresponding hydroxy acetophenone (1 equiv) was dissolved in 100 mL of DMF. Potassium carbonate (3 equiv) and benzyl bromide (1.5 equiv) were added to the solution. After addition, the mixture was stirred for 4 h at rt. Subsequently, the reaction was quenched with water (50 mL) and the mixture acidified with 1 M hydrochloric acid. The product was extracted with diethyl ether (3 × 100 mL). The combined organic layers were washed with 1 M hydrochloric acid and dried over Na_2_SO_4_, and the solvent was removed under reduced pressure. The product was used in the next step without further purification. It cannot be ruled out that the crude products still contain traces of DMF, which means that the indicated yield of 100% may be insignificantly lower.

##### 1-(2-(Benzyloxy)phenyl)ethan-1-one (**10**)^58^

The title compound was synthesized from 2-hydroxy acetophenone (4.42 g, 32.49 mmol, 1 equiv), benzyl bromide (8.33 g, 5.79 mL, 48.74 mmol, 1.5 equiv), and potassium carbonate (13.47 g, 97.48 mmol, 3 equiv) in DMF according to general procedure A (*R*_f_ = 0.50 in EtOAc/PE 1:8), yielding a colorless oil (7.35 g, 100%). ^1^H NMR (300 MHz, CDCl_3_) δ 7.81–7.73 (m, 1H), 7.48–7.37 (m, 6H), 7.07–6.98 (m, 2H), 5.17 (s, 2H), 2.62 (s, 3H). ^13^C NMR (75 MHz, CDCl_3_) δ 199.86, 158.04, 136.26, 133.59, 130.48, 128.73, 128.26, 127.59, 120.92, 112.89, 70.72, 32.11. HRMS (EI-MS) *m*/*z*: [M^•+^] calcd for C_15_H_14_O_2_^•+^: 226.09883; found: 226.09916; C_15_H_14_O_2_ (226.28).

#### General Procedure B

Sodium hydride (5 equiv) was suspended in 20 mL of DMF. The flask was flooded with argon and cooled to 0 °C by using an ice bath. The corresponding benzyl-protected hydroxy acetophenone (**10**–**12**, 1 equiv) was dissolved in 180 mL of DMF and slowly added to the suspension over a period of 1 h. After completion, stirring was continued for approximately 15 min until no more gas was released. Then, diethyl carbonate (5 equiv) was added dropwise over a period of 1 h. After the addition, the ice bath was removed, and the mixture was stirred for another 6 h at rt. The synthesis was stopped by adding 50 mL of 1 M hydrochloric acid. The product was isolated by extraction with diethyl ether. The combined organic layers were washed with 1 M hydrochloric acid and dried over Na_2_SO_4_. The crude product was purified by column chromatography (EtOAc/PE 1:5 or EtOAc/PE 1:6). For compounds **13**–**15**, keto–enol tautomerism could be observed in the NMR because especially for the three CH_2_ and the CH_3_ groups, double peaks were clearly visible. In the aromatic region, a clear distinction of the double peaks was not possible. The splitting is indicated accordingly in the NMR data.

##### Ethyl 3-(2-(Benzyloxy)phenyl)-3-oxopropanoate (**13**)^59^

The β-keto-ester **13** was prepared from **10** (7.35 g, 32.48 mmol, 1 equiv), sodium hydride (6.50 g, 162.41 mmol, 5 equiv), and diethyl carbonate (19.19 g, 20.53 mL, 162.41 mmol, 5 equiv) in DMF according to general procedure B (*R*_f_ = 0.8, EtOAc/PE 1:6), yielding a yellowish oil (6.06 g, 63%). ^1^H NMR (300 MHz, CDCl_3_) δ 12.74 (s, 0.1H (enol form)), 7.90–7.82 (m, 1H), 7.49–7.27 (m, 6H), 7.07–6.92 (m, 2H), 6.13 (s, 0.1H (enol form)), 5.15 (s, 2H), 4.22 (q, *J* = 7.1 Hz, 0.2H (keto–enol tautomerism)), 4.08 (q, *J* = 7.1 Hz, 1.8H (keto–enol tautomerism)), 3.99 (s, 1.8H (keto form)), 1.30 (t, *J* = 7.1 Hz, 0.3H (keto–enol tautomerism)), 1.17 (t, *J* = 7.1 Hz, 2.7H (keto–enol tautomerism)). ^13^C NMR (75 MHz, CDCl_3_) δ 193.54, 168.08, 158.21, 135.94, 134.52, 131.11, 128.79, 128.37, 127.60, 126.98, 121.07, 112.98, 92.63, 70.73, 60.90, 50.52, 14.08. HRMS (ESI-MS) *m*/*z*: [M + H^+^] calcd for C_18_H_19_O_4_^+^: 299.1278; found: 299.1281; C_18_H_18_O_4_(298.34).

#### General Procedure C

The corresponding β-keto-ester (**13**–**15**, 1 equiv) and 3-amino-1,2,4-triazole (1 equiv) were weighed in a flask. The flask was set under an argon atmosphere and the compounds were dissolved in 10 mL of acetic acid. The mixture was stirred for 16 h at 110 °C. The reaction was stopped by adding 30 mL of water. The product was extracted with diethyl ether and the crude product was obtained by concentrating the organic phase under reduced pressure. The residue was suspended in 4 mL of methanol and then centrifuged. The supernatant was tipped off. In total, the residue was washed three times with methanol.

##### 5-(2-(Benzyloxy)phenyl)-[1,2,4]triazolo[1,5-*a*]pyrimidin-7(4*H*)-one (**16**)

The title compound was synthesized from **13** (6.06 g, 20.31 mmol, 1 equiv) and 3-amino-1,2,4-triazole (1.71 g, 20.31 mmol, 1 equiv) in acetic acid according to general procedure C (*R*_f_ = 0.85 in DCM/methanol 95:5), yielding **16** as a white solid (2.0 g, 31%). ^1^H NMR (300 MHz, DMSO-*d*_6_) δ 8.29 (s, 1H), 7.59–7.50 (m, 2H), 7.47–7.39 (m, 2H), 7.37–7.24 (m, 4H), 7.12 (t, *J* = 8.0 Hz, 1H), 6.09 (s, 1H), 5.20 (s, 2H). ^13^C NMR (75 MHz, DMSO-*d*_6_) δ 156.43, 156.07, 151.02, 137.11, 132.75, 130.95, 128.85, 128.28, 127.74, 122.35, 121.39, 113.82, 100.26, 70.27. HRMS (ESI-MS) *m*/*z*: [M + H^+^] calcd for C_18_H_15_N_4_O_2_^+^: 319.1190; found: 319.1192; C_18_H_14_N_4_O_2_ (318.34).

#### General Procedure D

The synthesis was performed by dissolving **16**, **17**, or **18** (1 equiv) in 3 mL of phosphoryl chloride, and the mixture was heated to 105 °C for 1 h. Subsequently, the solvent was evaporated and the crude product was purified by column chromatography (EtOAc/PE 1:1).

##### 5-(2-(Benzyloxy)phenyl)-7-chloro-[1,2,4]triazolo[1,5-*a*]pyrimidine (**19**)

**19** was prepared from **16** (1300 mg, 4.08 mmol, 1 equiv) in phosphoryl chloride according to general procedure D (*R*_f_ = 0.75, DCM/methanol 98:2), yielding a yellowish oil (800 mg, 58%). ^1^H NMR (300 MHz, CDCl_3_) δ 8.53 (s, 1H), 8.17 (dd, *J* = 7.8, 1.8 Hz, 1H), 8.04 (s, 1H), 7.42–7.06 (m, 9H), 5.20 (s, 2H). ^13^C NMR (75 MHz, CDCl_3_) δ 161.02, 156.99, 156.17, 155.82, 138.11, 135.93, 132.97, 131.99, 128.79, 128.38, 127.40, 125.30, 121.85, 113.09, 112.61, 71.02. HRMS (ESI-MS) *m*/*z*: [M + H^+^] calcd for C_18_H_14_ClN_4_O^+^: 337.0851; found: 337.0851; C_18_H_13_ClN_4_O (336.78).

#### General Procedure E

A solution of **19**, **20**, or **21** (1 equiv), 4-chloro-2-isopropoxyphenylmethanamine (**9**) (3 equiv), and triethylamine (2.4 equiv) was dissolved in 20 mL of dichloromethane. The reaction was performed under an argon atmosphere and stirred at rt for 2 days. The monitoring of the reaction progress was carried out by TLC (EtOAc/PE 1:1). The solvent was removed by evaporation, and the crude product was purified by column chromatography (EtOAc/PE 1:1–2:1).

##### 5-(2-(Benzyloxy)phenyl)-*N*-(4-chloro-2-isopropoxybenzyl)-[1,2,4]triazolo[1,5-*a*]pyrimidin-7-amine (**22**)

Synthesis of **22** was performed with **19** (388 mg, 1.15 mmol, 1 equiv), (4-chloro-2-isopropoxyphenyl)methanamine (**9**) (690 mg, 3.46 mmol, 3 equiv), and triethylamine (280 mg, 383 μL, 2.77 mmol, 2.4 equiv) in dichloromethane according to general procedure E (*R*_f_ = 0.45, EtOAc/PE 1:1), yielding a yellow oil (200 mg, 35%). ^1^H NMR (300 MHz, CDCl_3_) δ 8.32 (s, 1H), 7.83–7.77 (m, 1H), 7.66–7.57 (m, 1H), 7.43–6.87 (m, 11H), 6.62 (d, *J* = 15.5 Hz, 1H), 5.14 (s, 2H), 4.72–4.53 (m, 3H), 1.36 (d, *J* = 1.9 Hz, 6H). ^13^C NMR (75 MHz, CDCl_3_) δ 160.85, 158.12, 155.24, 154.74, 153.91, 146.48, 138.32, 135.76, 133.94, 128.96, 128.62, 128.37, 127.55, 126.98, 126.55, 123.52, 122.19, 119.42, 119.14, 116.31, 112.33, 84.33, 69.11, 39.90, 20.93. HRMS (ESI-MS) *m*/*z*: [M + H^+^] calcd for C_28_H_27_ClN_5_O_2_^+^: 500.1848; found: 500.1850; C_28_H_26_ClN_5_O_2_ (500.00).

#### General Procedure F

The cleavage of the benzyl protecting group was performed by palladium-on-carbon (Pd/C)-catalyzed hydrogenation. Pd/C (10%) was added to a solution of **22**, **23**, or **24** in 25 mL of methanol. Subsequently, the mixture was heated to reflux under continuous stirring. The permanent TLC monitoring (EtOAc/PE 2:1 or EtOAc/PE 3:1) of the reaction progress is crucial because chlorine is also eliminated from the aromatic ring during hydrogenation, which should be prevented as far as possible. After complete cleavage of the protecting group, the mixture was allowed to cool to room temperature. Afterward, the catalyst was removed by filtration over Celite, and the solvent was evaporated. The crude product was used without further purification.

##### 2-(7-((4-Chloro-2-isopropoxybenzyl)amino)-[1,2,4]triazolo[1,5-*a*]pyrimidin-5-yl)phenol (**25**)

Compound **25** was obtained by catalyzed hydrogenation of **22** (200 mg, 0.400 mmol, 1 equiv) in methanol according to general procedure F (*R*_f_ = 0.40, EtOAc/PE 3:1), yielding a yellow solid (150 mg, 91%). ^1^H NMR (300 MHz, DMSO-*d*_6_) δ 9.02 (t, *J* = 6.4 Hz, 1H), 8.61 (s, 1H), 8.06–7.96 (m, 1H), 7.23–6.79 (m, 7H), 4.77–4.65 (m, 3H), 1.26 (d, *J* = 6.1 Hz, 7H). ^13^C NMR (75 MHz, DMSO-*d*_6_) δ 159.72, 156.38, 155.56, 154.49, 153.89, 148.79, 133.16, 130.40, 129.27, 129.20, 128.96, 126.25, 125.42, 119.43, 118.94, 118.47, 113.86, 85.34, 70.43, 22.31. HRMS (ESI-MS) *m*/*z*: [M + H^+^] calcd for C_21_H_21_ClN_5_O_2_^+^: 410.1378; found: 410.1382; C_21_H_20_ClN_5_O_2_ (409.87).

#### General Procedure G

Boc protection of the secondary aromatic amines was performed by dissolving **25**, **26**, or **27** (1 equiv), triethylamine (1.1 equiv), and catalytic amounts of 4-dimethylamino pyridine (DMAP) in chloroform. After cooling the mixture to 0 °C, di-*tert*-butyl dicarbonate (1.1 equiv) in chloroform was slowly added via a dropping funnel. After complete addition, the ice bath was removed and the mixture was stirred continuously overnight. Subsequently, the solvent was removed and the crude product was purified by column chromatography (EtOAc/PE 2:1).

##### *tert*-Butyl (4-Chloro-2-isopropoxybenzyl)(5-(2-hydroxyphenyl)-[1,2,4]triazolo[1,5-*a*]pyrimidin-7-yl)carbamate (**28**)

The respective N-Boc-protected aromatic amine was obtained from **25** (140 mg, 0.342 mmol, 1 equiv), DMAP (cat.), triethylamine (38 mg, 53 μL, 0.376 mmol, 1.1 equiv), and di-*tert*-butyldicarbonate (82 mg, 0.376 mmol, 1.1 equiv) in a total of 50 mL of chloroform according to general procedure G (*R*_f_ = 0.75, EtOAc/PE 2:1), yielding a colorless oil (122 mg, 64%). ^1^H NMR (300 MHz, CDCl_3_) δ 8.33–8.29 (m, 1H), 7.94–7.85 (m, 1H), 7.49–7.41 (m, 1H), 7.33–7.17 (m, 3H), 6.94–6.83 (m, 2H), 6.67 (d, *J* = 13.6 Hz, 1H), 4.71–4.51 (m, 3H), 1.42 (s, 9H), 1.34 (d, *J* = 1.3 Hz, 6H). ^13^C NMR (75 MHz, CDCl_3_) δ 160.18, 156.30, 155.79, 155.49, 154.90, 151.34, 147.38, 134.98, 131.82, 131.04, 130.83, 130.04, 129.47, 126.36, 124.41, 123.05, 120.38, 112.58, 88.37, 83.81, 42.72, 27.59, 22.07. HRMS (ESI-MS) *m*/*z*: [M + H^+^] calcd for C_26_H_29_ClN_5_O_4_^+^: 510.1903; found: 510.1909; C_26_H_28_ClN_5_O_4_ (509.99).

#### General Procedure H

The coupling of the respective linkers (**3**, **6**, or **7**) with pharmacophores **28**, **29**, or **30** was performed by a nucleophilic substitution reaction in DMF. **28, 29**, or **30** (1 equiv), **3**, **6**, or **7** (5 equiv), and potassium carbonate (5 equiv) were dissolved in DMF. The mixture was stirred continuously at 40 °C for 5–6 days. Monitoring of the reaction was constantly performed by TLC (EtOAc/PE 1:1 or EtOAc/PE 2:1). After completion, the solvent was removed under reduced pressure and the crude product was purified by column chromatography (DCM/methanol 99:1–95:5 (**Method A**) or EtOAc/PE 2:1–6:1 (**Method B**)).

##### *tert*-Butyl (4-Chloro-2-isopropoxybenzyl)(5-(2-((6-(1,3-dioxoisoindolin-2-yl)hexyl)oxy)phenyl)-[1,2,4]triazolo[1,5-*a*]pyrimidin-7-yl)carbamate (**31**)

The synthesis of **31** was carried out with **28** (150 mg, 0.294 mmol, 1 equiv), **7** (456 mg, 1.471 mmol, 5 equiv), and potassium carbonate (110 mg, 0.794 mmol, 5 equiv) in 10 mL of DMF according to general procedure H (method A) (*R*_f_ = 0.20, EtOAc/PE 1:1), yielding a colorless oil (180 mg, 83%). ^1^H NMR (300 MHz, CDCl_3_) δ 8.34 (s, 1H), 7.93–7.88 (m, 1H), 7.86–7.80 (m, 2H), 7.75–7.67 (m, 2H), 7.49–7.41 (m, 1H), 7.39–7.32 (m, 1H), 7.25–7.20 (m, 1H), 7.09 (d, *J* = 8.6 Hz, 1H), 6.88–6.81 (m, 2H), 6.57 (s, 1H), 5.17 (s, 2H), 4.56–4.41 (m, 1H), 3.75–3.62 (m, 4H), 1.82–1.59 (m, 4H), 1.39 (s, 13H), 1.14 (d, *J* = 6.0 Hz, 6H). ^13^C NMR (75 MHz, CDCl_3_) δ 168.45, 159.46, 156.42, 153.85, 151.18, 149.84, 148.77, 134.44, 133.96, 132.12, 131.52, 131.06, 130.88, 129.88, 126.39, 123.44, 123.22, 123.06, 120.36, 113.22, 93.94, 83.81, 70.53, 50.90, 49.56, 37.78, 28.50, 27.56, 26.45, 26.34, 21.70. HRMS (ESI-MS) *m*/*z*: [M + H^+^] calcd for C_40_H_44_ClN_6_O_6_^+^: 739.3005; found: 739.3010; C_40_H_43_ClN_6_O_6_ (739.27).

#### General Procedure I

The corresponding phthalimides (**31**–**37**) were deprotected by hydrazinolysis. Therefore, **31**–**37** (1 equiv) and hydrazine monohydrate (5 equiv) were dissolved in 1-butanol. The mixture was stirred at rt for 16 h. After cooling the mixture to 0 °C using an ice bath, the precipitated phthalhydrazide was filtered off over Celite. The solvent was evaporated, and purification was performed with preparative HPLC (MeCN/0.1% aqueous NH_3_ or MeCN/0.5% aqueous TFA).

##### 5-(2-((6-Aminohexyl)oxy)phenyl)-*N*-(4-chloro-2-isopropoxybenzyl)-[1,2,4]triazolo[1,5-*a*]pyrimidin-7-amine Dihydrotrifluoroacetate (**38**)

The title compound was synthesized from **31** (150 mg, 0.203 mmol, 1 equiv) and hydrazine monohydrate (50.79 mg, 49 μL, 1.015 mmol, 5 equiv) in 5 mL of 1-butanol according to general procedure I (*R*_f_ = 0.15 in DCM/methanol/NH_3_ conc. 50:50:1), yielding a colorless oil (7.92 mg, 5%). ^1^H NMR (300 MHz, CD_3_OD) δ 8.44 (s, 1H), 7.83 (d, *J* = 7.8 Hz, 1H), 7.41–7.25 (m, 2H), 7.06–6.85 (m, 5H), 5.28 (s, 2H), 4.64–4.52 (m, 1H), 3.89 (t, *J* = 7.5 Hz, 2H), 2.89 (t, *J* = 7.8 Hz, 2H), 1.89–1.75 (m, 2H), 1.70–1.58 (m, 2H), 1.48–1.39 (m, 4H), 1.06 (d, *J* = 6.0 Hz, 6H). ^13^C NMR (75 MHz, CD_3_OD) δ 160.94, 159.38, 156.63, 155.86, 151.32, 150.46, 132.74, 129.23, 129.19, 127.92, 124.21, 120.94, 120.10, 119.11, 118.17, 117.84, 112.66, 90.74, 69.54, 51.11, 39.18, 27.47, 27.10, 25.85, 25.75, 20.70. HRMS (ESI-MS) *m*/*z*: [M + H^+^] calcd for C_27_H_34_ClN_6_O_2_^+^: 509.2426; found: 509.2433; C_27_H_33_ClN_6_O_2_*x*C_4_H_2_F_6_O_4_ (737.10).

#### General Procedure J

The corresponding fluorophore (5-TAMRA NHS ester (1 equiv) or DY-549P1 NHS ester (1 equiv)) was weighed into an Eppendorf reaction vessel. The amine precursors (**38**–**44**, var. eq) and triethylamine (11 equiv) were dissolved in DMF (100 μL) and added to the vessel. The solution was vigorously shaken for 3 h at rt in the dark. Subsequently, the reaction was stopped by adding 10% aq. TFA (100 μL) and purified by preparative HPLC (MeCN/0.05% aq. TFA (**Method A**) or MeCN/0.1% aqueous NH_3_ (**Method B**)). Due to the insufficient amount (<0.5 mg) of DY-549P1 fluorescent ligands, **52**–**55** NMR could not be measured. The identity was confirmed by HRMS.

##### 2-(3,6-Bis(dimethylamino)xanthylium-9-yl)-5-((6-(2-(7-((4-chloro-2-isopropoxybenzyl)amino)-[1,2,4]triazolo[1,5-*a*]pyrimidin-5-yl)phenoxy)hexyl)carbamoyl)benzoate Hydrotrifluoroacetate (**45**)

The title compound was prepared from precursor **38** (3.96 mg, 0.0054 mmol, 1.3 equiv), 5-TAMRA NHS ester (2.23 mg, 0.0042 mmol, 1 equiv), and triethylamine (6.19 μL, 0.0465 mmol, 11 equiv) according to general procedure J (using method A for purification), yielding a fluffy purple solid (1.83 mg, 42%). RP-HPLC: 97% (*t*_R_ = 18.31 min, *k* = 5.10). ^1^H NMR (400 MHz, CD_3_OD) δ 8.74 (d, *J* = 1.8 Hz, 1H), 8.38 (s, 1H), 8.20 (dd, *J* = 7.9, 1.9 Hz, 1H), 7.83 (d, *J* = 8.1 Hz, 1H), 7.47 (d, *J* = 8.0 Hz, 1H), 7.36–7.28 (m, 2H), 7.12–6.96 (m, 7H), 6.94–6.88 (m, 4H), 5.32 (s, 2H), 4.62–4.52 (m, 1H), 3.88 (t, *J* = 7.7 Hz, 2H), 3.46 (t, *J* = 6.6 Hz, 2H), 3.30 (s*, 12H, concealed), 1.87–1.79 (m, 2H), 1.72–1.65 (m, 2H), 1.52–1.46 (m, 4H), 1.05 (d, *J* = 6.0 Hz, 6H). HRMS (ESI-MS) *m*/*z*: [M + H^+^] calcd for C_52_H_54_ClN_8_O_6_^+^: 921.3849; found: 921.3848; C_52_H_53_ClN_8_O_6_*x*C_2_HF_3_O_2_ (1035.52).

### Homology Modeling

Templates were chosen based on the alignment in GPCRdb.^60^ Accordingly, sphingosine-1-phosphate receptor 5 (S1P_5_) with 31% identity and 46% similarity to GPR3 was chosen as a template. Homology modeling was performed in MODELER.^61^ Two models, one active based on PDB ID 7ew1, and one inactive based on PDB ID 7yxa, were built. Five hundred model variants were generated based on each template and, to find representative models, they were clustered in Chimera.^62^ The best five representative models were selected, and their quality was investigated by considering the DOPE (discrete optimized protein energy) score, MODELER objective function value, RMSD (compared to the template), and normalized DOPE values (*z*-score). Model statistics are provided in Table S4.

### Docking Calculations

The 3D structures of the small molecules in db2 format were generated in tldr.docking.org using the newBuild3D module. Docking calculations were performed in DOCK3.7.^63^ The obtained poses were then minimized using a variant of MMFF94 (Merck Molecular force field), MMFF94x,^64^ in the Molecular Operating Environment (MOE) software.^65^ In the minimization step, the ligand and surrounding binding pocket residues were subjected to minimization. PyMOL (The PyMOL Molecular Graphics System, Version 2.0, Schrödinger, LLC) was used for the visualization of the poses.

### Plasmids and Molecular Cloning

The expression plasmids encoding N-terminally HA-tagged GPR3 (Catalog No. #GPR003TN00), GPR6 (Catalog No. #GPR0060000), and N-terminally HA-tagged GPR12 (Catalog No. #GPR012TN00) all encoded on a pcDNA3.1+ vector were obtained from the cDNA resource center (cdna.org). The Nluc insert was amplified with overhangs from α_2A_AR-Nluc/HaloTag^26^ and inserted between the HA-tag sequence and GPR3 using prolonged overlap extension PCR. Nluc-GPR6 and Nluc-GPR12 were cloned using restriction enzyme digestion and ligation. Nluc-AT_1_R, Nluc-β_1_AR, Nluc-β_2_AR, and Nluc-M_1_R were described previously.^44^ The plasmid encoding CRE-Fluc (pGL4.29[luc2P/CRE/Hygro]) was obtained from Promega. The H187-EPAC-FRET sensor^52^ was kindly provided by Jalink (The Netherlands Cancer Institute, Amsterdam, The Netherlands). All constructs were verified by sequencing (Eurofins genomics).

### Reagents

Poly-d-lysine was obtained from Sigma-Aldrich (Merck KGaA). Dulbecco’s Modified Eagle’s Medium (DMEM) and G-418 (Geneticin) were from Gibco. Diphenyleneiodonium chloride (DPI) and AF64394 were purchased from Tocris (Bio-Techne). CVN424 was obtained from MedChemExpress (MCE). The Nluc substrates furimazine (Catalog No. N1572) and vivazine (Catalog No. N2581) were from Promega (Madison). White-wall, white-bottomed 96-well and black-wall, black-bottomed 96-well microtiter plates were from Brand.

### Cell Culture

HEK293A was used for the experiment upon transient transfection and grown in Dulbecco’s modified Eagle’s medium (DMEM) supplemented with 2 mM glutamine, 10% fetal calf serum, streptomycin (0.1 mg/mL), and penicillin (100 U/mL) at 37 °C with 5% CO_2_. For the generation of stable Nluc-GPR3 cells, HEK293A cells grown in T75 flasks were transfected at a confluence of 40–50% with 1 μg of DNA. To select for stably expressing cells, transfected cells were cultured with 2000 μg/mL of G-418 and maintained in fully supplemented DMEM containing 500 μg/mL G-418. The absence of *Mycoplasma* contamination was routinely confirmed by PCR.

### Transient Transfection and Plating

Resuspended cells (300,000 cells/mL) were transfected in suspension with a total of 1 μg of DNA/mL suspension using Lipofectamine 2000 (Thermo Fisher Scientific; 2 μL of Lipofectamine 2000 per μg DNA). For reporter gene and cAMP experiments, resuspended cells were transfected with 500 ng of pcDNA, wildtype GPR3 or Nluc-GPR3 constructs along with 500 ng of *CRE*-Fluc plasmid or the FRET-based cAMP sensor H187, respectively, per mL of cell suspension. Cells mixed with the transfection reagents were seeded onto poly-d-lysine-precoated 96-well plates and grown for 48 h at 37 °C with 5% CO_2_. White plates were used for BRET and reporter gene experiments and black plates for experiments with the FRET cAMP sensor. Stable Nluc-GPR3-expressing cells were seeded 24 h prior to the experiment at a density of 800,000 cells/mL into white 96-well plates.

### Recording of the Nluc-GPR3 Luminescence Spectrum

HEK293A cells stably expressing Nluc-GPR3 were seeded as described above. Luminescence emission was recorded between 400 and 700 nm with 2 nm resolution in Hank’s balanced salt solution (HBSS) upon the addition of 1:1000 furimazine dilution. All experiments were conducted using a CLARIOstar plate reader (BMG, Ortenberg, Germany), and spectra were normalized to the donor emission peak.

### Recording of Fluorescence Excitation and Emission Spectra of 5-TAMRA- and DY-549P1-Labeled AF64394 Analogues

The fluorescent compounds were diluted in DMSO to a concentration of 20 μM. 100 μL portion of each ligand preparation or DMSO (blank) was added to a well of a black 96-well plate, and the excitation and emission spectra were recorded using a CLARIOstar plate reader with the following measurement settings: Excitation scan: emission wavelength 610–10 nm; excitation range 500–585 nm with 1 nm increments; gain 800. Emission scan: excitation wavelength 522 nm; emission range 550–650 nm with 1 nm increments; gain 800.

### NanoBRET-Based Ligand Binding Experiments

HEK293A transiently or stably expressing the indicated Nluc-GPCR constructs were grown for 48 or 24 h, respectively, and washed once with HBSS. For time-course saturation binding experiments, cells were then incubated with 1:100 vivazine solution (in HBSS) for 2 h, stimulated with fluorescent compounds or vehicle control, and the BRET ratios were recorded using a Spark multimode reader (Tecan, Männedorf, Switzerland) for 180 min with a temporal resolution of one data point per minute. For end point BRET measurements, fluorescent compounds were incubated with or without competitor (AF64394)/modulator (DPI) for 3 h in HBSS. Following this incubation period, 1:1000 furimazine (in HBSS) was added to the wells and three BRET reads were recorded and averaged. All experiments were conducted at 37 °C. Nluc emission intensity was quantified between 430 and 530 nm with an integration time of 50 ms. TAMRA and DY-591P1 emissions were quantified between 565 and 620 nm with an integration time of 50 ms.

### FRET-Based cAMP Measurements

Transfected cells grown in black 96-well plates were washed twice with 100 μL of HBSS 48 h after transfection and incubated with HBSS. Three baseline FRET reads were recorded and averaged prior to the addition of serial dilutions of AF64394, **45** or vehicle control, and subsequent FRET reads. All experiments were conducted at 37 °C. Cells were excited using a 430:10 nm filter combined with 458 and 504 nm long-pass filters to separate excitation from donor and acceptor emission light. FRET donor emission was quantified with a 480:10 nm filter. FRET acceptor emission was quantified using a 530:10 nm filter.

### CRE Reporter Gene Assay

Transfected cells grown in 96-well plates were washed with 100 μL of HBSS 24 h after transfection and incubated for another 24 h in fetal bovine serum (FBS)-free, fully supplemented DMEM containing either vehicle control, 10 μM AF64394, or 10 μM **45**. The day of the experiment, cells were washed with HBSS and lysed in 30 μL of Promega’s dual luciferase passive lysis buffer (15 min at room temperature). Then, 30 μL of luciferase assay reagent (LARII) was added to each well and CRE–dependent firefly luciferase (Fluc) intensity was measured at 37 °C using a CLARIOstar microplate reader (580:80 nm; 800 ms integration time).

### Data Analysis

FRET and BRET ratios were defined as acceptor emission/donor emission. The first ratio of a time-course experiment was defined as Ratio_basal_. To quantify ligand-induced changes, ΔFRET and ΔBRET were calculated for each well as a percent over basal ([(Ratio_stim_ – Ratio_basal_)/Ratio_basal_] × 100). Subsequently, the average ΔFRET/BRET of vehicle control was subtracted. Data from FRET/BRET experiments were fitted using a three-parameter fit. Linear vs exponential correlation for the data obtained from off-target binding experiments (Figures 4C and 6E) was tested using the extra-sum-of-squares *F*-test (*p* < 0.05). For competition experiments of **45** with AF64394, DPI, or CVN424, the preferred model (three- vs four-parameter log[agonist] vs response fit) was determined using an extra-sum-of-squares *F*-test. Based on the outcome of this test, a three-parameter fit was used to fit the competition data with AF64394 and CVN424 (Figure 5B,C) and a four-parameter fit (variable hill slope) was used to fit the modulation of binding of **45** to Nluc-GPR3 by DPI (Figure 5D). *K*_d_, EC_50_, and IC_50_ values were first determined for individual experiments, then transformed into p*K*_d_, pEC_50_, and pIC_50_ values, and ultimately pooled to calculate mean ± SEM of all biological replicates. Likewise, *K*_i_ values were first calculated for individual experiments using the Cheng–Prusoff equation (*K*_i_ = IC_50_/(1 + c(**45**)/*K*_d_(**45**))), then transformed into p*K*_i_ values and pooled to calculate mean ± SEM p*K*_i_ of all biological replicates. For the kinetic determination of the dissociation constant *K*_d_, *k*_on_ and *k*_off_ rates were calculated from a linear fit of the observed on-rates, *k*_obs_, at different ligand concentrations. The *y*-intercept of the linear regression represents *k*_off_, while *k*_on_ is represented by the slope of the fit. *K*_d_ was then calculated by using the equation *K*_d_ = *k*_on_/*k*_off_. Data shown are mean values ± SEM of *N* independent experiments. Reporter gene experiments were analyzed by plotting the raw intensity in the Fluc emission channel and statistical significance was tested using two-way ANOVA followed by Dunnett’s multiple comparison (****p < 0.0001). Data were analyzed using Prism 5.0 software (GraphPad, San Diego, CA).

## Data Availability

All data are contained within the article.

## Supporting Information Available

Preparation of linkers **3** and **6**; preparation of fluorescent ligands **46**–**55**; chemical structures of **7**–**10**, **13**, **16**, **19**, **22**, **25**, **28**, **31**, **38**, and **45**; NMR spectra of compounds **38**–**44** and **45**–**51**; RP-HPLC purity control of compounds **45**–**55**; RP-HPLC stability control of compounds **46** and **48**; fluorescence properties; activities of AF64394 and **45** in a CRE reporter gene assay with GPR3, GPR6, and GPR12; comparison of dissociation rates or *ortho*-labeled fluorescent AF64394 analogues; physicochemical properties of **45** and UR-MN212; computational chemistry; and references (PDF)
Molecular formula strings (CSV)
7yxa-model146-MB310-minMMFF94x (PDB)
7yxa-model146-MB312-minMMFF94x (PDB)
7yxa-model146-MB315-minMMFF94x (PDB)

## Supplementary Material

jm3c01707_si_002.csv

jm3c01707_si_003.pdb

jm3c01707_si_004.pdb

jm3c01707_si_005.pdb

## Abbreviations Used

DMAP: 4-dimethylamino pyridine
5-TAMRA: 5-carboxy-tetramethylrhodamine
MeCN: acetonitrile
AC: adenylate cyclase
β_1_AR: β_1_-adrenergic receptors
β_2_AR: β_2_-adrenergic receptors
BRET: bioluminescence resonance energy transfer
CBD: cannabidiol
CRE: cyclic AMP response element
DAD: diode array detector
DPI: diphenyleneiodonium chloride
DMEM: Dulbecco’s Modified Eagle’s Medium
EtOAc: ethyl acetate
FBS: fetal bovine serum
PEG-6: G-protein-coupled receptor, hexaethylene glycol
oGPCR: orphan G-protein-coupled receptor
HBSS: Hank’s balanced salt solution
LiAlH_4_: lithium aluminum hydride
MOE: molecular operating environment
M_1_R: muscarinic acetylcholine receptor M1
Nluc: nanoluciferase
NHS: *N*-hydroxysuccinimide
Pd/C: palladium-on-carbon
PE: petroleum ether
KHMDS: potassium bis(trimethylsilyl)amide
S1P: sphingosine-1-phosphate
S1P_5_: sphingosine-1-phosphate receptor 5
PEG-4: tetraethylene glycol
TM: transmembrane
AT1R: type-1 angiotensin II receptor
