# Supplementary material for: Development of Fluorescent AF64394 Analogues Enables Real-Time Binding Studies for the Orphan Class A GPCR GPR3
Source: J Med Chem. 2023 Oct 31;66(21):15025–41. doi: 10.1021/acs.jmedchem.3c01707 (PMC10641823; doi:10.1021/acs.jmedchem.3c01707)
Supplement: Supplementary file 1 — jm3c01707_si_001.pdf [file jm3c01707_si_001.pdf]

Supporting Information

Development of fluorescent AF64394 analogs enables real-time binding studies for the orphan class A GPCR GPR3

Merlin Bresinsky[a], Aida Shahraki[b], Peter Kolb[b], Steffen Pockes[a,c,*], and Hannes Schihada[b,*]

[a]Institute of Pharmacy, University of Regensburg, Universitätsstraße 31, 93053 Regensburg, Germany

[b]Department of Pharmaceutical Chemistry, University of Marburg, Marbacher Weg 8, 35032 Marburg, Germany

[c]Department of Medicinal Chemistry, Institute for Therapeutics Discovery and Development, University of Minnesota, Minneapolis, MN 55414, USA

Corresponding authors: steffen.pockes@ur.de **(Steffen Pockes)**, schihada@uni-marburg.de **(Hannes Schihada)**

Content

1. Preparation of the linker **3** and **6** S3
2. Preparation of the fluorescence ligands **46-55** S6
3. Chemical structures of **7-10**, **13**, **16**, **19**, **22**, **25**, **28**, **31**, **38**, and **45** S27
4. NMR spectra of compounds **38-44** and **45-51** S31
5. RP-HPLC purity control of compounds **45-55** S39
6. RP-HPLC stability control of compound **46** and **48** S50
7. Fluorescence properties S52
8. Activity of **45** in a CRE reporter gene assay with GPR3, GPR6 and GPR12 .... S53
9. Comparison of dissociation rates of *ortho*-labeled fluorescent AF64394 analogs S54
10. Physicochemical properties of **45** and UR-MN212 .... S55
11. Basal BRET values recorded by different instruments S56
12. Computational chemistry S57
13. References S61

1. Preparation of the linker 3 and 6

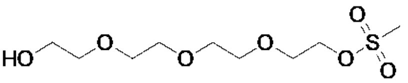

2-(2-(2-(2-Hydroxyethoxy)ethoxy)ethoxy)ethyl methanesulfonate (1)[1]

The reaction was carried out by solving tetraethylene glycol (PEG-4) (5.60 g, 28.83 mmol, 1 eq) and triethylamine (4.02 mL, 28.83 mmol, 1 eq) in 100 mL dichloromethane. The mixture was cooled to 0 °C by using an ice bath. Subsequently, methanesulfonyl chloride (2.23 mL, 28.83 mmol, 1 eq) in 300 mL dichloromethane was added dropwise over a period of 10 hours. After addition, the ice bath was removed, and stirring was continued overnight. Evaporation of the solvent and purification by column chromatography (DCM/Methanol 98/2-95/5) resulted in a yellowish oil (2.76 g, 35%). ^{1}H NMR (300 MHz, $CDCl_3$) δ 4.31 – 4.22 (m, 2H), 3.69 – 3.43 (m, 14H), 3.05 (bs, 1H), 2.97 (s, 3H). ^{13}C NMR (75 MHz, $CDCl_3$) δ 72.45, 70.45, 70.32, 70.17, 69.40, 68.87, 61.45, 37.49. HRMS (ESI-MS) *m/z*: [M+H$^+$] calculated for $C_9H_{21}O_7S^+$: 273.1003, found 273.1005.; $C_9H_{20}O_7S$ (272.31).

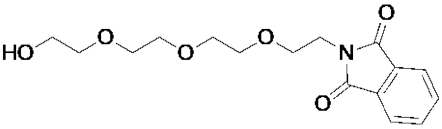

2-(2-(2-(2-Hydroxyethoxy)ethoxy)ethoxy)ethyl)isoindoline-1,3-dione (2)[2]

Potassium phthalimide (2.12 g, 11.46 mmol, 1.2 eq) and **1** (2.60 g, 9.55 mmol, 1 eq) were dissolved in 200 mL anhydrous DMF. The mixture was heated to 80 °C, and stirring was continued overnight. Subsequently, the solvent was removed under reduced pressure, and the crude product was purified by column chromatography (DCM/Methanol 95/5), yielding a colorless oil (2.05 g, 66%). ^{1}H NMR (300 MHz, $CDCl_3$) δ 7.86 – 7.79 (m, 2H), 7.73 – 7.66 (m, 2H), 3.88 (t, *J* = 5.8 Hz, 2H), 3.76 – 3.52 (m, 14H), 2.64 (s, 1H). ^{13}C NMR (75 MHz, $CDCl_3$) δ 168.32, 133.95, 132.14, 123.50, 72.49, 70.66, 70.53, 70.34, 70.10, 67.97, 61.74, 37.28. HRMS (ESI-MS) *m/z*: [M+H$^+$] calculated for $C_{16}H_{22}NO_6^+$: 324.1442, found 324.1446.; $C_{16}H_{21}NO_6$ (323.35).

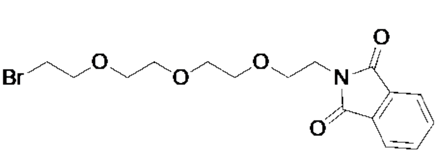

2-(2-(2-(2-Bromoethoxy)ethoxy)ethoxy)ethyl)isoindoline-1,3-dione (3)[3]

2 (2.05 g, 6.34 mmol, 1 eq) was dissolved in 200 mL dichloromethane and cooled by an ice bath to 0 °C. Subsequently, triphenylphosphine (2.00 g, 7.61 mmol, 1.2 eq) in 50 mL dichloromethane was quickly dropped into the solution. After 15 minutes, *N*-bromosuccinimide (1.35 g, 7.61 mmol, 1.2 eq) was added. The mixture was now stirred at 0 °C for 2 h. Reaction progress was monitored by TLC (R_f = 0.85, DCM/Methanol 95/5). Evaporation of the solvent and purification by column chromatography (DCM/Methanol 95/5) of the resuide resulted in a colorless oil (1.70 g, 69%). ^{1}H NMR

(300 MHz, $CDCl_3$) δ 7.82 – 7.73 (m, 2H), 7.70 – 7.63 (m, 2H), 3.84 (t, *J* = 5.8 Hz, 2H), 3.75 – 3.66 (m, 4H), 3.63 – 3.52 (m, 8H), 3.39 (t, *J* = 6.3 Hz, 2H). ^{13}C NMR (75 MHz, $CDCl_3$) δ 168.20, 133.95, 132.09, 123.20, 71.13, 70.59, 70.45, 70.09, 67.89, 37.25, 30.42. HRMS (ESI-MS) *m/z*: [M+H$^+$] calculated for $C_{16}H_{21}BrNO_5^+$: 386.0596, found 386.0598.; $C_{16}H_{20}BrNO_5$ (386.24).

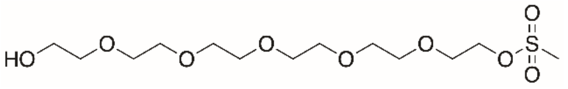

17-Hydroxy-3,6,9,12,15-pentaoxaheptadecyl methanesulfonate (4)[4]

The reaction was carried out by solving hexaethylene glycole (PEG-6) (5.60 g, 19.83 mmol, 1 eq) and triethylamine (2.77 mL, 19.83 mmol, 1 eq) in 100 mL dichloromethane. The mixture was cooled to 0 °C by using an ice bath. Subsequently, methanesulfonyl chloride (1.53 mL, 19.83 mmol, 1 eq) in 300 mL dichloromethane was added dropwise over a period of 10 hours. After addition, the ice bath was removed, and stirring was continued overnight. Evaporation of the solvent and purification by column chromatography (DCM/Methanol 98/2-95/5) resulted in a yellowish oil (3.14 g, 44%). ^1H NMR (300 MHz, $CDCl_3$) δ 4.41 – 4.35 (m, 2H), 3.80 – 3.56 (m, 22H), 3.08 (s, 3H), 2.69 (bs, 1H). ^{13}C NMR (75 MHz, $CDCl_3$) δ 72.54, 70.62, 70.58, 70.55, 70.53, 70.32, 69.39, 69.03, 61.74, 37.76. HRMS (ESI-MS) *m/z*: [M+H$^+$] calculated for $C_{13}H_{29}O_9S^+$: 361.1527, found 361.1531.; $C_{13}H_{28}O_9S$ (360.42).

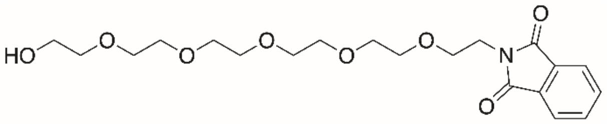

2-(17-Hydroxy-3,6,9,12,15-pentaoxaheptadecyl)isoindoline-1,3-dione (5)[5]

Potassium phthalimide (1.86 g, 10.02 mmol, 1.2 eq) and **4** (3.01 g, 8.35 mmol, 1 eq) were dissolved in 200 mL anhydrous DMF. The mixture was heated to 80 °C, and stirring was continued overnight. Subsequently, the solvent was removed under reduced pressure, and the crude product was purified by column chromatography (DCM/Methanol 95/5), yielding a colorless oil (3.44 g, 100%). ^1H NMR (300 MHz, $CDCl_3$) δ 7.87 – 7.78 (m, 2H), 7.75 – 7.65 (m, 2H), 3.88 (t, *J* = 5.7 Hz, 2H), 3.76 – 3.54 (m, 22H), 2.69 (s, 1H). ^{13}C NMR (75 MHz, $CDCl_3$) δ 168.29, 133.95, 132.15, 123.25, 72.54, 70.61, 70.55, 70.52, 70.35, 70.07, 67.92, 61.75, 37.26. HRMS (ESI-MS) *m/z*: [M+H$^+$] calculated for $C_{20}H_{30}NO_8^+$: 412.1966, found 412.1970.; $C_{20}H_{29}NO_8$ (411.45).

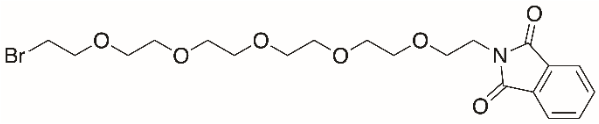

2-(17-Bromo-3,6,9,12,15-pentaoxaheptadecyl)isoindoline-1,3-dione (6)

5 (3.44 g, 8.36 mmol, 1 eq) was dissolved in 200 mL dichloromethane and cooled by an ice bath to 0 °C. Subsequently, triphenylphosphine (2.63 g, 10.03 mmol, 1.2 eq) in 50 mL dichloromethane was quickly dropped into the solution. After 15 minutes, *N*-bromosuccinimide (1.79 g, 10.03 mmol, 1.2 eq) was added. The mixture was now stirred at 0 °C for 2 h. Reaction progress was monitored by TLC (R_f = 0.85, DCM/Methanol 95/5). Evaporation of the solvent and purification by column chromatography (DCM/Methanol 95/5) of the resuide resulted in a colorless oil (2.55 g, 64%). ^{1}H NMR (300 MHz, $CDCl_3$) δ 7.84 – 7.78 (m, 2H), 7.73 – 7.66 (m, 2H), 3.87 (t, *J* = 0.7 Hz, 2H), 3.78 (t, *J* = 6.3 Hz, 2H), 3.71 (t, *J* = 6.0 Hz, 2H), 3.67 – 3.52 (m, 16H), 3.44 (t, *J* = 6.3 Hz, 2H). ^{13}C NMR (75 MHz, $CDCl_3$) δ 168.25, 133.95, 132.14, 123.24, 71.20, 70.64, 70.62, 70.59, 70.55, 70.53, 70.08, 67.91, 37.25, 30.39. HRMS (ESI-MS) *m/z*: [M+H$^+$] calculated for $C_{20}H_{29}BrNO_7^+$: 474.1122, found 474.1120.; $C_{20}H_{28}BrNO_7$ (474.35).

2. Preparation of the fluorescence ligands 46-55

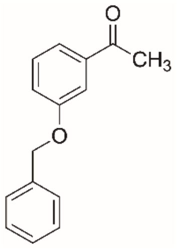

1-(3-(Benzyloxy)phenyl)ethan-1-one (11)[6]

The title compound was synthesized from 3-hydroxy acetophenone (4.42 g, 32.49 mmol, 1 eq), benzyl bromide (8.33 g, 5.79 mL, 48.74 mmol, 1.5 eq), and potassium carbonate (13.47 g, 97.48 mmol, 3 eq) in DMF according to the general procedure A (R_f = 0.66 in EtOAc/PE 1/4) yielding a colorless solid (7.35 g, 100%). ^{1}H NMR (300 MHz, $CDCl_3$) δ 7.62 – 7.55 (m, 2H), 7.49 – 7.36 (m, 6H), 7.22 – 7.17 (m, 1H), 5.12 (s, 2H), 2.59 (s, 3H). ^{13}C NMR (75 MHz, $CDCl_3$) δ 197.91, 159.00, 138.53, 136.56, 129.70, 128.70, 128.19, 127.62, 121.38, 120.31, 113.61, 70.19, 26.79. HRMS (EI-MS) *m/z*: [$M^{\bullet+}$] calculated for $C_{15}H_{14}O_2^{\bullet+}$: 226.09883, found 226.09908; $C_{15}H_{14}O_2$ (226.28).

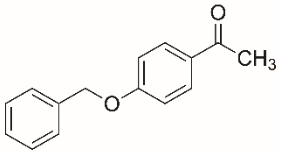

1-(4-(Benzyloxy)phenyl)ethan-1-one (12)[7]

The title compound was synthesized from 4-hydroxy acetophenone (4.42 g, 32.49 mmol, 1 eq), benzyl bromide (8.33 g, 5.79 mL, 48.74 mmol, 1.5 eq), and potassium carbonate (13.47 g, 97.48 mmol, 3 eq) in DMF according to the general procedure A (R_f = 0.66 in EtOAc/PE 1/4) yielding a colorless solid (7.35 g, 100%). ^{1}H NMR (300 MHz, $CDCl_3$) δ 7.97 – 7.90 (m, 2H), 7.46 – 7.29 (m, 5H), 7.05 – 6.95 (m, 2H), 5.10 (s, 2H), 2.53 (s, 3H). ^{13}C NMR (75 MHz, $CDCl_3$) δ 196.76, 162.64, 136.23, 130.66, 130.51, 128.74, 128.28, 127.53, 114.58, 70.13, 26.41. HRMS (EI-MS) *m/z*: [$M^{\bullet+}$] calculated for $C_{15}H_{14}O_2^{\bullet+}$: 226.09883, found 226.09829; $C_{15}H_{14}O_2$ (226.28).

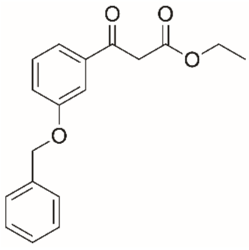

Ethyl 3-(3-(benzyloxy)phenyl)-3-oxopropanoate (14)[8]

The β-ketoester **14** was prepared from **11** (7.35 g, 32.48 mmol, 1 eq), sodium hydride (6.50 g, 162.41 mmol, 5 eq), and diethyl carbonate (19.19 g, 20.53 mL, 162.41 mmol, 5 eq) in DMF according to the general procedure B (R_f = 0.5, EtOAc/PE 1/5) yielding

a yellowish oil (4.35 g, 45%). ^{1}H NMR (300 MHz, $CDCl_3$) δ 12.63 (s, 0.2H (enol form)), 7.62 – 7.03 (m, 9H), 5.67 (s, 0.2H (enol form), 5.09 (s, 2H), 4.33 – 4.17 (m, 1.6H (keto-enol tautomerism)), 4.13 (q, *J* = 7.1 Hz, 0.4H (keto-enol tautomerism)), 3.97 (s, 1.6H (keto form)), 1.34 (t, *J* = 7.1 Hz, 0.6H), 1.26 (t, *J* = 7.1 Hz, 2.4H (keto-enol tautomerism)). ^{13}C NMR (75 MHz, $CDCl_3$) δ 192.35, 167.52, 159.09, 137.39, 136.41, 129.88, 128.70, 128.21, 127.60, 121.43, 121.02, 113.71, 87.71, 70.20, 70.10, 61.50, 60.42, 46.10, 14.36, 14.15. HRMS (ESI-MS) *m/z*: [M+H$^+$] calculated for $C_{18}H_{19}O_4^+$: 299.1278, found 299.1280; $C_{18}H_{18}O_4$(298.34).

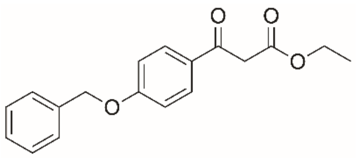

Ethyl 3-(4-(benzyloxy)phenyl)-3-oxopropanoate (15)[9]

The β-ketoester **15** was prepared from **12** (7.35 g, 32.48 mmol, 1 eq), sodium hydride (6.50 g, 162.41 mmol, 5 eq), and diethyl carbonate (19.19 g, 20.53 mL, 162.41 mmol, 5 eq) in DMF according to the general procedure B (R_f = 0.5, EtOAc/PE 1/5) yielding a yellowish solid (6.40 g, 66%). ^{1}H NMR (300 MHz, $CDCl_3$) δ 12.75 (s, 0.2H (enol form)), 7.90 – 7.65 (m, 2H), 7.40 – 7.25 (m, 5H), 6.99 – 6.90 (m, 2H), 5.57 (s, 0.2H (enol form)), 5.01 (s, 2H), 4.27 – 4.11 (m, 1.6H (keto-enol tautomerism)), 4.11 – 4.02 (m, 0.4H (keto-enol tautomerism)), 3.88 (s, 1.6H (keto form)), 1.27 (t, *J* = 7.1 Hz, 0.6H (keto-enol tautomerism)), 1.20 (t, *J* = 7.1 Hz, 2.4H (keto-enol tautomerism)). ^{13}C NMR (75 MHz, $CDCl_3$) δ 193.54, 168.08, 158.21, 135.94, 134.52, 131.11, 128.79, 128.37, 127.60, 126.98, 121.07, 112.98, 92.63, 70.73, 60.90, 50.52, 14.08. HRMS (ESI-MS) *m/z*: [M+H$^+$] calculated for $C_{18}H_{19}O_4^+$: 299.1278, found 299.1282; $C_{18}H_{18}O_4$(298.34).

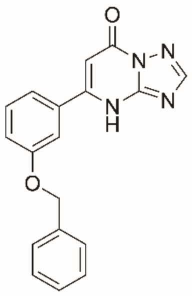

5-(3-(Benzyloxy)phenyl)-[1,2,4]triazolo[1,5-*a*]pyrimidin-7(4*H*)-one (17)

The title compound was synthesized from **14** (4.06 g, 13.61 mmol, 1 eq) and 3-amino-1,2,4-triazole (1.14 g, 13.61 mmol, 1eq) in acetic acid according to the general procedure C (R_f = 0.85 in DCM/Methanol 95/5) yielding **17** as a white solid (910 mg, 21%). ^{1}H NMR (300 MHz, DMSO-d_6) δ 13.60 (bs, 1H), 8.42 (s, 1H), 7.61 – 7.53 (m, 1H), 7.50 – 7.31 (m, 7H), 7.24 – 7.14 (m, 1H), 6.42 (s, 1H), 5.25 – 5.14 (m, 2H). ^{13}C NMR (75 MHz, DMSO-d_6) δ 206.47, 194.29, 159.04, 156.48, 151.23, 137.24, 130.55, 128.92, 128.37, 128.21, 128.18, 120.40, 118.27, 113.88, 98.10, 69.93. HRMS (ESI-

MS) *m/z*: [M+H+] calculated for $C_{18}H_{15}N_4O_2^+$: 319.1190, 319.1193; $C_{18}H_{14}N_4O_2$ (318.34).

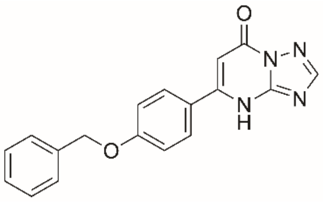

5-(4-(Benzyloxy)phenyl)-[1,2,4]triazolo[1,5-*a*]pyrimidin-7(4*H*)-one (18)

The title compound was synthesized from **15** (2.15 g, 7.19 mmol, 1 eq) and 3-amino-1,2,4-triazole (0.61 g, 7.19 mmol, 1eq) in acetic acid according to the general procedure C (R_f = 0.85 in DCM/Methanol 95/5) yielding **18** as a white solid (430 mg, 19%). ^{1}H NMR (300 MHz, DMSO-d_6) δ 13.47 (bs, 1H), 8.37 (s, 1H), 7.99 – 7.81 (m, 2H), 7.50 – 7.32 (m, 5H), 7.21 – 7.07 (m, 2H), 6.31 (s, 1H), 5.28 – 5.13 (m, 2H). ^{13}C NMR (75 MHz, DMSO-d_6) δ 206.48, 192.42, 161.08, 156.48, 137.08, 129.59, 128.96, 128.43, 128.27, 128.21, 118.15, 115.60, 96.74, 69.86. HRMS (ESI-MS) *m/z*: [M+H+] calculated for $C_{18}H_{15}N_4O_2^+$: 319.1190, found 319.1201; $C_{18}H_{14}N_4O_2$ (318.34).

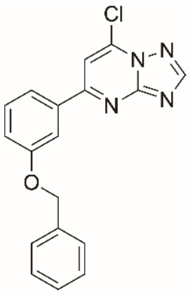

5-(3-(Benzyloxy)phenyl)-7-chloro-[1,2,4]triazolo[1,5-*a*]pyrimidine (20)

20 was prepared from **17** (900 mg, 2.83 mmol, 1 eq) in phosphoryl chloride according to the general procedure D (R_f = 0.50, DCM/Methanol 98/2) yielding a yellow solid (660 mg, 69%). ^{1}H NMR (300 MHz, $CDCl_3$) δ 8.55 (s, 1H), 7.88 (t, *J* = 2.1 Hz, 1H), 7.75 – 7.59 (m, 2H), 7.50 – 7.30 (m, 6H), 7.19 – 7.11 (m, 1H), 5.15 (s, 2H). ^{13}C NMR (75 MHz, $CDCl_3$) δ 161.50, 159.50, 156.57, 155.95, 139.64, 136.64, 136.49, 130.21, 128.66, 128.15, 127.56, 120.42, 119.10, 113.63, 108.03, 70.26. HRMS (ESI-MS) *m/z*: [M+H+] calculated for $C_{18}H_{14}ClN_4O^+$: 337.0851, found 337.0861; $C_{18}H_{13}ClN_4O$ (336.78).

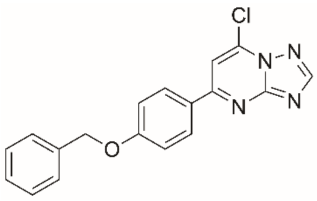

5-(4-(Benzyloxy)phenyl)-7-chloro-[1,2,4]triazolo[1,5-*a*]pyrimidine (21)

21 was prepared from **18** (700 mg, 2.20 mmol, 1 eq) in phosphoryl chloride according to the general procedure D (R_f = 0.50, DCM/Methanol 98/2) yielding a yellow solid (470 mg, 63%). ^{1}H NMR (300 MHz, $CDCl_3$) δ 8.50 (s, 1H), 8.19 – 8.06 (m, 1H), 7.65 – 6.99 (m, 9H), 5.14 (d, *J* = 9.1 Hz, 2H). ^{13}C NMR (75 MHz, $CDCl_3$) δ 162.01, 161.27, 156.35, 139.32, 136.17, 131.45, 129.69, 128.77, 128.71, 128.27, 127.52, 120.87, 115.43, 108.78, 107.30, 70.21. HRMS (ESI-MS) *m/z*: [M+H$^+$] calculated for $C_{18}H_{14}ClN_4O^+$: 337.0851, found 337.0852; $C_{18}H_{13}ClN_4O$ (336.78).

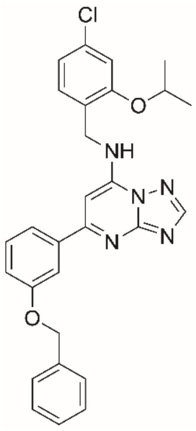

5-(3-(Benzyloxy)phenyl)-*N*-(4-chloro-2-isopropoxybenzyl)-[1,2,4]triazolo[1,5-*a*]pyrimidin-7-amine (23)

Synthesis of **23** was performed with **20** (470 mg, 1.40 mmol, 1 eq), (4-chloro-2-isopropoxyphenyl)methanamine (**9**) (836 mg, 4.19 mmol, 3 eq), and triethylamine (339 mg, 467 µL, 3.35 mmol, 2.4 eq) in dichloromethane according to the general procedure E (R_f = 0.45, EtOAc/PE 1/1), yielding a yellow oil (700 mg, 100%). ^{1}H NMR (300 MHz, $CDCl_3$) δ 8.33 – 8.31 (m, 1H), 7.84 – 7.79 (m, 1H), 7.69 – 7.60 (m, 1H), 7.51 – 7.05 (m, 9H), 6.97 – 6.87 (m, 2H), 6.63 (d, *J* = 14.7 Hz, 1H), 5.15 (s, 2H), 4.75 – 4.54 (m, 3H), 1.37 (d, *J* = 0.6 Hz, 6H). ^{13}C NMR (101 MHz, $CDCl_3$) δ 161.90, 159.20, 156.36, 155.75, 155.00, 147.49, 139.42, 136.82, 135.11, 130.04, 129.70, 129.48, 128.61, 128.04, 127.59, 124.53, 123.17, 120.48, 120.19, 117.41, 113.41, 85.27, 70.21, 42.08, 21.99. HRMS (ESI-MS) *m/z*: [M+H$^+$] calculated for $C_{28}H_{27}ClN_5O_2^+$: 500.1848, found 500.1860; $C_{28}H_{26}ClN_5O_2$ (500.00).

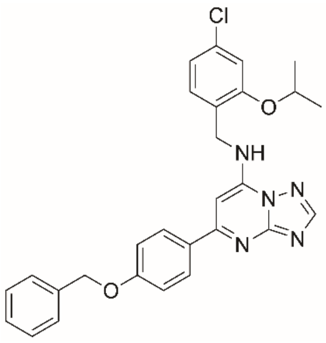

5-(4-(Benzyloxy)phenyl)-*N*-(4-chloro-2-isopropoxybenzyl)-[1,2,4]triazolo[1,5-*a*]pyrimidin-7-amine (24)

Synthesis of **24** was performed with **21** (470 mg, 1.40 mmol, 1 eq), (4-chloro-2-isopropoxyphenyl)methanamine (**9**) (836 mg, 4.19 mmol, 3 eq), and triethylamine (339 mg, 467 µL, 3.35 mmol, 2.4 eq) in dichloromethane according to the general procedure E (R_f = 0.30, EtOAc/PE 1/1), yielding a yellow oil (450 mg, 64%). ^{1}H NMR (300 MHz, $CDCl_3$) δ 8.28 (s, 1H), 8.05 – 7.97 (m, 2H), 7.42 – 6.81 (m, 11H), 6.53 (d, *J* = 17.3 Hz, 1H), 5.06 (s, 2H), 4.68 – 4.49 (m, 3H), 1.34 (d, *J* = 3.3 Hz, 6H).
^{13}C NMR (75 MHz, $CDCl_3$) δ 161.57, 160.80, 156.26, 155.80, 154.75, 147.49, 136.58, 134.78, 130.46, 129.96, 129.36, 129.19, 128.64, 128.10, 127.50, 124.82, 123.57, 120.48, 114.85, 113.38, 112.84, 84.54, 70.03, 41.70, 22.01. HRMS (ESI-MS) *m/z*: [M+H$^+$] calculated for $C_{28}H_{27}ClN_5O_2^+$: 500.1848, found 500.1850; $C_{28}H_{26}ClN_5O_2$ (500.00).

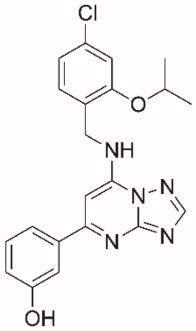

3-(7-((4-Chloro-2-isopropoxybenzyl)amino)-[1,2,4]triazolo[1,5-*a*]pyrimidin-5-yl)phenol (26)

Compound **26** was obtained by catalyzed hydrogenation of **23** (670 mg, 1.340 mmol, 1eq) in methanol according to the general procedure F (R_f = 0.25, EtOAc/PE 2/1), yielding a yellow solid (480 mg, 87%). ^{1}H NMR (300 MHz, $CDCl_3$) δ 8.37 (s, 1H), 8.02 (s, 1H), 7.76 (s, 1H), 7.38 – 7.26 (m, 2H), 7.21 – 7.07 (m, 2H), 6.99 – 6.88 (m, 2H), 6.79 – 6.59 (m, 1H), 4.72 – 4.53 (m, 3H), 1.35 (d, *J* = 6.0 Hz, 6H). ^{13}C NMR (101 MHz, $CDCl_3$) δ 162.79, 157.53, 156.19, 155.64, 151.99, 147.52, 137.45, 134.78, 129.99, 129.59, 129.39, 124.37, 123.17, 120.54, 118.68, 115.05, 112.76, 87.69, 70.37, 22.10. HRMS (ESI-MS) *m/z*: [M+H$^+$] calculated for $C_{21}H_{21}ClN_5O_2^+$: 410.1378, found 410.1389.; $C_{21}H_{20}ClN_5O_2$ (409.87).

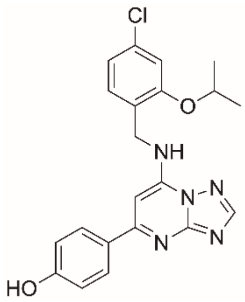

4-(7-((4-Chloro-2-isopropoxybenzyl)amino)-[1,2,4]triazolo[1,5-*a*]pyrimidin-5-yl)phenol (27)

Compound **27** was obtained by catalyzed hydrogenation of **24** (450 mg, 0.900 mmol, 1 eq) in methanol according to the general procedure F (R_f = 0.25, EtOAc/PE 2/1), yielding a yellow solid (230 mg, 62%). ^{1}H NMR (300 MHz, DMSO-*d*$_6$) δ 8.64 – 8.51 (m, 1H), 8.46 (s, 1H), 8.02 – 7.94 (m, 1H), 7.36 – 6.67 (m, 7H), 4.88 – 4.47 (m, 3H), 1.34 – 1.22 (m, 6H). ^{13}C NMR (75 MHz, DMSO-*d*$_6$) δ 160.27, 156.33, 155.81, 155.52, 154.77, 148.23, 133.26, 130.25, 129.44, 129.07, 128.61, 126.67, 125.85, 120.81, 120.65, 115.86, 113.95, 84.51, 71.06, 22.33. HRMS (ESI-MS) *m/z*: [M+H$^+$] calculated for $C_{21}H_{21}ClN_5O_2^+$: 410.1378, found 410.1385.; $C_{21}H_{20}ClN_5O_2$ (409.87).

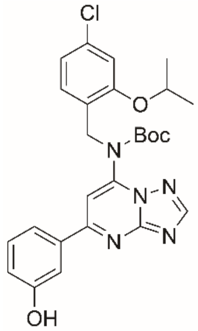

***tert*-Butyl (4-chloro-2-isopropoxybenzyl)(5-(3-hydroxyphenyl)-[1,2,4]triazolo[1,5-*a*]pyrimidin-7-yl)carbamate (29)**

The respective N-Boc protected aromatic amine was obtained from **26** (480 mg, 1.171 mmol, 1 eq), DMAP (cat.), triethylamine (130 mg, 181 µL, 1.1 eq), and di-*tert*-butyldicarbonate (281 mg, 1.288 mmol, 1.1 eq) in a total of 50 mL chloroform according to the general procedure G (R_f = 0.78, EtOAc/PE 2/1), yielding a colorless oil (460 mg, 77%). ^{1}H NMR (300 MHz, $CDCl_3$) δ 8.32 – 8.29 (m, 1H), 7.99 – 7.87 (m, 2H), 7.46 (t, *J* = 0.8 Hz, 1H), 7.33 – 7.23 (m, 2H), 6.96 – 6.87 (m, 2H), 6.60 (d, *J* = 16.6 Hz, 1H), 4.71 – 4.55 (m, 3H), 1.57 (s, 9H), 1.36 (d, *J* = 0.7 Hz, 6H). ^{13}C NMR (75 MHz, $CDCl_3$) δ 160.90, 156.31, 155.80, 155.72, 155.00, 151.44, 147.68, 139.55, 130.03, 129.73, 129.62, 129.46, 124.93, 124.46, 123.31, 120.70, 120.44, 112.79, 85.26, 83.77, 42.67, 27.75, 22.14. HRMS (ESI-MS) *m/z*: [M+H$^+$] calculated for $C_{26}H_{29}ClN_5O_4^+$: 510.1903, found 510.1910.; $C_{26}H_{28}ClN_5O_4$ (509.99).

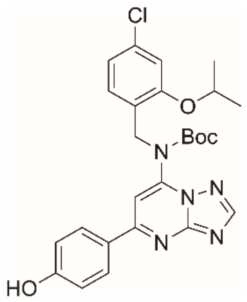

***tert*-Butyl (4-chloro-2-isopropoxybenzyl)(5-(4-hydroxyphenyl)-[1,2,4]triazolo[1,5-*a*]pyrimidin-7-yl)carbamate (30)**

The respective N-Boc protected aromatic amine was obtained from **27** (200 mg, 0.488 mmol, 1 eq), DMAP (cat.), triethylamine (54 mg, 75 µL, 0.537 mmol, 1.1 eq), and di-*tert*-butyldicarbonate (117 mg, 0.537 mmol, 1.1 eq) in a total of 50 mL chloroform according to the general procedure G (R_f = 0.78, EtOAc/PE 2/1), yielding a colorless oil (200 mg, 80%). ^{1}H NMR (300 MHz, $CDCl_3$) δ 8.31 (s, 1H), 8.11 – 8.04 (m, 2H), 7.32 – 7.18 (m, 3H), 6.95 – 6.88 (m, 2H), 6.58 (d, *J* = 16.6 Hz, 1H), 4.72 – 4.53 (m, 3H), 1.56 (s, 9H), 1.35 (d, *J* = 6.0 Hz, 6H). ^{13}C NMR (75 MHz, $CDCl_3$) δ 161.19, 156.33, 155.70, 154.94, 152.88, 151.47, 147.57, 135.33, 130.01, 129.73, 129.45, 128.88, 124.50, 123.16, 121.50, 120.50, 113.44, 85.08, 83.93, 42.04, 27.72, 22.00. HRMS (ESI-MS) *m/z*: [M+H$^+$] calculated for $C_{26}H_{29}ClN_5O_4^+$: 510.1903, found 510.1906.; $C_{26}H_{28}ClN_5O_4$ (509.99).

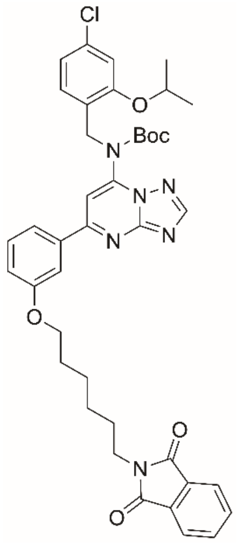

***tert*-Butyl (4-chloro-2-isopropoxybenzyl)(5-(3-((6-(1,3-dioxoisoindolin-2-yl)hexyl)oxy)phenyl)-[1,2,4]triazolo[1,5-*a*]pyrimidin-7-yl)carbamate (32)**

The synthesis of **32** was carried out with **29** (90 mg, 0.177 mmol, 1 eq), **7** (274 mg, 0.882 mmol, 5 eq), and potassium carbonate (122 mg, 0.882 mmol, 5 eq) in 10 mL DMF according to the general procedure H (method A, R_f = 0.20, EtOAc/PE 1/1), yielding a colorless oil (118 mg, 91%). ^{1}H NMR (300 MHz, $CDCl_3$) δ 8.35 (s, 1H), 8.00 – 7.91 (m, 2H), 7.88 – 7.78 (m, 2H), 7.76 – 7.65 (m, 2H), 7.49 (t, *J* = 7.9 Hz, 1H), 7.35 – 7.27 (m, 2H), 6.97 – 6.90 (m, 2H), 6.65 (d, *J* = 15.7 Hz, 1H), 4.73 – 4.57 (m, 3H), 3.88 – 3.38 (m, 4H), 2.22 – 1.63 (m, 8H), 1.58 (s, 9H), 1.38 (d, *J* = 6.1 Hz, 6H). ^{13}C NMR (75 MHz, $CDCl_3$) δ 168.08, 160.03, 157.36, 154.51, 152.90, 151.89, 149.51,

137.50, 133.90, 131.94, 131.62, 129.83, 129.68, 129.61, 129.55, 123.20, 122.67, 120.47, 118.14, 112.78, 93.89, 83.85, 70.25, 51.40, 48.07, 35.10, 28.41, 27.76, 25.53, 25.30, 22.15. HRMS (ESI-MS) *m/z*: [M+H$^+$] calculated for $C_{40}H_{44}ClN_6O_6^+$: 739.3005, found 739.3012.; $C_{40}H_{43}ClN_6O_6$ (739.27).

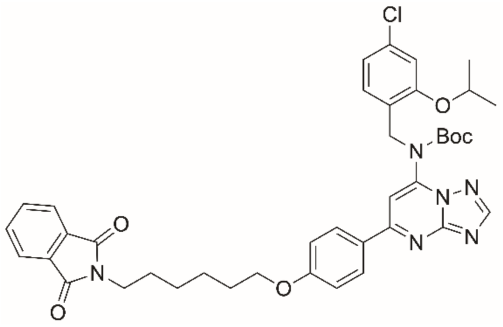

***tert*-Butyl (4-chloro-2-isopropoxybenzyl)(5-(4-((6-(1,3-dioxoisoindolin-2-yl)hexyl)oxy)phenyl)-[1,2,4]triazolo[1,5-*a*]pyrimidin-7-yl)carbamate (33)**

The synthesis of **33** was carried out with **30** (80 mg, 0.159 mmol, 1 eq), **7** (246 mg, 0.794 mmol, 5 eq), and potassium carbonate (110 mg, 0.794 mmol, 5 eq) in 10 mL DMF according to the general procedure H (method A, R_f = 0.20, EtOAc/PE 1/1), yielding a colorless oil (60 mg, 51%). HRMS (ESI-MS) *m/z*: [M+H$^+$] calculated for $C_{40}H_{44}ClN_6O_6^+$: 739.3005, found 739.3009; $C_{40}H_{43}ClN_6O_6$ (739.27).

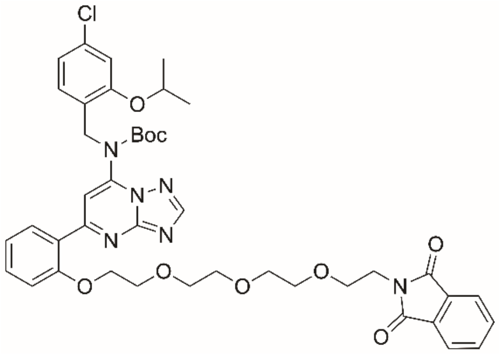

***tert*-Butyl (4-chloro-2-isopropoxybenzyl)(5-(2-(2-(2-(2-(2-(1,3-dioxoisoindolin-2-yl)ethoxy)ethoxy)ethoxy)ethoxy)phenyl)-[1,2,4]triazolo[1,5-*a*]pyrimidin-7-yl)carbamate (34)**

The synthesis of **34** was carried out with **28** (90 mg, 0.176 mmol, 1 eq), **3** (341 mg, 0.882 mmol, 5 eq), and potassium carbonate (122 mg, 0.882 mmol, 5 eq) in 10 mL DMF according to the general procedure H (method B, R_f = 0.25, EtOAc/PE 2/1), yielding a colorless oil (39 mg, 27%). ^{1}H NMR (400 MHz, $CDCl_3$) δ 8.31 (s, 1H), 7.86 – 7.82 (m, 1H), 7.82 – 7.78 (m, 2H), 7.69 – 7.65 (m, 2H), 7.46 – 7.40 (m, 1H), 7.34 – 7.28 (m, 1H), 7.23 – 7.09 (m, 2H), 6.87 – 6.81 (m, 2H), 6.63 (d, *J* = 4.1 Hz, 1H), 5.15 (d, *J* = 11.8 Hz, 2H), 4.56 – 4.43 (m, 1H), 4.11 – 4.03 (m, 2H), 3.85 (t, *J* = 5.9 Hz, 2H), 3.68 (t, *J* = 5.9 Hz, 2H), 3.60 – 3.46 (m, 10H), 1.41 (s, 9H), 1.16 (d, *J* = 1.5 Hz, 6H). ^{13}C NMR (75 MHz, $CDCl_3$) δ 168.22, 159.51, 156.34, 155.79, 154.09, 151.27, 148.73, 133.94, 132.09, 131.54, 130.95, 130.83, 129.71, 128.95, 126.27, 124.46, 123.21, 123.09, 120.26, 113.15, 94.10, 83.73, 70.57, 70.03, 69.81, 67.89, 37.21, 29.72, 27.55,

21.89. HRMS (ESI-MS) *m/z*: [M+H$^+$] calculated for $C_{42}H_{48}ClN_6O_9^+$: 815.3166, found 815.3173.; $C_{42}H_{47}ClN_6O_9$ (815.32).

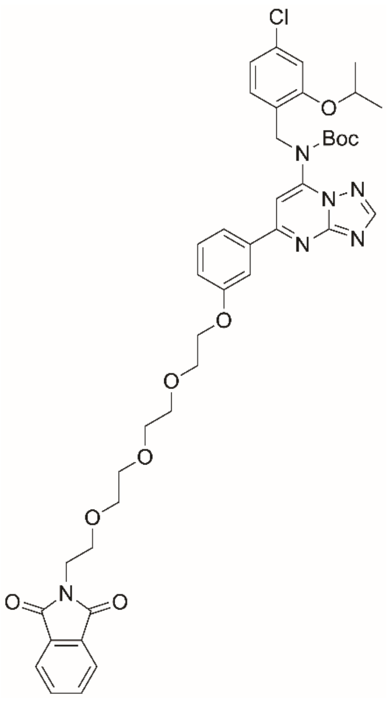

tert-Butyl (4-chloro-2-isopropoxybenzyl)(5-(3-(2-(2-(2-(2-(1,3-dioxoisoindolin-2-yl)ethoxy)ethoxy)ethoxy)ethoxy)phenyl)-[1,2,4]triazolo[1,5-*a*]pyrimidin-7-yl)carbamate (35)

The synthesis of **35** was carried out with **29** (82 mg, 0.161 mmol, 1 eq), **3** (311 mg, 0.804 mmol, 5 eq), and potassium carbonate (111 mg, 0.804 mmol, 5 eq) in 10 mL DMF according to the general procedure H (method B, R_f = 0.25, EtOAc/PE 2/1), yielding a colorless oil (49 mg, 37%). ^{1}H NMR (400 MHz, $CDCl_3$) δ 8.35 – 8.30 (m, 1H), 7.89 – 7.84 (m, 2H), 7.82 – 7.77 (m, 2H), 7.70 – 7.65 (m, 2H), 7.47 – 7.39 (m, 1H), 7.25 – 7.19 (m, 2H), 6.90 – 6.83 (m, 2H), 6.68 (d, *J* = 8.2 Hz, 1H), 5.11 (d, *J* = 10.3 Hz, 2H), 4.61 – 4.48 (m, 1H), 4.27 – 4.06 (m, 2H), 3.87 – 3.78 (m, 4H), 3.67 (t, *J* = 5.9 Hz, 2H), 3.58 – 3.47 (m, 8H), 1.56 (s, 9H), 1.21 (d, *J* = 6.0 Hz, 6H). ^{13}C NMR (101 MHz, $CDCl_3$) δ 168.21, 160.24, 156.25, 155.67, 151.70, 151.45, 150.45, 139.16, 133.91, 132.10, 129.53, 129.12, 128.78, 124.87, 124.41, 123.40, 123.19, 120.70, 120.48, 112.85, 91.43, 83.69, 70.66, 70.61, 70.56, 70.26, 70.05, 67.90, 40.87, 37.24, 29.70, 27.73, 21.99. HRMS (ESI-MS) *m/z*: [M+H$^+$] calculated for $C_{42}H_{48}ClN_6O_9^+$: 815.3166, found 815.3172.; $C_{42}H_{47}ClN_6O_9$ (815.32).

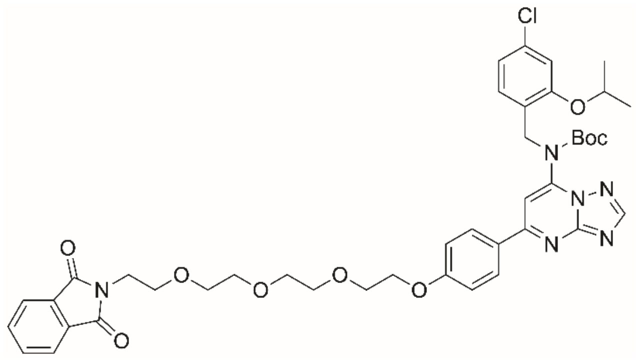

***tert*-Butyl (4-chloro-2-isopropoxybenzyl)(5-(4-(2-(2-(2-(2-(1,3-dioxoisoindolin-2-yl)ethoxy)ethoxy)ethoxy)ethoxy)phenyl)-[1,2,4]triazolo[1,5-*a*]pyrimidin-7-yl)carbamate (36)**

The synthesis of **36** was carried out with **30** (80 mg, 0.157 mmol, 1 eq), **3** (303 mg, 0.784 mmol, 5 eq), and potassium carbonate (108 mg, 0.784 mmol, 5 eq) in 10 mL DMF according to the general procedure H (method B, R_f = 0.25, EtOAc/PE 2/1), yielding a colorless oil (52 mg, 41%). ^{1}H NMR (300 MHz, $CDCl_3$) δ 8.30 (s, 1H), 8.08 – 8.00 (m, 2H), 7.81 – 7.76 (m, 2H), 7.69 – 7.64 (m, 2H), 7.27 – 7.18 (m, 2H), 6.91 – 6.81 (m, 2H), 6.63 (d, *J* = 3.3 Hz, 1H), 5.10 (d, *J* = 6.4 Hz, 2H), 4.61 – 4.44 (m, 1H), 4.25 – 4.12 (m, 2H), 3.87 – 3.78 (m, 4H), 3.67 (t, *J* = 5.8 Hz, 2H), 3.59 – 3.44 (m, 8H), 1.55 (s, 9H), 1.20 (d, *J* = 6.0 Hz, 6H). ^{13}C NMR (75 MHz, $CDCl_3$) δ 168.23, 160.40, 156.22, 155.64, 152.84, 151.43, 150.34, 134.41, 133.93, 132.08, 129.57, 128.83, 125.62, 125.06, 124.41, 123.20, 121.44, 120.47, 118.67, 113.35, 91.01, 83.88, 70.67, 70.61, 70.56, 70.05, 67.91, 50.90, 40.89, 37.22, 29.72, 27.70, 23.89. HRMS (ESI-MS) *m/z*: [M+H$^+$] calculated for $C_{42}H_{48}ClN_6O_9^+$: 815.3166, found 815.3187.; $C_{42}H_{47}ClN_6O_9$ (815.32).

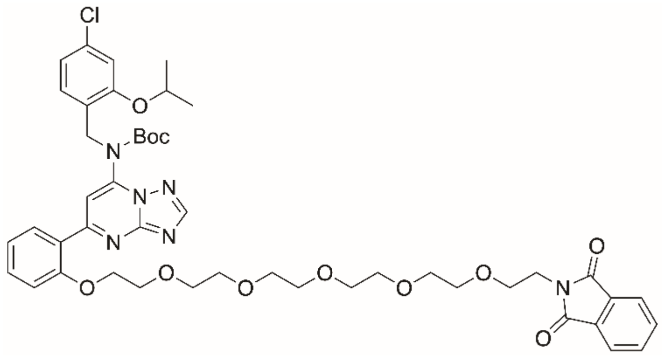

***tert*-Butyl (4-chloro-2-isopropoxybenzyl)(5-(2-((17-(1,3-dioxoisoindolin-2-yl)-3,6,9,12,15-pentaoxaheptadecyl)oxy)phenyl)-[1,2,4]triazolo[1,5-*a*]pyrimidin-7-yl)carbamate (37)**

The synthesis of **37** was carried out with **30** (200 mg, 0.392 mmol, 1 eq), **6** (930 mg, 1.961 mmol, 5 eq), and potassium carbonate (271 mg, 1.961 mmol, 5 eq) in 10 mL DMF according to the general procedure H (method B, R_f = 0.25, EtOAc/PE 2/1), yielding a colorless oil (300 mg, 85%). ^{1}H NMR (300 MHz, $CDCl_3$) δ 8.37 (s, 1H), 7.91 – 7.86 (m, 1H), 7.85 – 7.81 (m, 2H), 7.73 – 7.69 (m, 2H), 7.50 – 7.43 (m, 1H), 7.38 – 7.31 (m, 1H), 7.26 – 7.22 (m, 1H), 7.12 (d, *J* = 8.6 Hz, 1H), 6.87 – 6.83 (m, 2H), 6.70

(s, 1H), 5.15 (s, 2H), 4.57 – 4.43 (m, 1H), 4.09 (t, *J* = 5.3 Hz, 2H), 3.89 (t, *J* = 5.9 Hz, 2H), 3.82 (t, *J* = 5.4 Hz, 2H), 3.72 (t, *J* = 5.8 Hz, 2H), 3.66 – 3.50 (m, 16H), 1.42 (s, 9H), 1.17 (d, *J* = 6.0 Hz, 6H). ^{13}C NMR (75 MHz, $CDCl_3$) δ 168.15, 159.29, 157.76, 155.76, 154.13, 151.19, 150.19, 133.93, 132.04, 131.69, 130.89, 130.67, 129.74, 128.93, 126.23, 124.48, 123.15, 123.06, 120.22, 112.57, 93.92, 83.61, 70.63, 70.48, 70.00, 69.74, 67.83, 51.08, 50.38, 37.18, 27.52, 21.85. HRMS (ESI-MS) *m/z*: [M+H$^+$] calculated for $C_{46}H_{56}ClN_6O_{11}^+$: 903.3690, found 903.3704.; $C_{46}H_{55}ClN_6O_{11}$ (903.43).

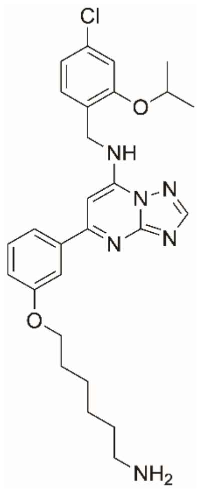

5-(3-((6-Aminohexyl)oxy)phenyl)-*N*-(4-chloro-2-isopropoxybenzyl)-[1,2,4]triazolo[1,5-*a*]pyrimidin-7-amine (39)

The title compound was synthesized from **32** (118 mg, 0.160 mmol, 1 eq) and hydrazine monohydrate (39.85 mg, 39 µL, 0.798 mmol, 5 eq) in 4 mL 1-butanol according to the general procedure I (R_f = 0.15 in DCM/Methanol/NH_3 conc. 50/50/1) yielding a colorless oil (11 mg, 9%). ^1H NMR (300 MHz, CD_3OD) δ 8.34 (s, 1H), 7.49 – 7.39 (m, 2H), 7.32 – 7.23 (m, 2H), 7.01 – 6.82 (m, 3H), 6.68 (s, 1H), 5.26 (s, 2H), 4.61 – 4.45 (m, 1H), 3.79 (t, *J* = 7.6 Hz, 2H), 2.66 (t, *J* = 7.1 Hz, 2H), 1.82 – 1.68 (m, 2H), 1.52 – 1.44 (m, 2H), 1.43 – 1.30 (m, 4H), 1.05 – 0.99 (m, 6H). ^{13}C NMR (75 MHz, $CDCl_3$) δ 160.53, 159.96, 156.58, 155.81, 154.31, 152.71, 133.66, 132.98, 131.03, 130.52, 128.84, 124.35, 123.77, 122.28, 120.58, 120.18, 118.14, 95.61, 73.16, 54.47, 36.31, 31.08, 30.21, 26.73, 24.54, 21.40. HRMS (ESI-MS) *m/z*: [M+H$^+$] calculated for $C_{27}H_{34}ClN_6O_2^+$: 509.2426, found 509.2428.; $C_{27}H_{33}ClN_6O_2$ (509.05).

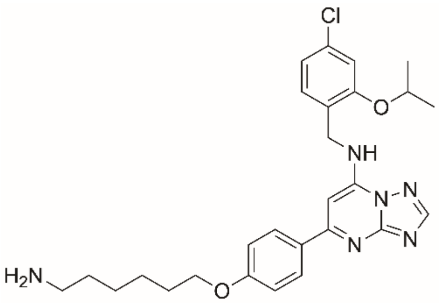

5-(4-((6-Aminohexyl)oxy)phenyl)-*N*-(4-chloro-2-isopropoxybenzyl)-[1,2,4]triazolo[1,5-*a*]pyrimidin-7-amine (40)

The title compound was synthesized from **33** (36 mg, 0.049 mmol, 1 eq) and hydrazine monohydrate (12.26 mg, 12 µL, 0.245 mmol, 5 eq) in 4 mL 1-butanol according to the general procedure I (R_f = 0.15 in DCM/Methanol/NH_3 conc. 50/50/1) yielding a colorless oil (3.9 mg, 16%). ^{1}H NMR (300 MHz, CD_3OD) δ 8.32 (s, 1H), 7.96 – 7.89 (m, 2H), 7.28 (d, *J* = 8.2 Hz, 1H), 6.99 (d, *J* = 2.1 Hz, 1H), 6.93 – 6.85 (m, 3H), 6.69 (s, 1H), 5.23 (s, 2H), 4.57 – 4.49 (m, 1H), 3.83 (t, *J* = 7.4 Hz, 2H), 2.86 (t, *J* = 7.3 Hz, 2H), 1.86 – 1.72 (m, 2H), 1.68 – 1.56 (m, 2H), 1.41 (q, *J* = 4.1 Hz, 4H), 1.02 (d, *J* = 6.0 Hz, 6H). HRMS (ESI-MS) *m/z*: [M+H$^+$] calculated for $C_{27}H_{34}ClN_6O_2^+$: 509.2426, found 509.2432.; $C_{27}H_{33}ClN_6O_2$ (509.05).

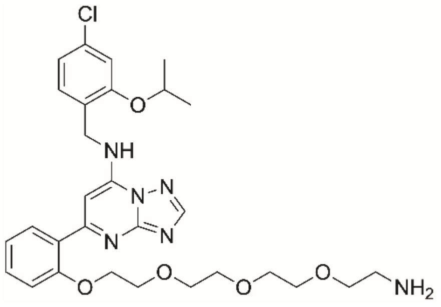

5-(2-(2-(2-(2-(2-Aminoethoxy)ethoxy)ethoxy)ethoxy)phenyl)-*N*-(4-chloro-2-isopropoxybenzyl)-[1,2,4]triazolo[1,5-*a*]pyrimidin-7-amine (41)

The title compound was synthesized from **34** (39.00 mg, 0.048 mmol, 1 eq) and hydrazine monohydrate (11.97 mg, 12 µL, 0.239 mmol, 5 eq) in 4 mL 1-butanol according to the general procedure I (R_f = 0.35 in DCM/Methanol/NH_3 conc. 50/50/1) yielding a colorless oil (11.7 mg, 36%). ^{1}H NMR (300 MHz, CD_3OD) δ 8.36 (s, 1H), 7.87 (d, *J* = 7.9 Hz, 1H), 7.40 – 7.31 (m, 2H), 7.07 (s, 1H), 7.02 (d, *J* = 1.9 Hz, 1H), 6.98 – 6.89 (m, 3H), 5.28 (s, 2H), 4.67 – 4.55 (m, 1H), 4.24 (t, *J* = 5.1 Hz, 2H), 3.80 (t, *J* = 5.1 Hz, 2H), 3.58 – 3.53 (m, 2H), 3.51 – 3.43 (m, 8H), 2.83 (t, *J* = 5.2 Hz, 2H), 1.12 (d, *J* = 6.0 Hz, 6H). ^{13}C NMR (75 MHz, CD_3OD) δ 159.92, 156.90, 156.45, 155.39, 152.60, 151.03, 133.28, 132.69, 127.64, 124.67, 120.08, 118.97, 118.08, 112.72, 90.26, 70.29, 70.20, 70.09, 69.56, 69.20, 51.22, 49.44, 39.98, 20.73. HRMS (ESI-MS) *m/z*: [M+H$^+$] calculated for $C_{29}H_{38}ClN_6O_5^+$: 585.2587, found 585.2584.; $C_{29}H_{37}ClN_6O_5$ (585.10).

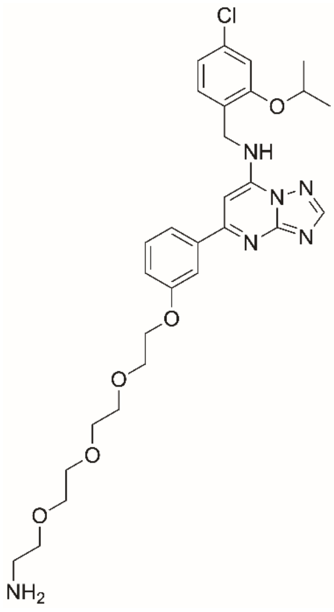

5-(3-(2-(2-(2-(2-Aminoethoxy)ethoxy)ethoxy)ethoxy)phenyl)-*N*-(4-chloro-2-isopropoxybenzyl)-[1,2,4]triazolo[1,5-*a*]pyrimidin-7-amine (42)

The title compound was synthesized from **35** (49.10 mg, 0.060 mmol, 1 eq) and hydrazine monohydrate (15.07 mg, 15 µL, 0.301 mmol, 5 eq) in 4 mL 1-butanol according to the general procedure **I** (R_f = 0.35 in DCM/Methanol/NH_3 conc. 50/50/1) yielding a colorless oil (2.9 mg, 8%). ^{1}H NMR (300 MHz, CD_3OD) δ 8.35 (s, 1H), 7.52 – 7.43 (m, 2H), 7.35 – 7.27 (m, 2H), 7.01 (d, *J* = 2.0 Hz, 1H), 6.95 – 6.88 (m, 3H), 5.24 (s, 2H), 4.67 – 4.51 (m, 1H), 4.22 (t, *J* = 5.1 Hz, 2H), 3.79 (t, *J* = 5.0 Hz, 2H), 3.57 – 3.51 (m, 2H), 3.50 – 3.41 (m, 8H), 2.87 (t, *J* = 5.0 Hz, 2H), 1.09 (d, *J* = 6.0 Hz, 6H). HRMS (ESI-MS) *m/z*: [M+H$^+$] calculated for $C_{29}H_{38}ClN_6O_5^+$: 585.2587, found 585.2595.; $C_{29}H_{37}ClN_6O_5$ (585.10).

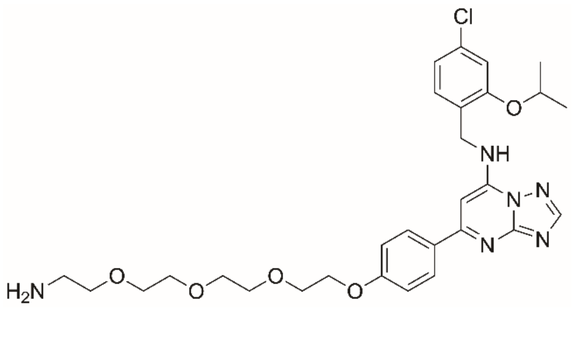

5-(4-(2-(2-(2-(2-Aminoethoxy)ethoxy)ethoxy)ethoxy)phenyl)-*N*-(4-chloro-2-isopropoxybenzyl)-[1,2,4]triazolo[1,5-*a*]pyrimidin-7-amine (43)

The title compound was synthesized from **36** (51.00 mg, 0.063 mmol, 1 eq) and hydrazine monohydrate (15.66 mg, 15 µL, 0.313 mmol, 5 eq) in 4 mL 1-butanol according to the general procedure **I** (R_f = 0.35 in DCM/Methanol/NH_3 conc. 50/50/1) yielding a colorless oil (8.6 mg, 20%). ^{1}H NMR (400 MHz, CD_3OD) δ 8.28 (s, 1H), 7.96 – 7.90 (m, 2H), 7.30 (d, *J* = 8.1 Hz, 1H), 6.99 (d, *J* = 1.9 Hz, 1H), 6.92 – 6.82 (m, 4H), 5.22 (s, 2H), 4.63 – 4.52 (m, 1H), 4.16 (t, *J* = 5.1 Hz, 2H), 3.77 (t, *J* = 5.1 Hz, 2H), 3.57 – 3.51 (m, 2H), 3.50 – 3.38 (m, 8H), 2.74 (t, *J* = 5.3 Hz, 2H), 1.09 (d, *J* = 6.0 Hz, 6H).

^{13}C NMR (101 MHz, CD_3OD) δ 161.62, 161.16, 157.56, 156.56, 152.68, 150.78, 134.23, 130.51, 128.97, 127.84, 124.10, 119.93, 115.54, 113.02, 91.13, 71.03, 70.33, 70.24, 70.14, 69.81, 69.18, 50.54, 50.33, 40.39, 20.54. HRMS (ESI-MS) *m/z*: [M+H$^+$] calculated for $C_{29}H_{38}ClN_6O_5^+$: 585.2587, found 585.2591.; $C_{29}H_{37}ClN_6O_5$ (585.10).

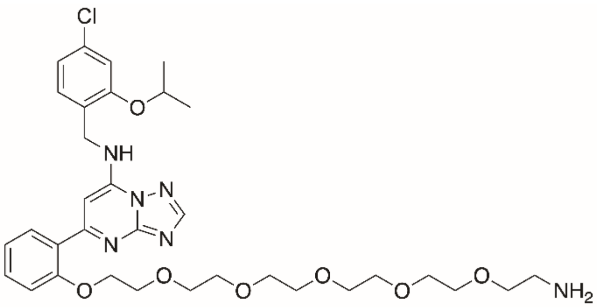

5-(2-((17-Amino-3,6,9,12,15-pentaoxaheptadecyl)oxy)phenyl)-*N*-(2-isopropoxybenzyl)-[1,2,4]triazolo[1,5-*a*]pyrimidin-7-amine (44)

The title compound was synthesized from **37** (300.00 mg, 0.332 mmol, 1 eq) and hydrazine monohydrate (83.12 mg, 81 µL, 1.660 mmol, 5 eq) in 10 mL 1-butanol according to the general procedure **I** (R_f = 0.35 in DCM/Methanol/NH_3 conc. 50/50/1) yielding a colorless oil (37.0 mg, 36%). ^1H NMR (300 MHz, CD_3OD) δ 8.34 (s, 1H), 7.84 (dd, *J* = 8.1, 1.6 Hz, 1H), 7.39 – 7.27 (m, 2H), 7.08 – 6.84 (m, 5H), 5.28 (s, 2H), 4.66 – 4.52 (m, 1H), 4.20 (t, *J* = 5.1 Hz, 2H), 3.80 (t, *J* = 5.1 Hz, 2H), 3.58 – 3.42 (m, 18H), 2.80 (t, *J* = 5.3 Hz, 2H), 1.12 (d, *J* = 6.1 Hz, 6H). ^{13}C NMR (75 MHz, CD_3OD) δ 161.50, 159.99, 156.51, 155.38, 152.74, 151.09, 134.30, 132.66, 130.43, 127.71, 123.82, 120.04, 119.00, 118.12, 117.94, 113.08, 90.17, 70.85, 70.32, 70.22, 70.12, 70.06, 70.04, 69.98, 69.76, 69.24, 50.98, 50.76, 40.37, 20.59. HRMS (ESI-MS) *m/z*: [M+H$^+$] calculated for $C_{33}H_{46}ClN_6O_7^+$: 673.3111, found 673.3124.; $C_{33}H_{45}ClN_6O_7$ (673.21).

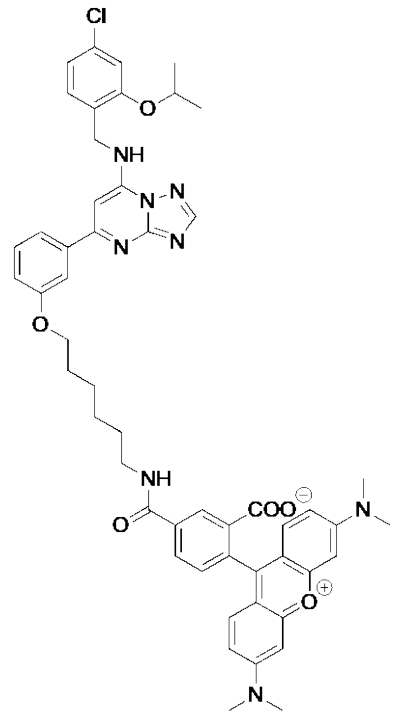

2-(3,6-Bis(dimethylamino)xanthylium-9-yl)-5-((6-(3-(7-((4-chloro-2-isopropoxybenzyl)amino)-[1,2,4]triazolo[1,5-*a*]pyrimidin-5-yl)phenoxy)hexyl)carbamoyl)benzoate hydrotrifluoroacetate (46)

The title compound was prepared from precursor **39** (5.40 mg, 0.0106 mmol, 1.5 eq), 5-TAMRA NHS ester (3.73 mg, 0.0071 mmol, 1 eq), and triethylamine (10.36 µL, 0.0778 mmol, 11 eq) according to the general procedure J (using method A for purification) yielding a fluffy purple solid (1.993 mg, 18%). RP-HPLC: 97% (t_R = 17.08 min, *k* = 4.69). ^{1}H NMR (400 MHz, CD_3OD) δ 8.73 (d, *J* = 1.7 Hz, 1H), 8.34 (s, 1H), 8.19 (dd, *J* = 7.9, 1.8 Hz, 1H), 7.49 – 7.40 (m, 3H), 7.31 – 7.24 (m, *J* = 8.6 Hz, 2H), 7.13 – 6.95 (m, 7H), 6.92 – 6.86 (m, *J* = 7.2, 5.4, 2.2 Hz, 2H), 6.70 (s, 1H), 5.28 (s, 2H), 4.59 – 4.51 (m, 1H), 3.85 (t, 2H), 3.48 – 3.43 (m, 2H), 3.30 (s*, 12H, concealed), 1.86 – 1.78 (m, 2H), 1.71 – 1.65 (m, 2H), 1.52 – 1.45 (m, 4H), 1.02 (d, *J* = 6.0 Hz, 6H). HRMS (ESI-MS) *m/z*: [M+H$^+$] calculated for $C_{52}H_{54}ClN_8O_6^+$: 921.3850, found 921.3849.; $C_{52}H_{53}ClN_8O_6$ x $C_2HF_3O_2$ (1035.52).

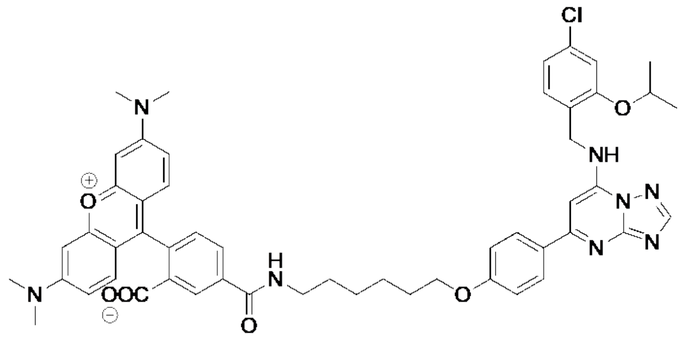

2-(3,6-Bis(dimethylamino)xanthylium-9-yl)-5-((6-(4-(7-((4-chloro-2-isopropoxybenzyl)amino)-[1,2,4]triazolo[1,5-*a*]pyrimidin-5-yl)phenoxy)hexyl)carbamoyl)benzoate hydrotrifluoroacetate (47)

The title compound was prepared from precursor **40** (0.65 mg, 0.0013 mmol, 1.4 eq), 5-TAMRA NHS ester (0.50 mg, 0.0009 mmol, 1 eq), and triethylamine (1.39 µL, 0.0104 mmol, 11 eq) according to the general procedure J (using method A for purification) yielding a fluffy purple solid (0.635 mg, 65%). RP-HPLC: > 99% (t_R = 15.87 min, *k* = 4.29). ^{1}H NMR (400 MHz, CD_3OD) δ 8.74 (s, 1H), 8.22 – 8.17 (m, 1H), 7.91 (d, *J* = 8.7 Hz, 2H), 7.47 (d, *J* = 7.9 Hz, 1H), 7.28 (d, *J* = 8.1 Hz, 1H), 7.11 (d, 2H), 7.05 – 6.97 (m, 5H), 6.91 – 6.84 (m, 4H), 6.70 (s, 1H), 5.26 (s, 2H), 4.57 – 4.51 (m, 1H), 3.83 (t, *J* = 7.7 Hz, 2H), 3.47 (m, 2H), 3.30 (s*, 12H, concealed), 1.85 – 1.77 (m, 2H), 1.71 – 1.66 (m, 2H), 1.50 – 1.45 (m, 4H), 1.02 (d, *J* = 6.0 Hz, 6H). HRMS (ESI-MS) *m/z*: [M+H$^+$] calculated for $C_{52}H_{54}ClN_8O_6^+$: 921.3850, found 921.3849.; $C_{52}H_{53}ClN_8O_6$ x $C_2HF_3O_2$ (1035.52).

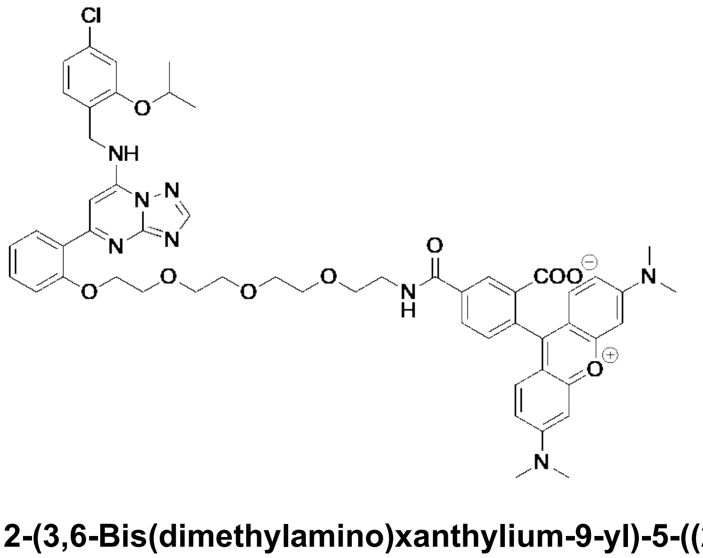

2-(3,6-Bis(dimethylamino)xanthylium-9-yl)-5-((2-(2-(2-(2-(2-(7-((4-chloro-2-isopropoxybenzyl)amino)-[1,2,4]triazolo[1,5-*a*]pyrimidin-5-yl)phenoxy)ethoxy)ethoxy)ethoxy)ethyl)carbamoyl)benzoate hydrotrifluoroacetate (48)

The title compound was prepared from precursor **41** (1.92 mg, 0.0050 mmol, 3 eq), 5-TAMRA NHS ester (0.60 mg, 0.0011 mmol, 1 eq), and triethylamine (1.67 µL, 0.0125 mmol, 11 eq) according to the general procedure J (using method A for purification) yielding a fluffy purple solid (1,091 mg, 86%). RP-HPLC: > 99% (t_R = 16.95 min, *k* = 4.65). ^{1}H NMR (400 MHz, CD_3OD) δ 8.71 (d, *J* = 1.8 Hz, 1H), 8.24 – 8.19 (m, 2H), 7.75

(dd, *J* = 8.2, 1.6 Hz, 1H), 7.40 (d, *J* = 7.9 Hz, 1H), 7.31 – 7.26 (m, 1H), 7.23 (d, *J* = 8.2 Hz, 1H), 6.99 (d, *J* = 9.4 Hz, 2H), 6.95 (d, *J* = 1.9 Hz, 1H), 6.91 – 6.80 (m, 8H), 5.23 (s, 2H), 4.56 – 4.48 (m, 1H), 4.06 (t, *J* = 5.3 Hz, 2H), 3.71 (t, *J* = 5.2 Hz, 2H), 3.63 – 3.56 (m, 4H), 3.54 – 3.48 (m, 8H), 3.22 (s, 12H), 1.02 (d, *J* = 6.0 Hz, 6H). HRMS (ESI-MS) *m/z*: [M+H$^+$] calculated for $C_{54}H_{58}ClN_8O_9^+$: 997.4010, found 997.4006.; $C_{54}H_{57}ClN_8O_9$ x $C_2HF_3O_2$ (1111.57).

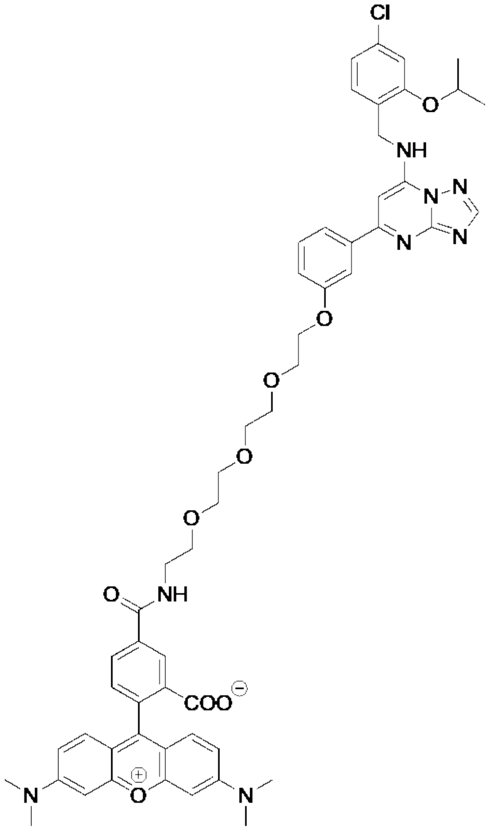

2-(3,6-Bis(dimethylamino)xanthylium-9-yl)-5-((2-(2-(2-(2-(3-(7-((4-chloro-2-isopropoxybenzyl)amino)-[1,2,4]triazolo[1,5-*a*]pyrimidin-5-yl)phenoxy)ethoxy)ethoxy)ethoxy)ethyl)carbamoyl)benzoate hydrotrifluoroacetate (49)

The title compound was prepared from precursor **42** (1.35 mg, 0.0023 mmol, 1 eq), 5-TAMRA NHS ester (1.22 mg, 0.0023 mmol, 1 eq), and triethylamine (3.38 µL, 0.0254 mmol, 11 eq) according to the general procedure J (using method A for purification) yielding a fluffy purple solid (1.508 mg, 59%). RP-HPLC: > 99% (t_R = 15.20 min, *k* = 4.07). ^{1}H NMR (400 MHz, CD_3OD) δ 8.70 (s, 1H), 8.25 (s, 1H), 8.19 (d, *J* = 7.6 Hz, 1H), 7.44 – 7.38 (m, 3H), 7.27 – 7.21 (m, 2H), 7.06 (d, *J* = 9.6 Hz, 2H), 6.99 – 6.83 (m, 7H), 6.79 (s, 1H), 5.22 (s, 2H), 4.60 – 4.51 (m, 1H), 4.08 (t, *J* = 5.3 Hz, 2H), 3.74 (t, *J* = 5.2 Hz, 2H), 3.68 – 3.60 (m, 4H), 3.58 – 3.50 (m, 8H), 3.24 (s, 12H), 1.04 (d, *J* = 6.0 Hz, 6H). HRMS (ESI-MS) *m/z*: [M+H$^+$] calculated for $C_{54}H_{58}ClN_8O_9^+$: 997.4010, found 997.4020.; $C_{54}H_{57}ClN_8O_9$ x $C_2HF_3O_2$ (1111.57).

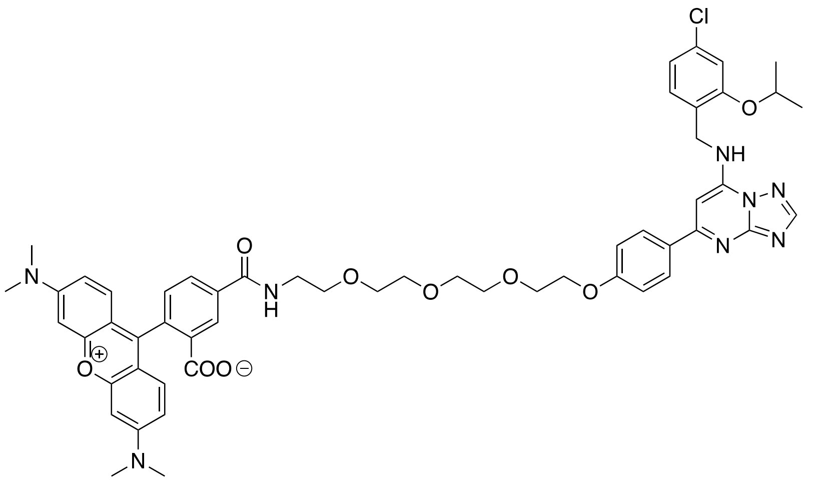

2-(3,6-Bis(dimethylamino)xanthylium-9-yl)-5-((2-(2-(2-(2-(4-(7-((4-chloro-2-isopropoxybenzyl)amino)-[1,2,4]triazolo[1,5-*a*]pyrimidin-5-yl)phenoxy)ethoxy)ethoxy)ethoxy)ethyl)carbamoyl)benzoate hydrotrifluoroacetate (50)

The title compound was prepared from precursor **43** (2.90 mg, 0.0050 mmol, 2.2 eq), 5-TAMRA NHS ester (1.20 mg, 0.0023 mmol, 1 eq), and triethylamine (3.33 µL, 0.0250 mmol, 11 eq) according to the general procedure J (using method A for purification) yielding a fluffy purple solid (2.231 mg, 88%). RP-HPLC: > 99% (t_R = 15.22 min, *k* = 4.07). ^{1}H NMR (400 MHz, CD_3OD) δ 8.75 (d, *J* = 1.8 Hz, 1H), 8.24 (dd, *J* = 7.9, 1.8 Hz, 1H), 7.89 (d, 2H), 7.45 (d, *J* = 7.9 Hz, 1H), 7.25 (d, *J* = 8.1 Hz, 1H), 7.04 (d, *J* = 9.4 Hz, 2H), 6.99 – 6.76 (m, 10H), 5.21 (s, 2H), 4.60 – 4.50 (m, 1H), 4.04 (t, *J* = 5.3 Hz, 2H), 3.74 (t, *J* = 5.2 Hz, 2H), 3.68 – 3.61 (m, 4H), 3.58 – 3.53 (m, 8H), 3.25 (s, 12H), 1.05 (d, *J* = 6.0 Hz, 6H). HRMS (ESI-MS) *m/z*: [M+H$^+$] calculated for $C_{54}H_{58}ClN_8O_9^+$: 997.4010, found 997.4012.; $C_{54}H_{57}ClN_8O_9$ x $C_2HF_3O_2$ (1111.57).

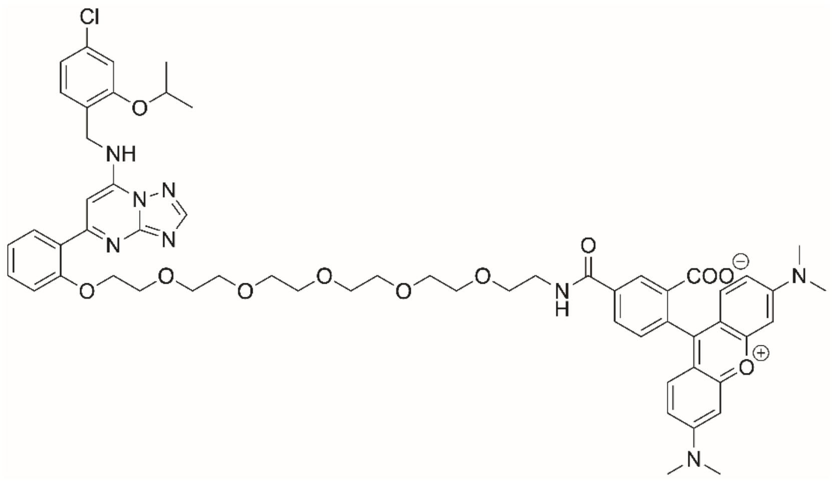

2-(3,6-Bis(dimethylamino)xanthylium-9-yl)-5-((17-(2-(7-((4-chloro-2-isopropoxybenzyl)amino)-[1,2,4]triazolo[1,5-*a*]pyrimidin-5-yl)phenoxy)-3,6,9,12,15-pentaoxaheptadecyl)carbamoyl)benzoat hydrotrifluoroacetate (51)

The title compound was prepared from precursor **44** (18.60 mg, 0.0365 mmol, 3.1 eq), 5-TAMRA NHS ester (6.20 mg, 0.0118 mmol, 1 eq), and triethylamine (17.21 µL,

0.1293 mmol, 11 eq) according to the general procedure J (using method A for purification) yielding a fluffy purple solid (9.34 mg, 66%). RP-HPLC: 98% (t_R = 16.80 min, k = 4.60). ^{1}H NMR (400 MHz, CD_3OD) δ 8.82 (d, J = 1.8 Hz, 1H), 8.40 (s, 1H), 8.32 (dd, J = 7.9, 1.9 Hz, 1H), 7.85 (d, J = 8.0 Hz, 1H), 7.52 (d, J = 7.9 Hz, 1H), 7.39 – 7.30 (m, 2H), 7.11 (d, J = 1.0 Hz, 2H), 7.04 (s, 2H), 7.01 – 6.88 (m, 7H), 5.31 (s, 2H), 4.68 – 4.57 (m, 1H), 4.16 (t, J = 5.1 Hz, 2H), 3.80 (t, J = 5.1 Hz, 2H), 3.76 – 3.49 (m, 20H), 3.30 (s, 12H), 1.15 (d, J = 6.0 Hz, 6H). ^{13}C NMR (101 MHz, CD_3OD) δ 166.74, 165.93, 161.35, 159.82, 157.49, 157.41, 156.53, 152.31, 151.06, 136.64, 136.28, 134.37, 132.73, 131.44, 131.01, 130.57, 130.43, 130.03, 127.80, 123.72, 120.06, 119.06, 118.08, 114.08, 113.24, 113.14, 96.04, 90.39, 70.39, 70.31, 70.23, 70.19, 70.11, 70.03, 69.93, 69.09, 51.05, 50.73, 39.89, 39.52, 20.59. HRMS (ESI-MS) *m/z*: [M+H$^+$] calculated for $C_{58}H_{66}ClN_8O_{11}^+$: 1085.4534, found 1085.4548.; $C_{58}H_{65}ClN_8O_{11}$ x $C_2HF_3O_2$ (1199.68).

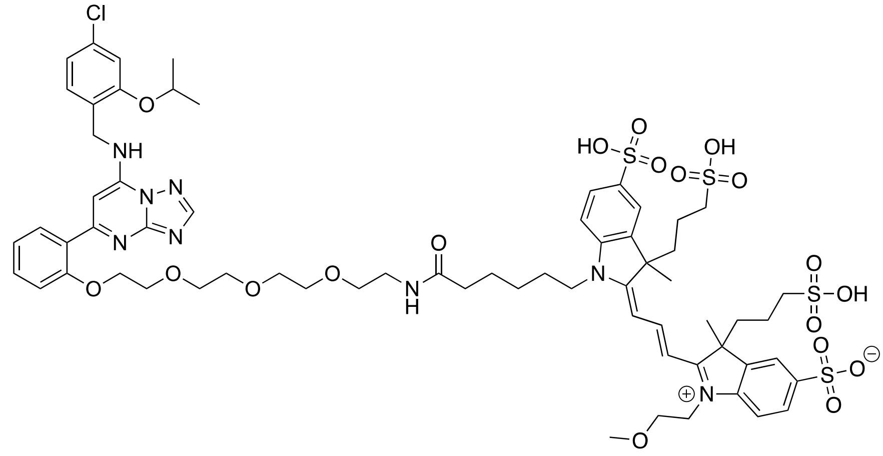

Triammonium 2-((*E*)-3-((*E*)-1-(1-(2-(7-((4-chloro-2-isopropoxybenzyl)amino)-[1,2,4]triazolo[1,5-*a*]pyrimidin-5-yl)phenoxy)-13-oxo-3,6,9-trioxa-12-azaoctadecan-18-yl)-3-methyl-5-sulfo-3-(3-sulfopropyl)indolin-2-ylidene)prop-1-en-1-yl)-1-(2-methoxyethyl)-3-methyl-5-sulfo-3-(3-sulfopropyl)-3*H*-indol-1-ium (52)

The title compound was prepared from precursor **41** (8.06 mg, 0.0138 mmol, 71.6 eq), DY-549P1 NHS ester (0.20 mg, 0.0002 mmol, 1 eq), and triethylamine (0.28 µL, 0.0021 mmol, 11 eq) according to the general procedure J (using method B for purification) yielding a fluffy purple solid (0.25 mg, 84%). RP-HPLC: > 99% (t_R = 6.44 min, k = 1.34). HRMS (ESI-MS) *m/z*: [M+H$^+$] calculated for $C_{65}H_{84}ClN_8O_{19}S_4^+$: 1443.4419, found 1443.4405.; $C_{65}H_{83}ClN_8O_{19}S_4$ x 3 NH_3 (1495.20).

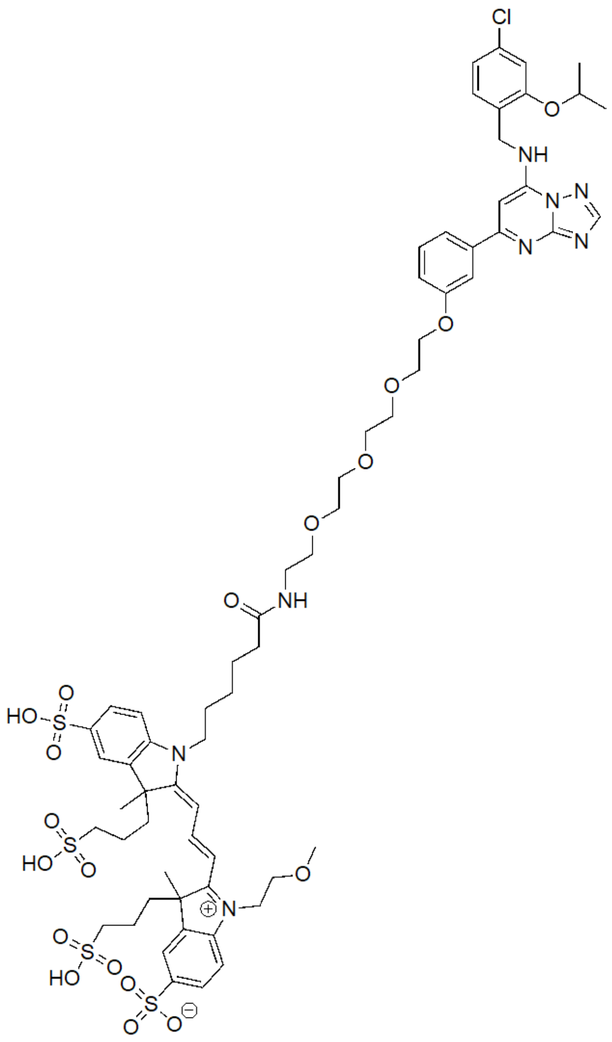

Triammonium 2-((*E*)-3-((*E*)-1-(1-(3-(7-((4-chloro-2-isopropoxybenzyl)amino)-[1,2,4]triazolo[1,5-*a*]pyrimidin-5-yl)phenoxy)-13-oxo-3,6,9-trioxa-12-azaoctadecan-18-yl)-3-methyl-5-sulfo-3-(3-sulfopropyl)indolin-2-ylidene)prop-1-en-1-yl)-1-(2-methoxyethyl)-3-methyl-5-sulfo-3-(3-sulfopropyl)-3*H*-indol-1-ium (53)

The title compound was prepared from precursor **42** (0.58 mg, 0.0010 mmol, 5.2 eq), DY-549P1 NHS ester (0.20 mg, 0.0002 mmol, 1 eq), and triethylamine (0.28 µL, 0.0021 mmol, 11 eq) according to the general procedure J (using method B for purification) yielding a fluffy purple solid (0.299 mg, 100%). RP-HPLC: > 99% (t_R = 5.70 min, k = 0.9). HRMS (ESI-MS) *m/z*: $[M-2H]^{2-}$ calculated for $C_{65}H_{81}ClN_8O_{19}S_4^{2-}$: 720.2100, found 720.2114.; $C_{65}H_{83}ClN_8O_{19}S_4$ x 3 NH_3 (1495.20).

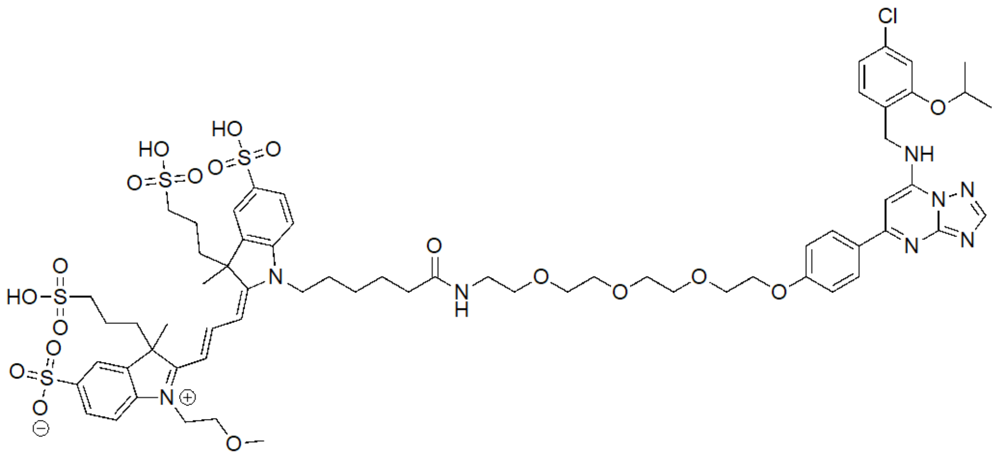

Triammonium 2-((*E*)-3-((*E*)-1-(1-(4-(7-((4-chloro-2-isopropoxybenzyl)amino)-[1,2,4]triazolo[1,5-*a*]pyrimidin-5-yl)phenoxy)-13-oxo-3,6,9-trioxa-12-azaoctadecan-18-yl)-3-methyl-5-sulfo-3-(3-sulfopropyl)indolin-2-ylidene)prop-1-en-1-yl)-1-(2-methoxyethyl)-3-methyl-5-sulfo-3-(3-sulfopropyl)-3*H*-indol-1-ium (54)

The title compound was prepared from precursor **43** (0.45 mg, 0.0008 mmol, 4 eq), DY-549P1 NHS ester (0.20 mg, 0.0002 mmol, 1 eq), and triethylamine (0.28 µL, 0.0021 mmol, 11 eq) according to the general procedure J (using method B for purification) yielding a fluffy purple solid (0.290 mg, 97%). RP-HPLC: > 99% (t_R = 4.92 min, *k* = 0.64). HRMS (ESI-MS) *m/z*: $[M-2H]^{2-}$ calculated for $C_{65}H_{81}ClN_8O_{19}S_4^{2-}$: 720.2100, found 720.2109.; $C_{65}H_{83}ClN_8O_{19}S_4$ x 3 NH_3 (1495.20).

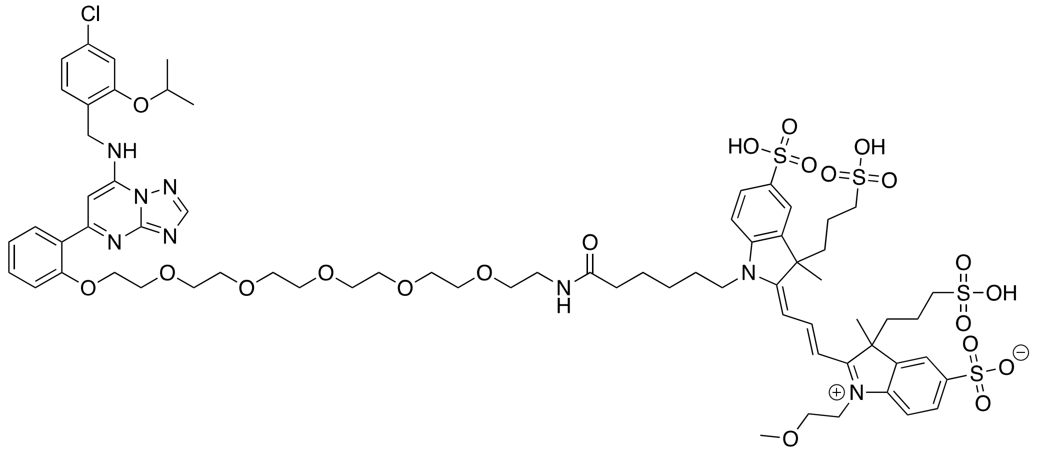

Triammonium 2-((*E*)-3-((*E*)-1-(1-(2-(7-((4-chloro-2-isopropoxybenzyl)amino)-[1,2,4]triazolo[1,5-*a*]pyrimidin-5-yl)phenoxy)-19-oxo-3,6,9,12,15-pentaoxa-18-azatetracosan-24-yl)-3-methyl-5-sulfo-3-(3-sulfopropyl)indolin-2-ylidene)prop-1-en-1-yl)-1-(2-methoxyethyl)-3-methyl-5-sulfo-3-(3-sulfopropyl)-3*H*-indol-1-ium (55)

The title compound was prepared from precursor **44** (2.09 mg, 0.0027 mmol, 14 eq), DY-549P1 NHS ester (0.20 mg, 0.0002 mmol, 1 eq), and triethylamine (0.28 µL, 0.0021 mmol, 11 eq) according to the general procedure J (using method B for purification) yielding a fluffy purple solid (0.317 mg, 100%). RP-HPLC: > 99% (t_R = 6.64 min, *k* = 1.41). HRMS (ESI-MS) *m/z*: $[M+H^+]$ calculated for $C_{69}H_{92}ClN_8O_{21}S_4^+$: 1531.4943, found 1531.4935.; $C_{69}H_{91}ClN_8O_{21}S_4$ x 3 NH_3 (1583.31).

3. Chemical structures of 7-10, 13, 16, 19, 22, 25, 28, 31, 38, and 45

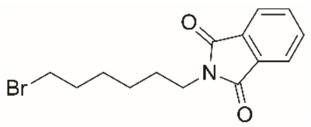

2-(6-Bromohexyl)isoindoline-1,3-dione (7)[10]

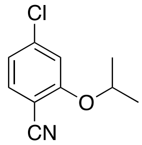

4-Chloro-2-isopropoxybenzonitrile (8)

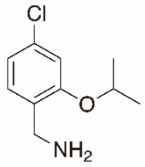

4-Chloro-2-isopropoxyphenylmethanamine (9)[11]

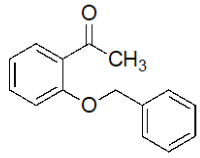

1-(2-(Benzyloxy)phenyl)ethan-1-one (10)[12]

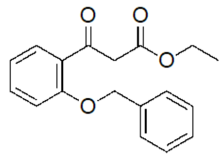

Ethyl 3-(2-(benzyloxy)phenyl)-3-oxopropanoate (13)[13]

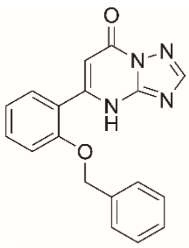

5-(2-(Benzyloxy)phenyl)-[1,2,4]triazolo[1,5-*a*]pyrimidin-7(4*H*)-one (16)

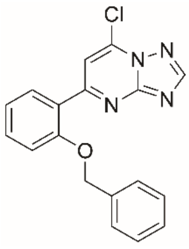

5-(2-(Benzyloxy)phenyl)-7-chloro-[1,2,4]triazolo[1,5-*a*]pyrimidine (19)

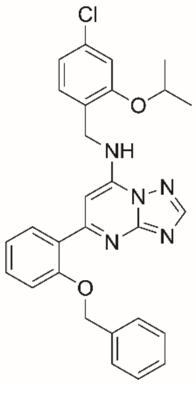

5-(2-(Benzyloxy)phenyl)-*N*-(4-chloro-2-isopropoxybenzyl)-[1,2,4]triazolo[1,5-*a*]pyrimidin-7-amine (22)

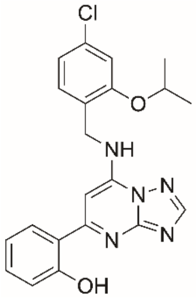

2-(7-((4-Chloro-2-isopropoxybenzyl)amino)-[1,2,4]triazolo[1,5-*a*]pyrimidin-5-yl)phenol (25)

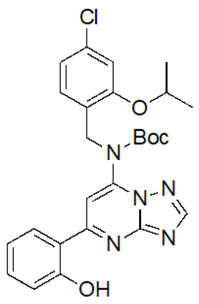

***tert*-Butyl (4-chloro-2-isopropoxybenzyl)(5-(2-hydroxyphenyl)-[1,2,4]triazolo[1,5-*a*]pyrimidin-7-yl)carbamate (28)**

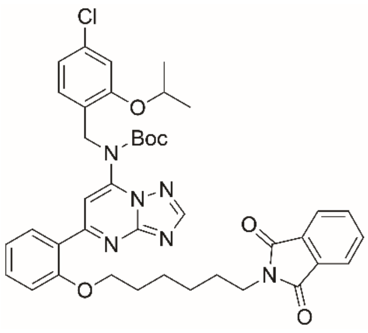

***tert*-Butyl (4-chloro-2-isopropoxybenzyl)(5-(2-((6-(1,3-dioxoisoindolin-2-yl)hexyl)oxy)phenyl)-[1,2,4]triazolo[1,5-*a*]pyrimidin-7-yl)carbamate (31)**

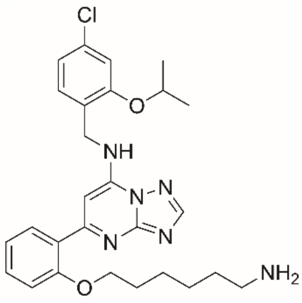

5-(2-((6-Aminohexyl)oxy)phenyl)-*N*-(4-chloro-2-isopropoxybenzyl)-[1,2,4]triazolo[1,5-*a*]pyrimidin-7-amine dihydrotrifluoroacetate (38)

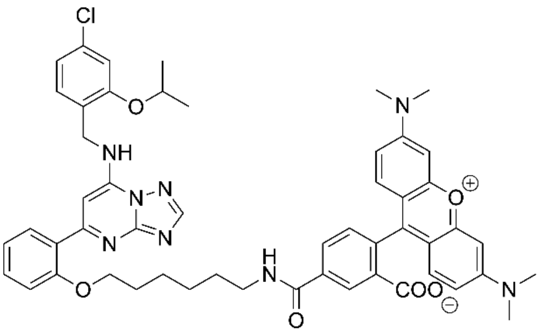

2-(3,6-Bis(dimethylamino)xanthylium-9-yl)-5-((6-(2-(7-((4-chloro-2-isopropoxybenzyl)amino)-[1,2,4]triazolo[1,5-*a*]pyrimidin-5-yl)phenoxy)hexyl)carbamoyl)benzoate hydrotrifluoroacetate (45)

4. NMR spectra of compounds 38-44 and 45-51

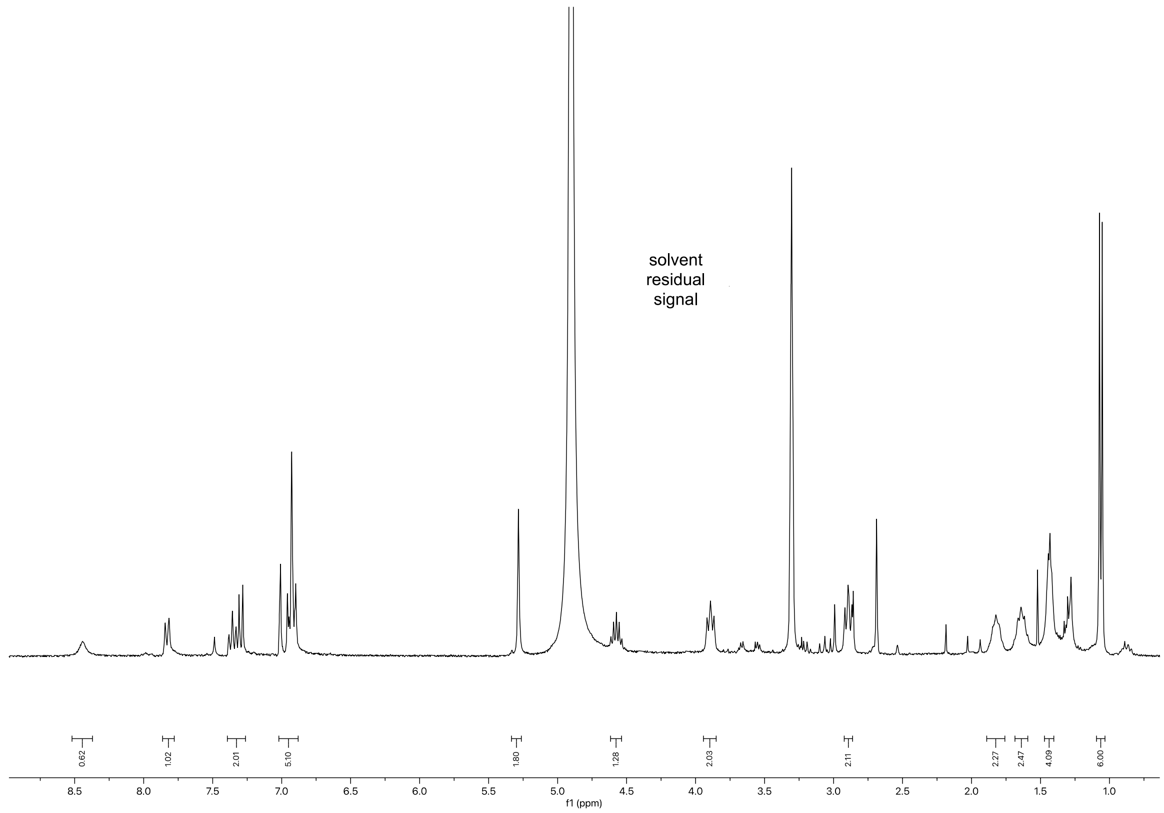

Figure S1. ^{1}H NMR spectrum (300 MHz, CD_3OD) of compound **38**.

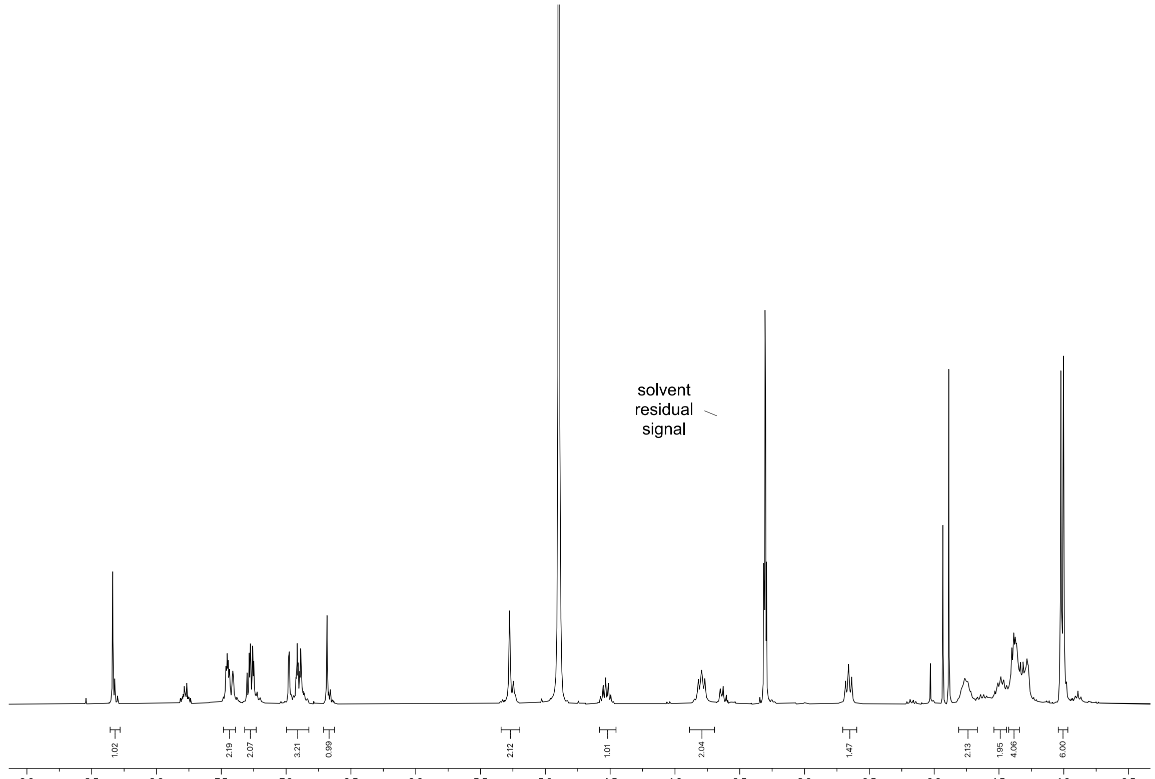

Figure S2. ^{1}H NMR spectrum (300 MHz, CD_3OD) of compound **39**.

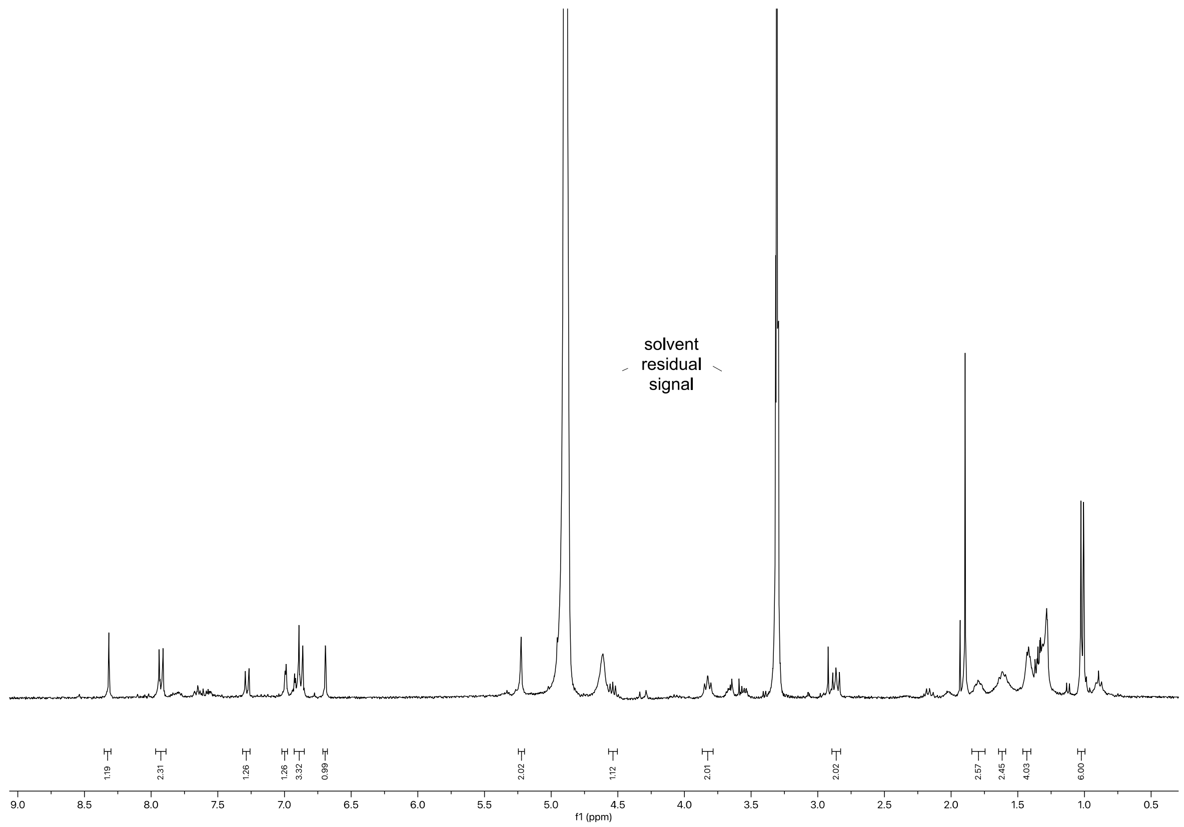

Figure S3. ^{1}H NMR spectrum (300 MHz, CD_3OD) of compound **40**.

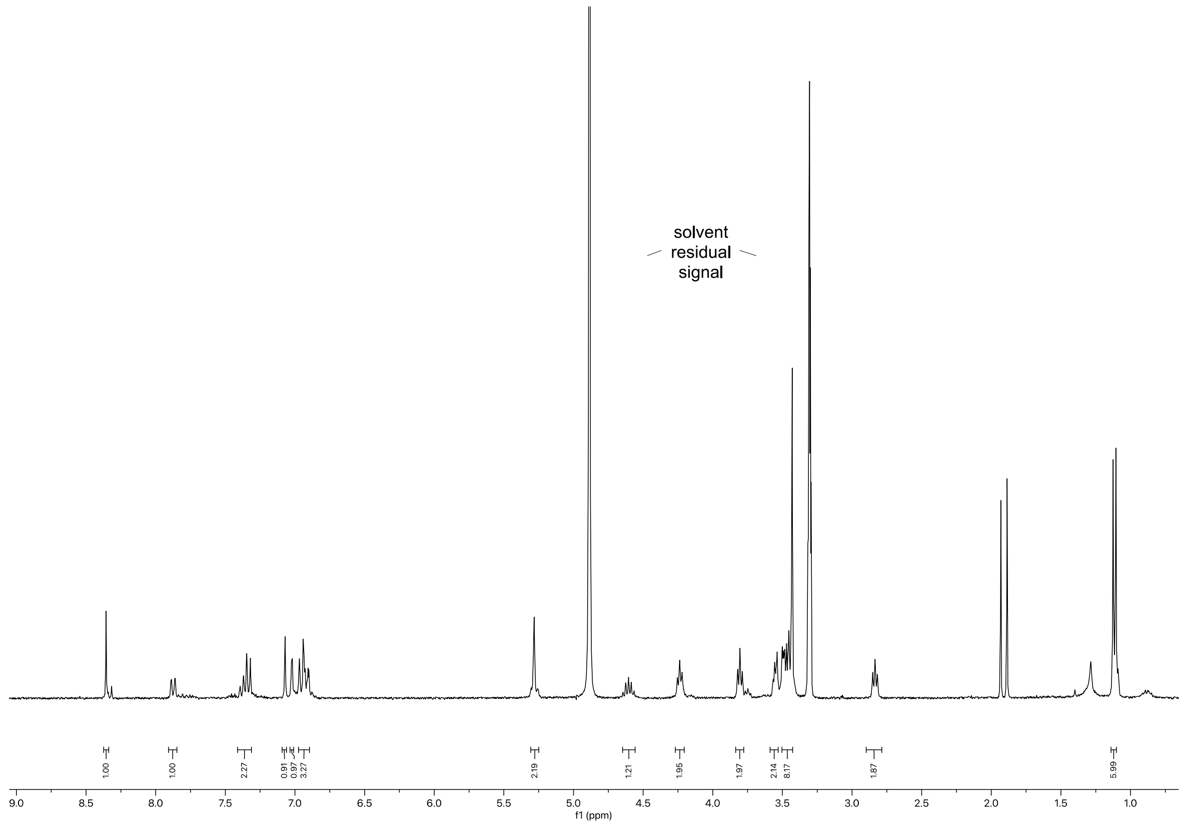

Figure S4. ^{1}H NMR spectrum (300 MHz, CD_3OD) of compound **41**.

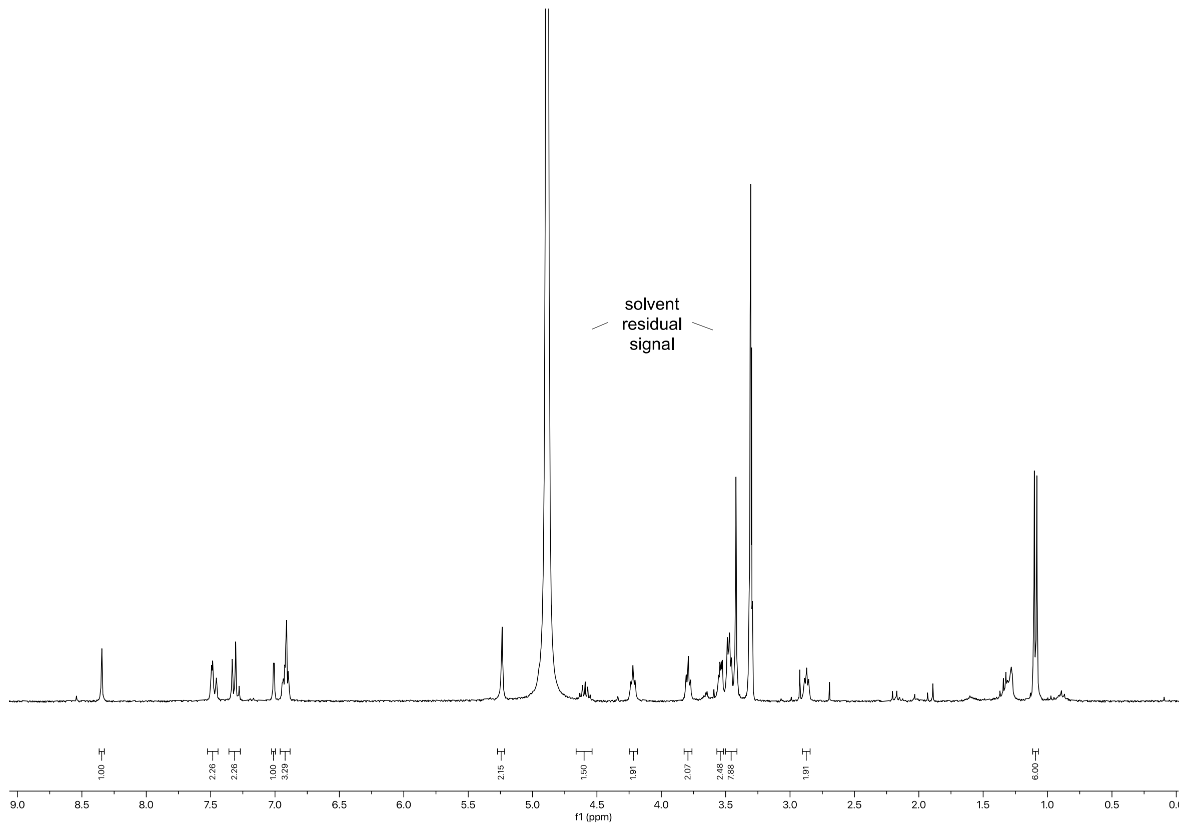

Figure S5. ^{1}H NMR spectrum (300 MHz, CD_3OD) of compound **42**.

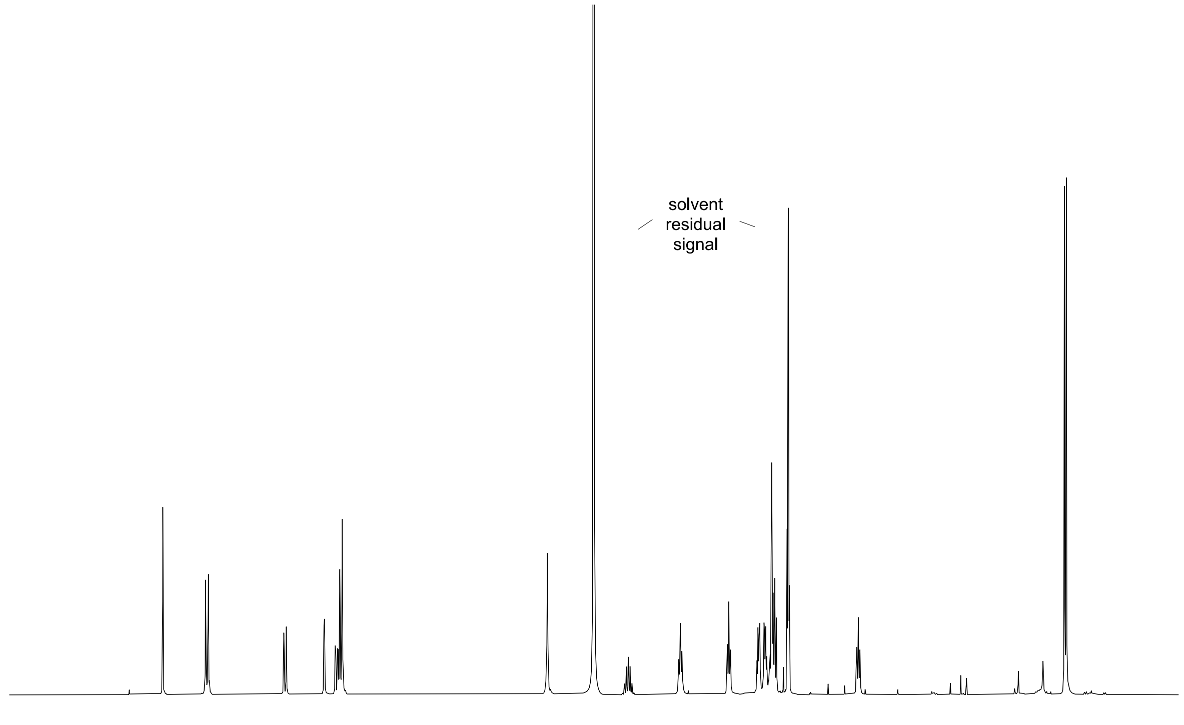

Figure S6. ^{1}H NMR spectrum (400 MHz, CD_3OD) of compound **43**.

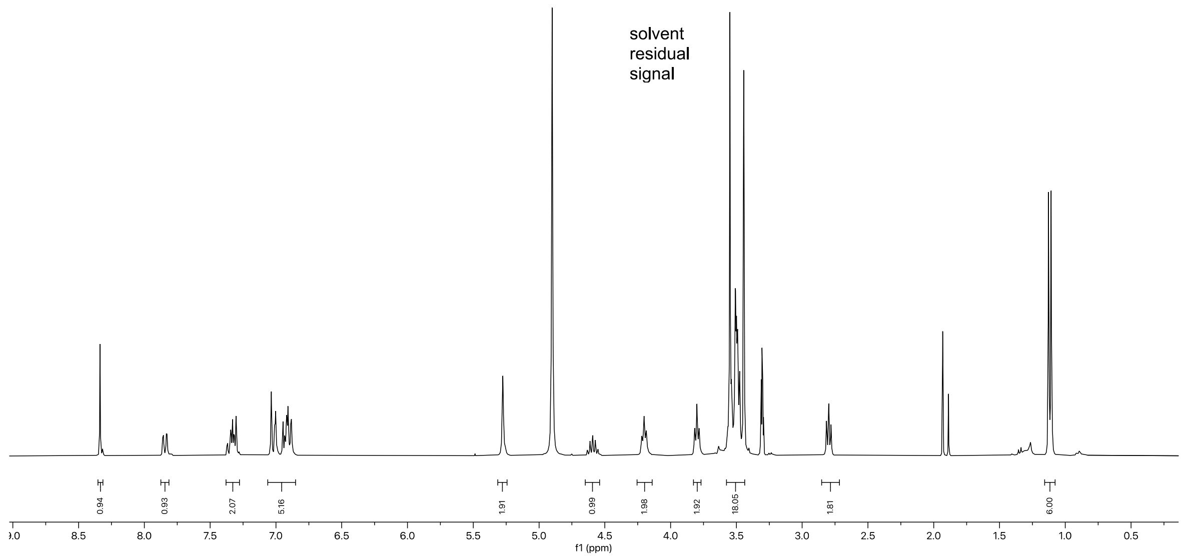

Figure S7. ^{1}H NMR spectrum (300 MHz, CD_3OD) of compound **44**.

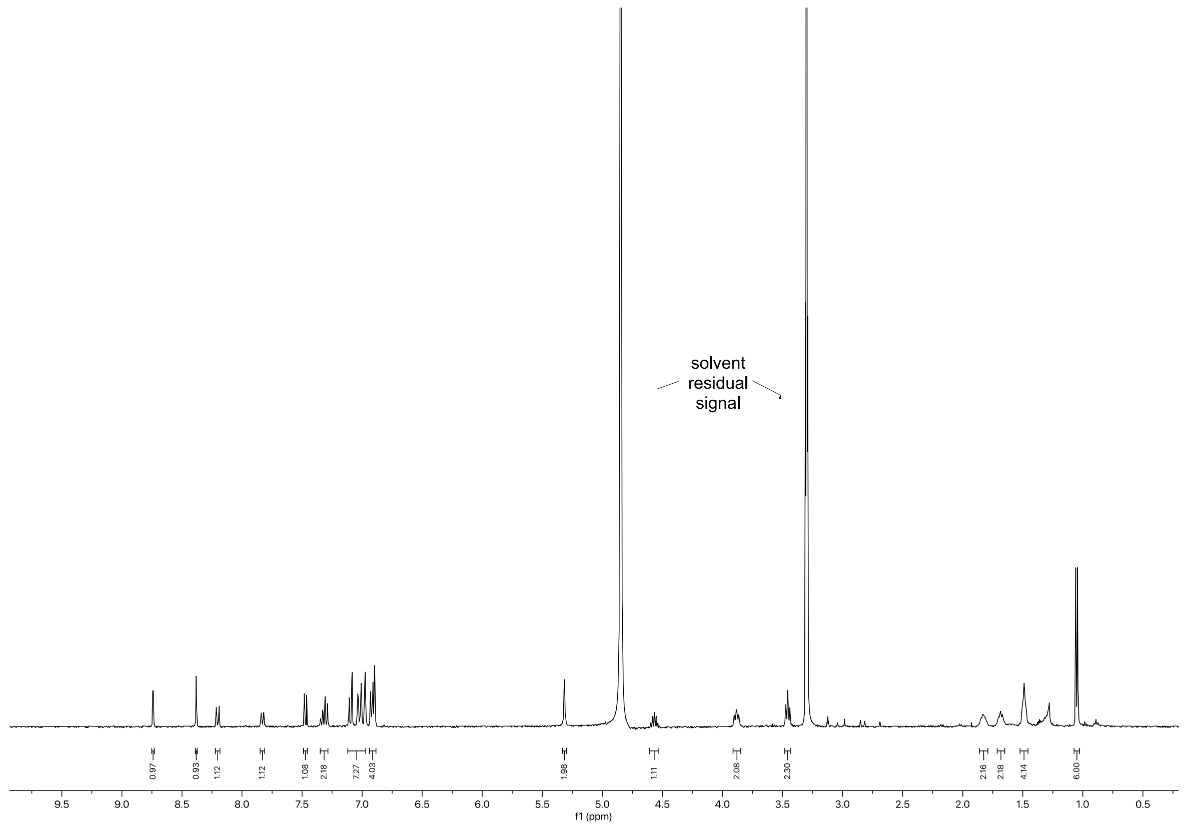

Figure S8. ^{1}H NMR spectrum (400 MHz, CD_3OD) of compound **45**.

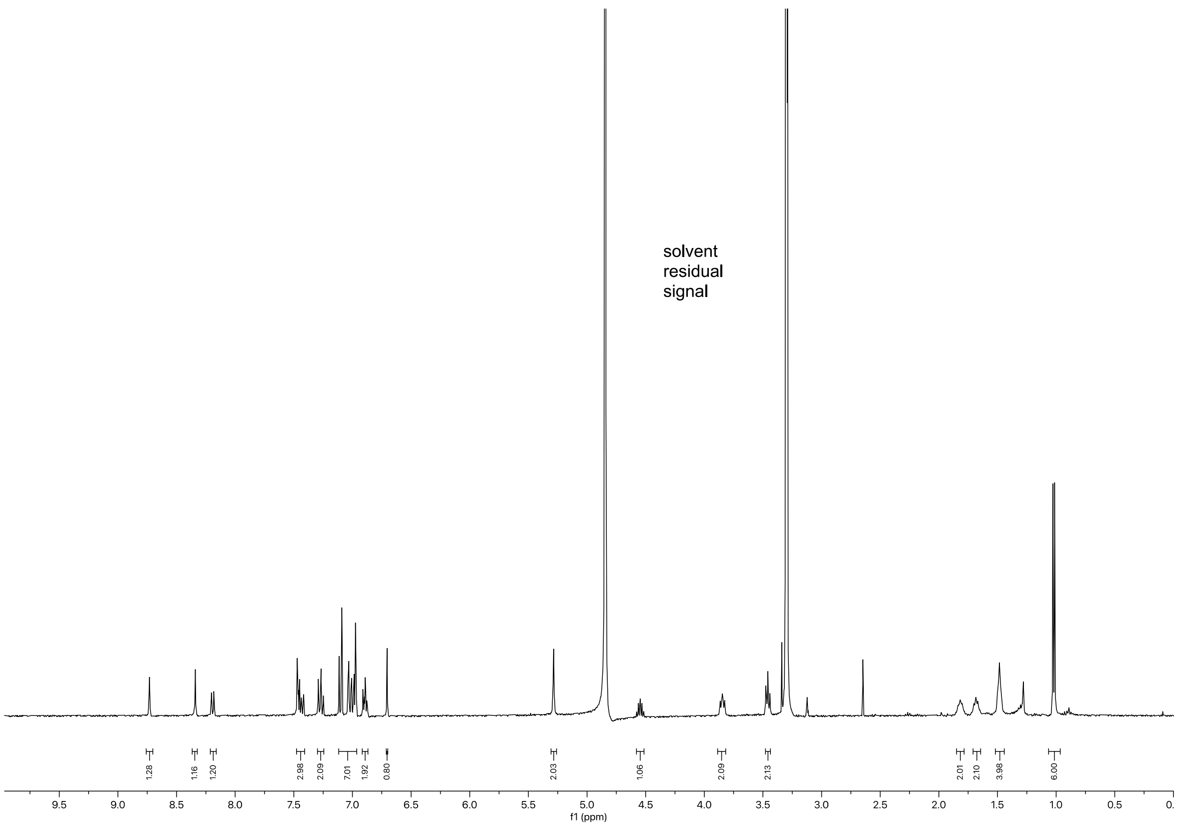

Figure S9. ^{1}H NMR spectrum (400 MHz, CD_3OD) of compound **46**.

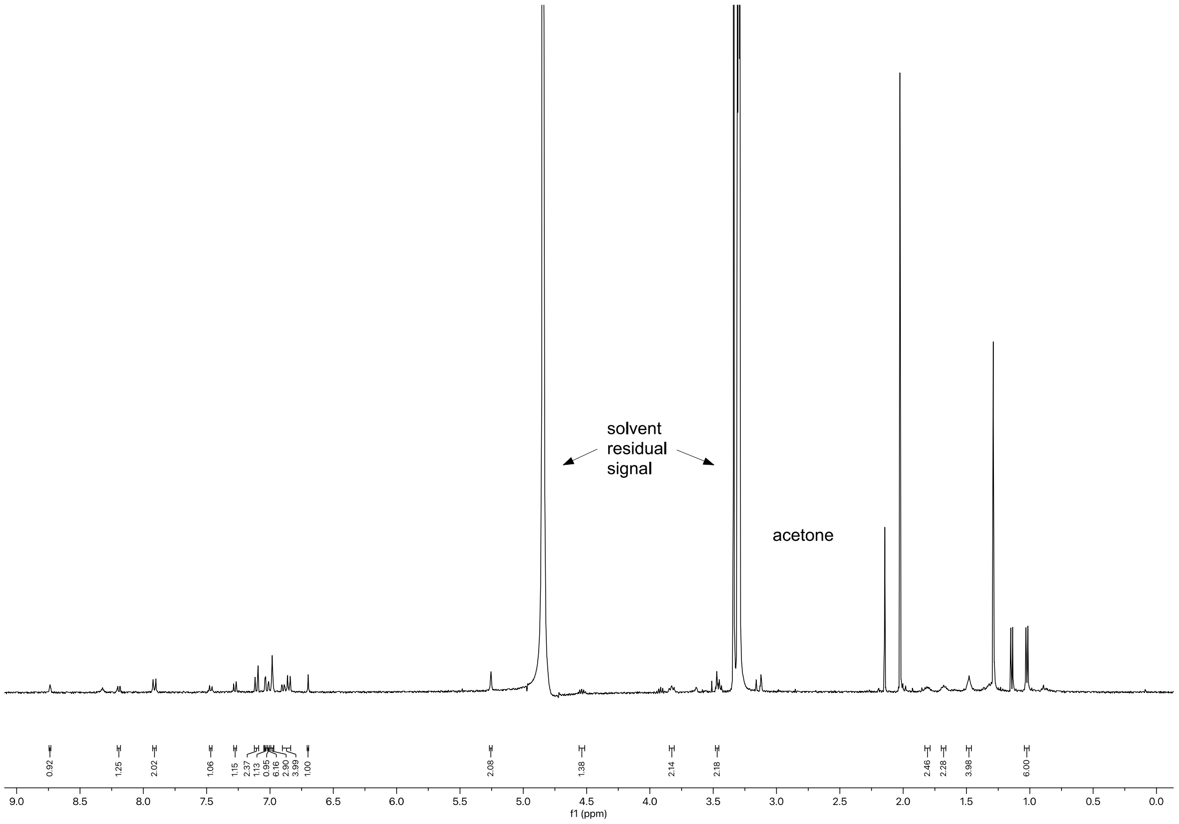

Figure S10. ^{1}H NMR spectrum (400 MHz, CD_3OD) of compound **47**.

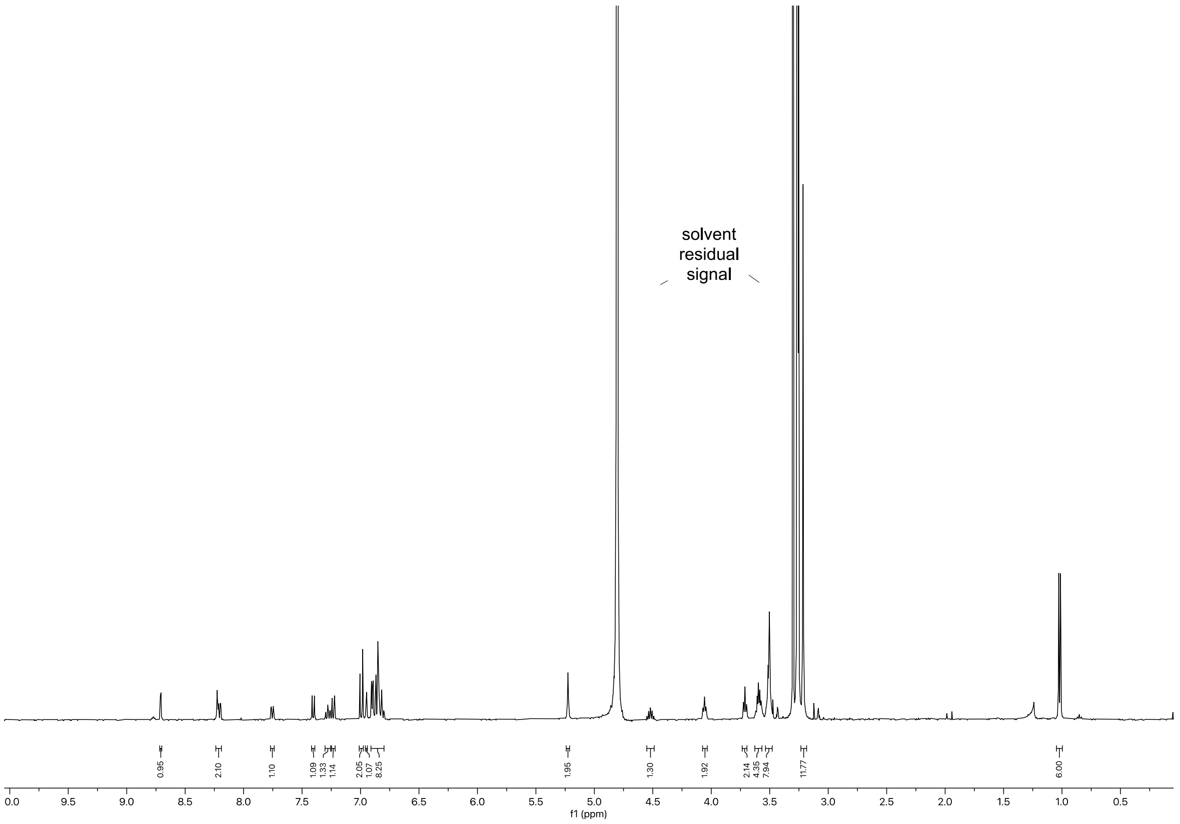

Figure S11. ^{1}H NMR spectrum (400 MHz, CD_3OD) of compound **48**.

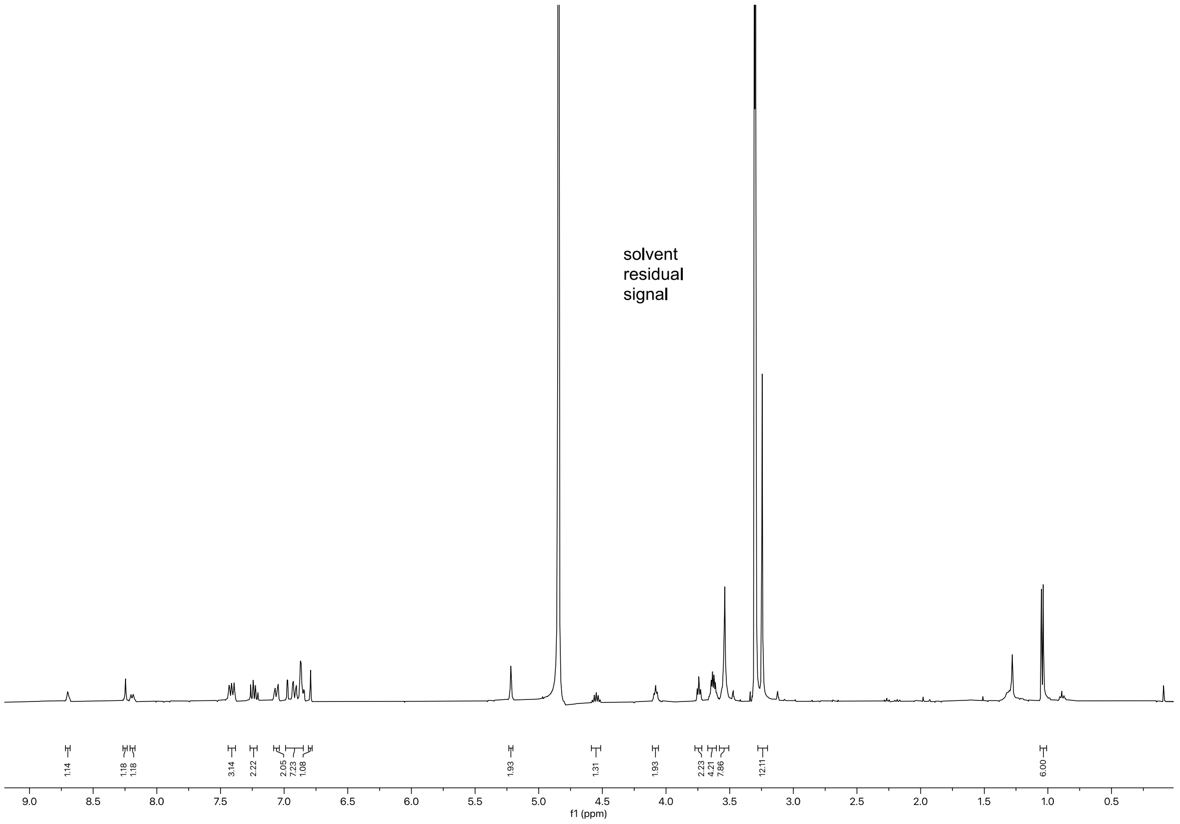

Figure S12. ^{1}H NMR spectrum (400 MHz, CD_3OD) of compound **49**.

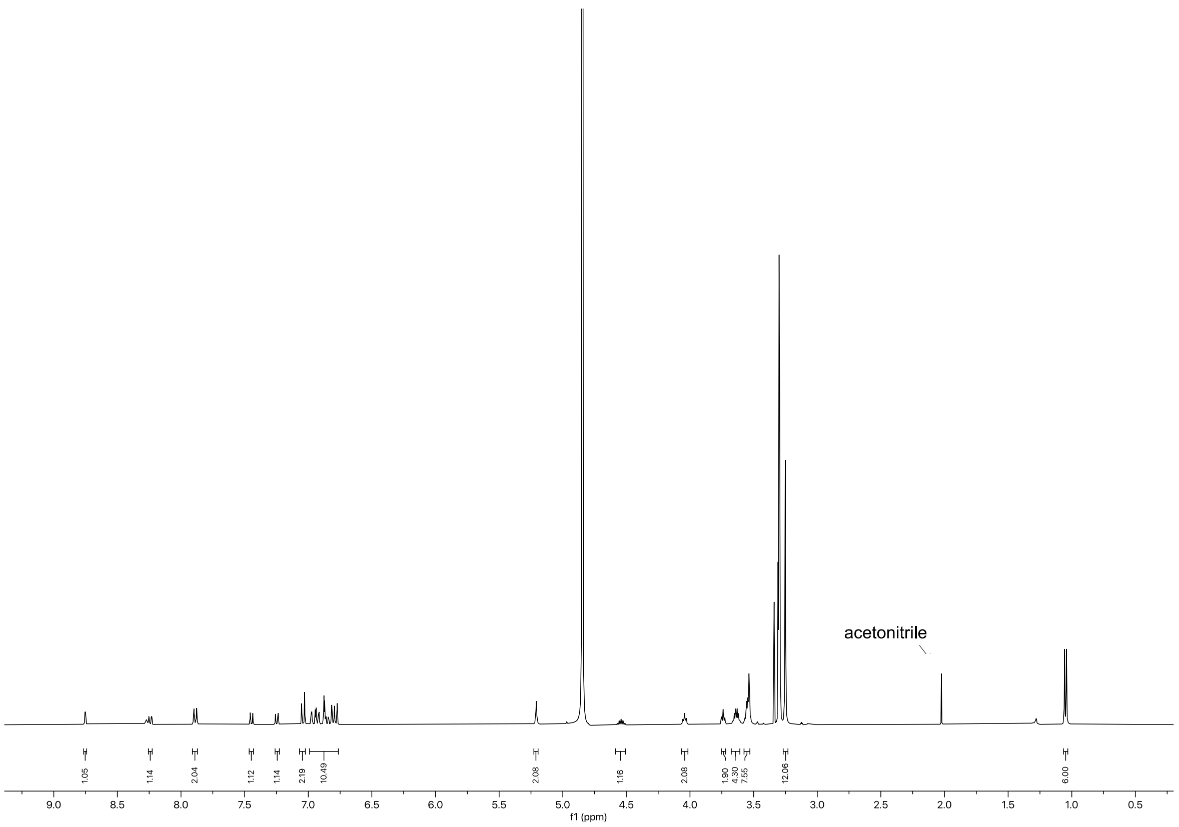

Figure S13. ^{1}H NMR spectrum (400 MHz, CD_3OD) of compound **50**.

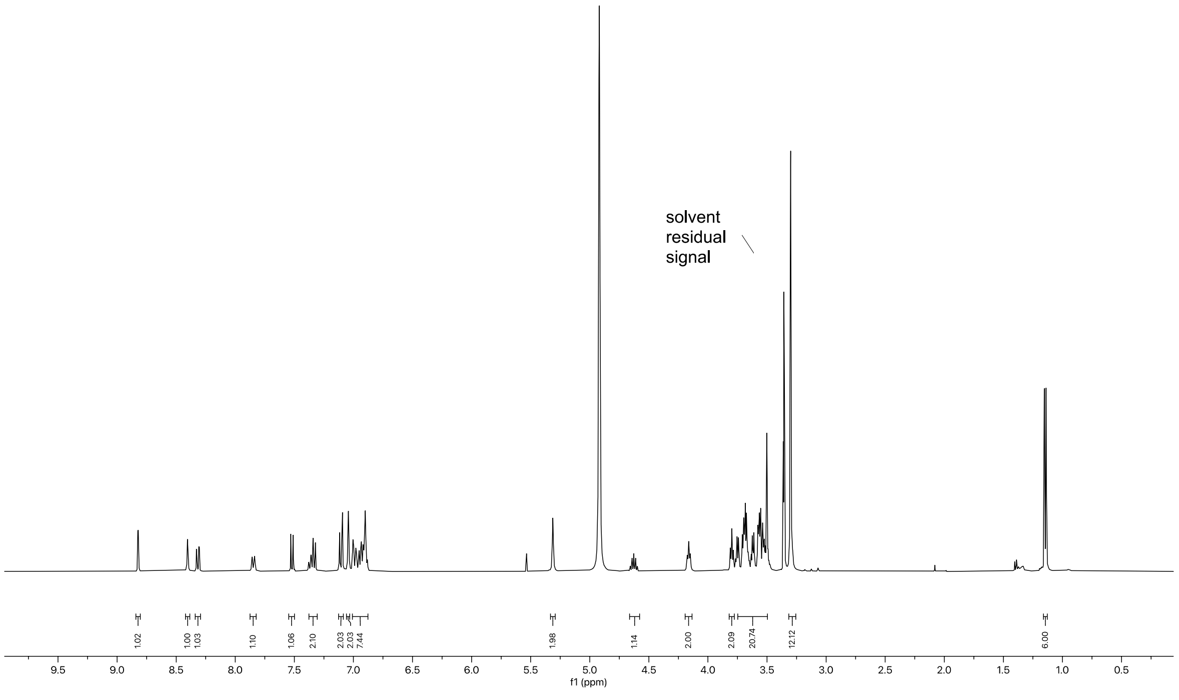

Figure S14. ^{1}H NMR spectrum (400 MHz, CD_3OD) of compound **51**.

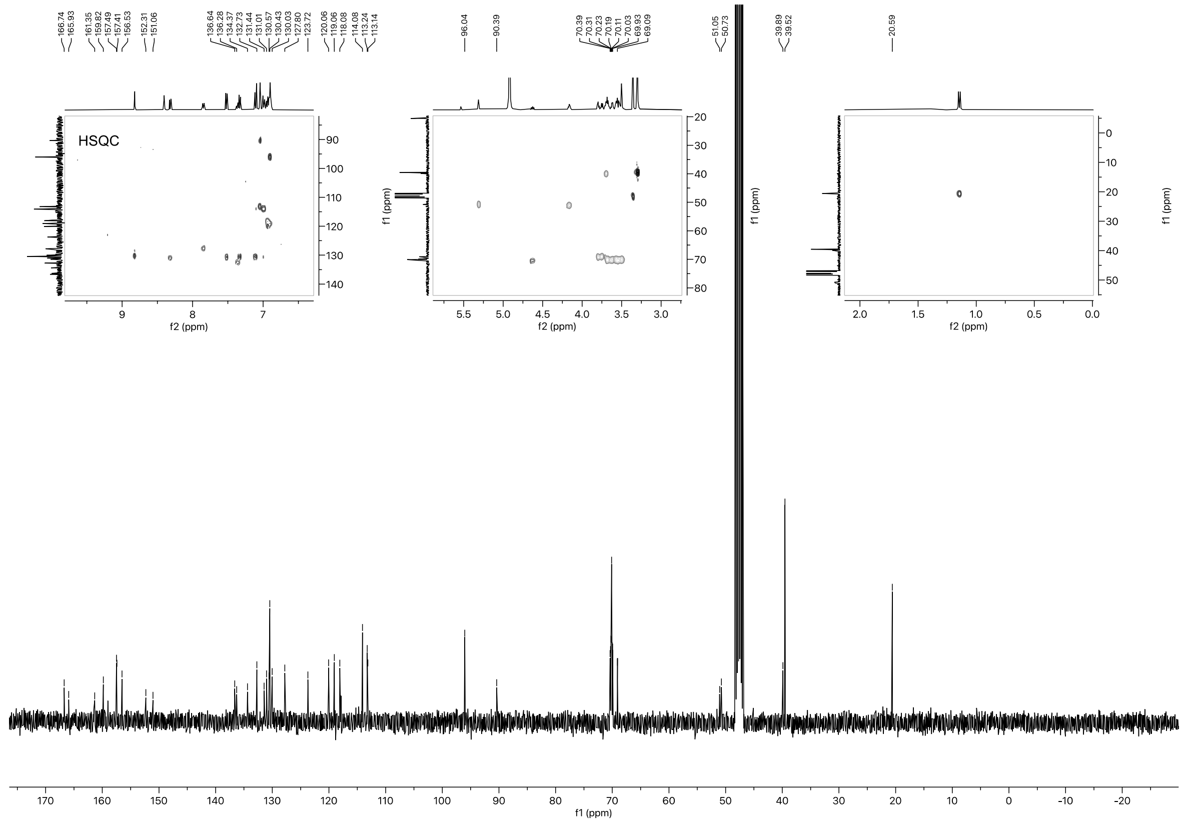

Figure S15. ^{13}C NMR spectrum (101 MHz, CD_3OD) of compound **51**.

5. RP-HPLC purity control of compounds 45-55

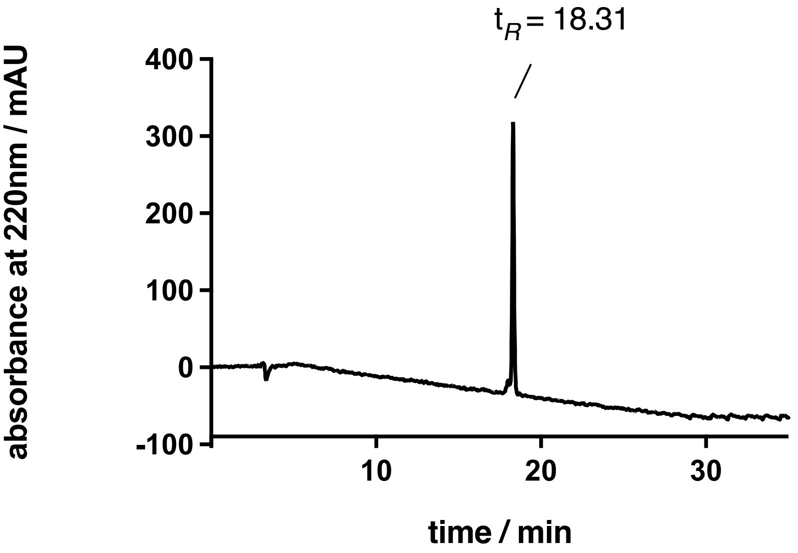

Figure S16. RP-HPLC analysis (purity control) of compound **45** (97%, 220 nm).

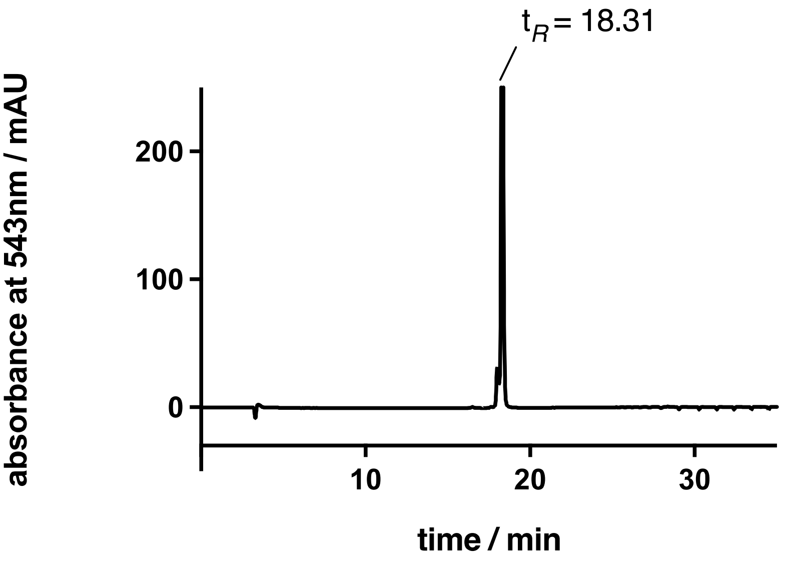

Figure S17. RP-HPLC analysis (purity control) of compound **45** (97%, 543 nm).

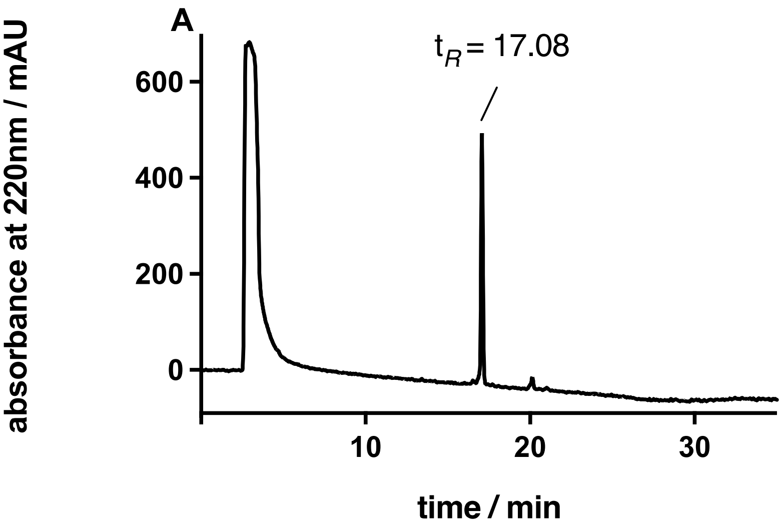

Figure S18. RP-HPLC analysis (purity control) of compound **46** (97%, 220 nm).

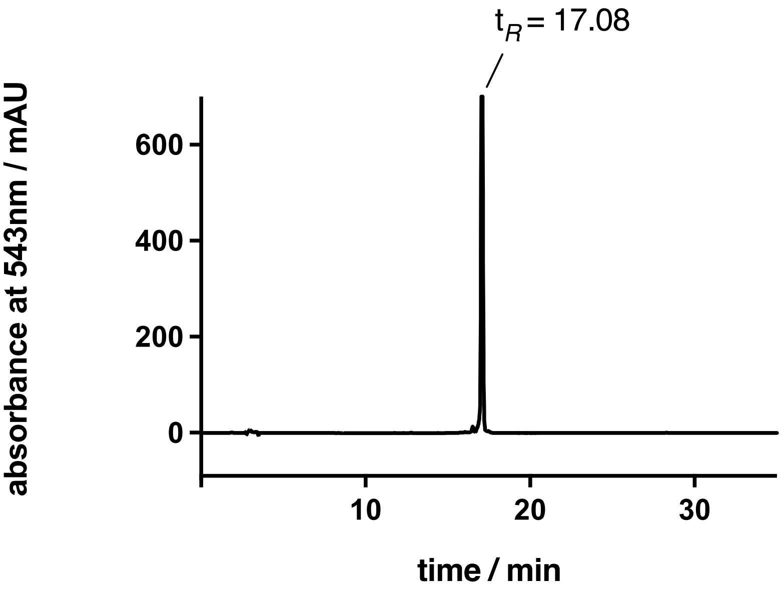

Figure S19. RP-HPLC analysis (purity control) of compound **46** (97%, 543 nm).

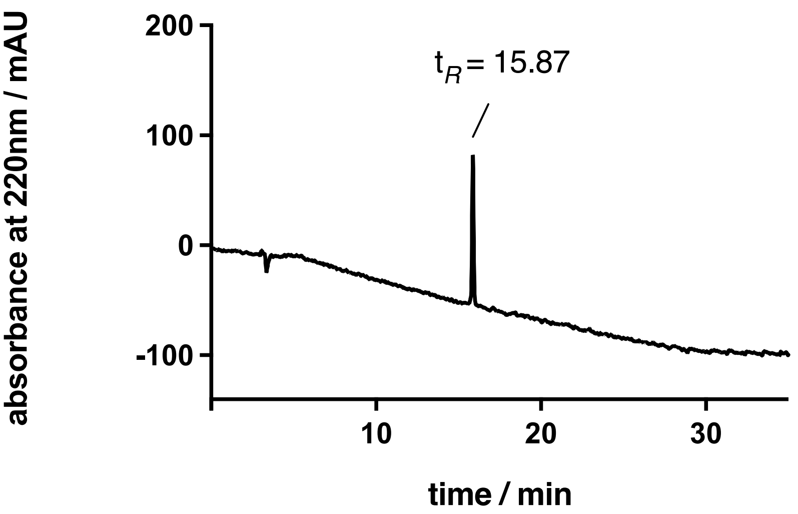

Figure S20. RP-HPLC analysis (purity control) of compound **47** (> 99%, 220 nm).

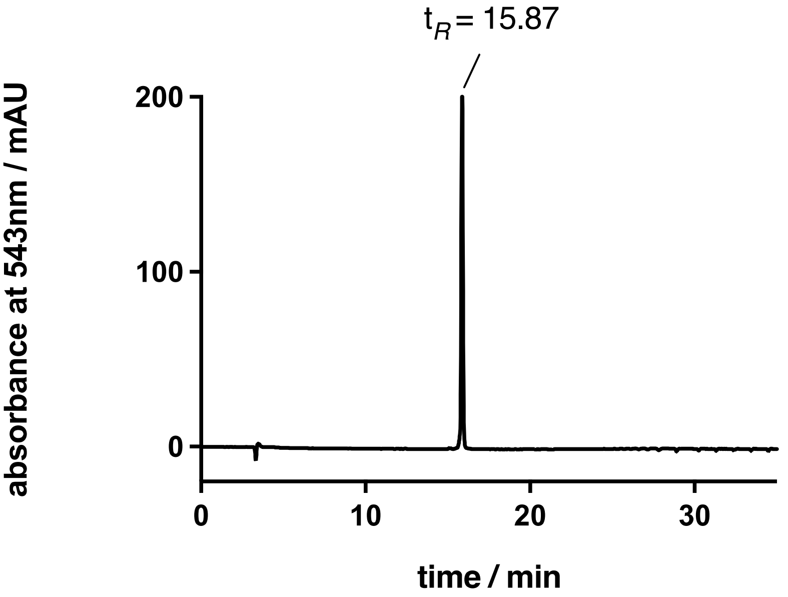

Figure S21. RP-HPLC analysis (purity control) of compound **47** (> 99%, 543 nm).

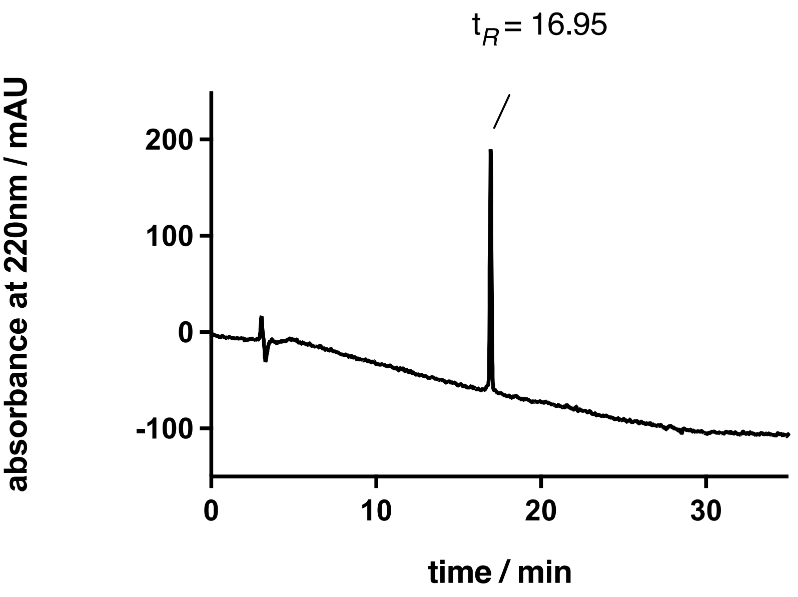

Figure S22. RP-HPLC analysis (purity control) of compound **48** (> 99%, 220 nm).

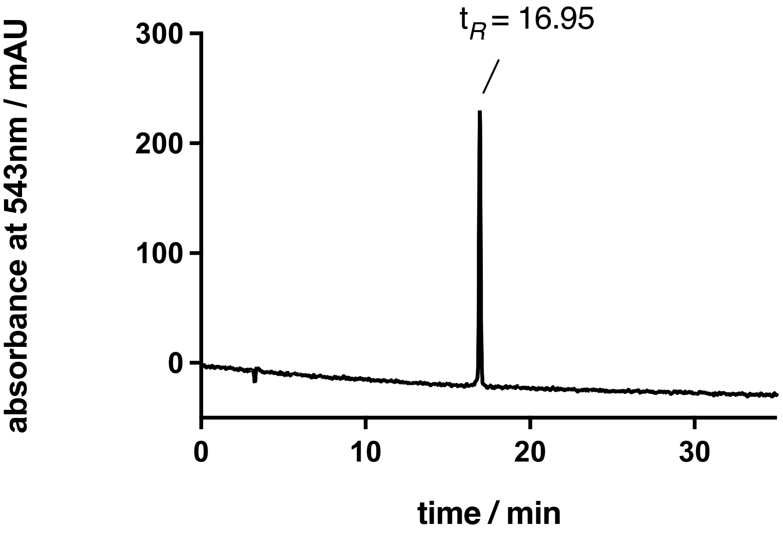

Figure S23. RP-HPLC analysis (purity control) of compound **48** (> 99%, 543 nm).

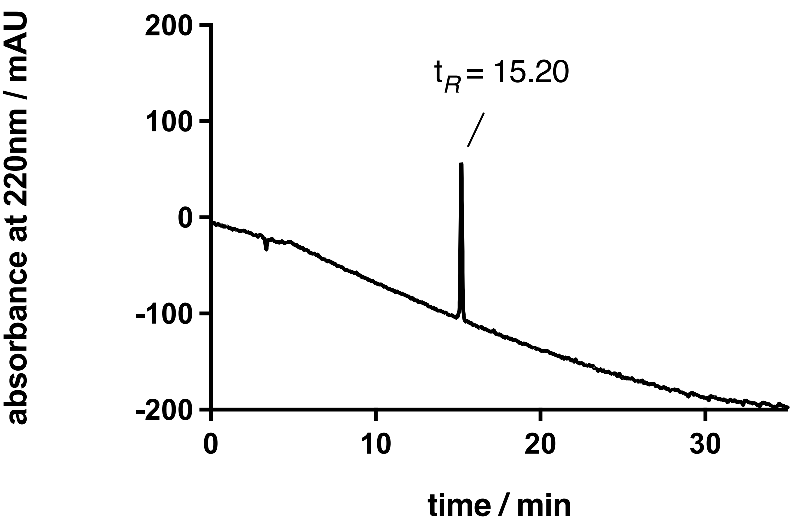

Figure S24. RP-HPLC analysis (purity control) of compound **49** (> 99%, 220 nm).

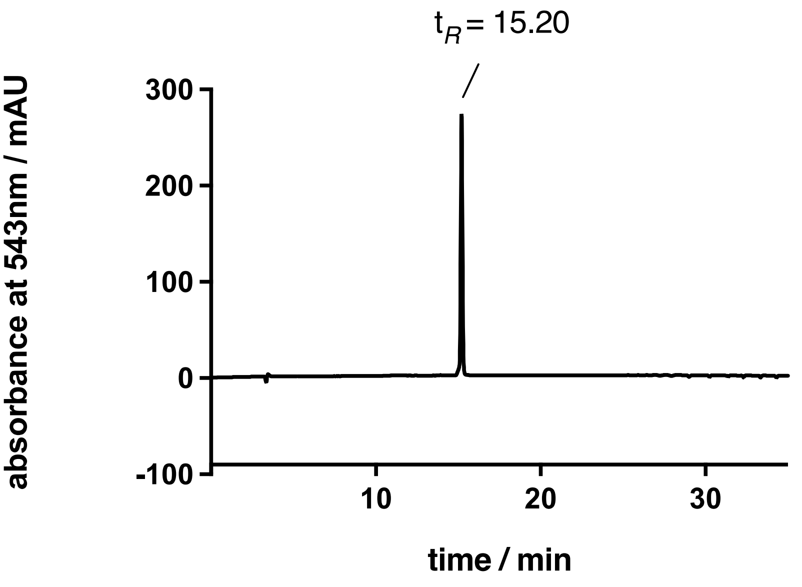

Figure S25. RP-HPLC analysis (purity control) of compound **49** (> 99%, 543 nm).

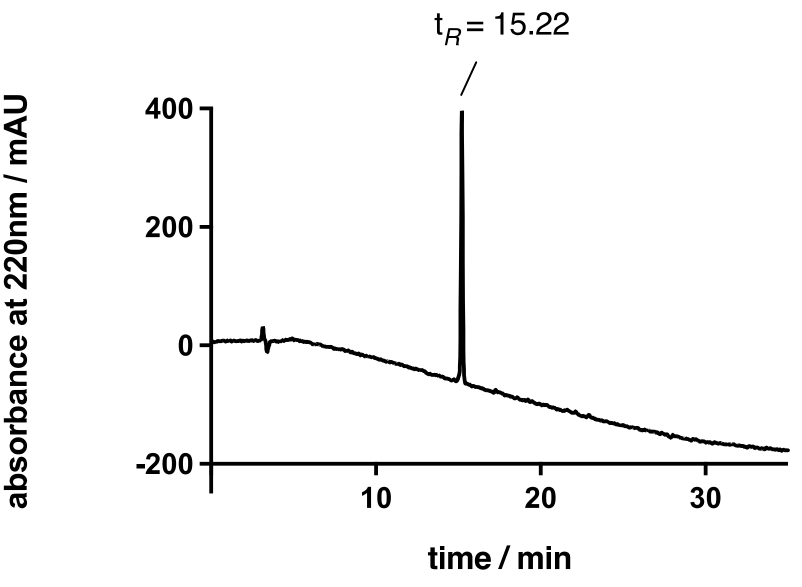

Figure S26. RP-HPLC analysis (purity control) of compound **50** (> 99%, 220 nm).

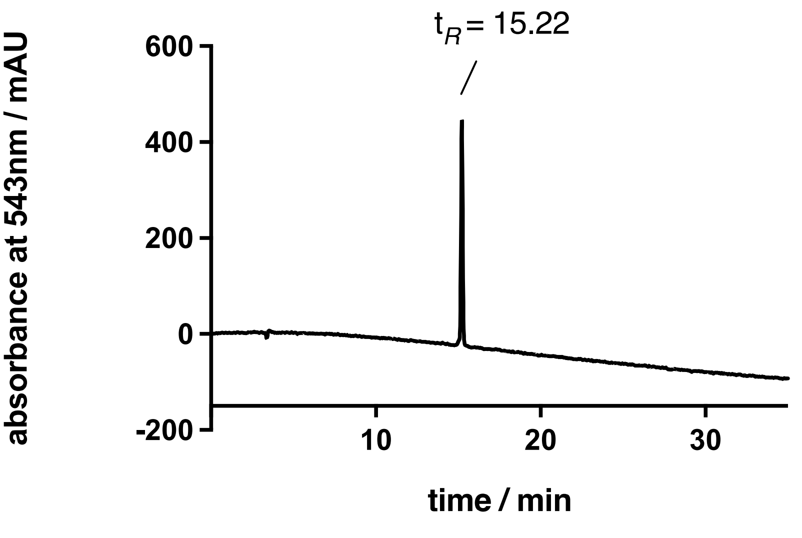

Figure S27. RP-HPLC analysis (purity control) of compound **50** (> 99%, 543 nm).

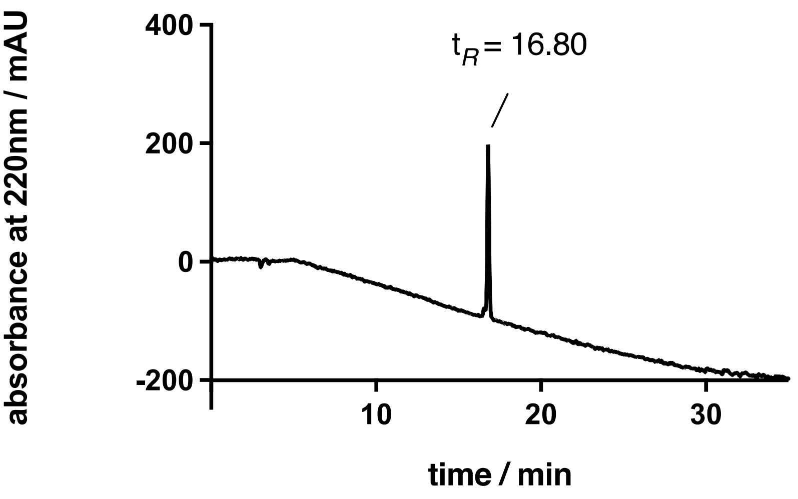

Figure S28. RP-HPLC analysis (purity control) of compound **51** (98%, 220 nm).

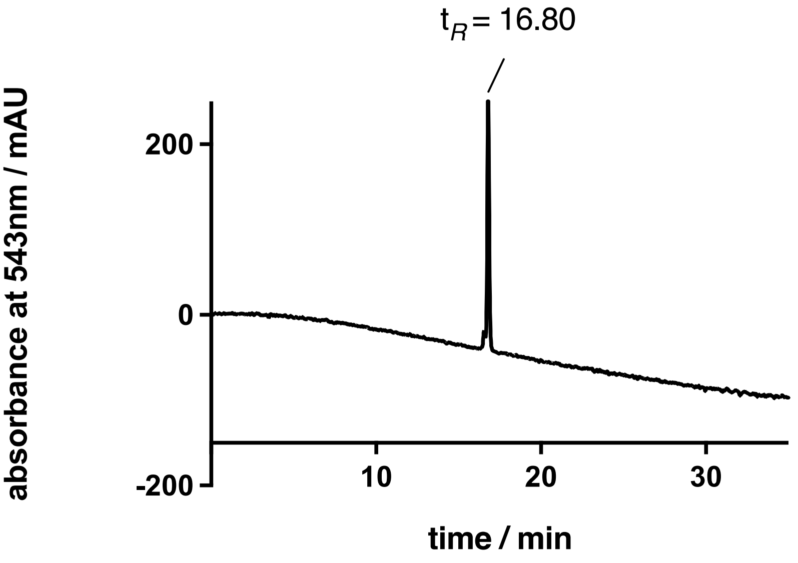

Figure S29. RP-HPLC analysis (purity control) of compound **51** (98%, 543 nm).

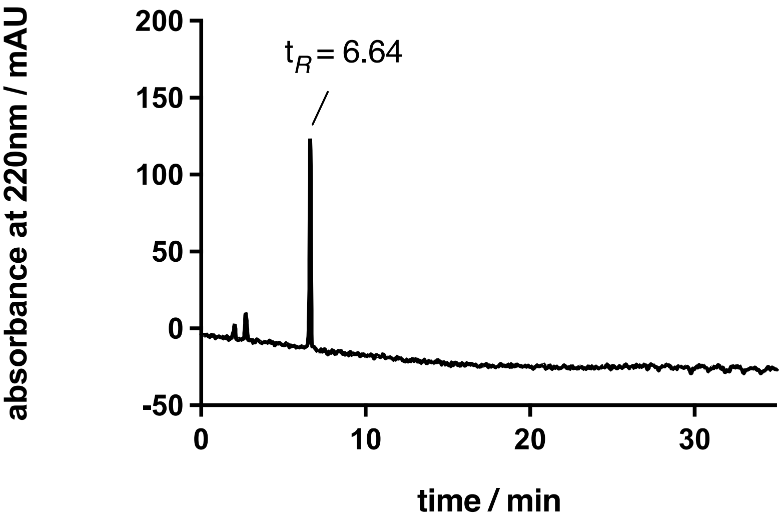

Figure S30. RP-HPLC analysis (purity control) of compound **52** (> 99%, 220 nm).

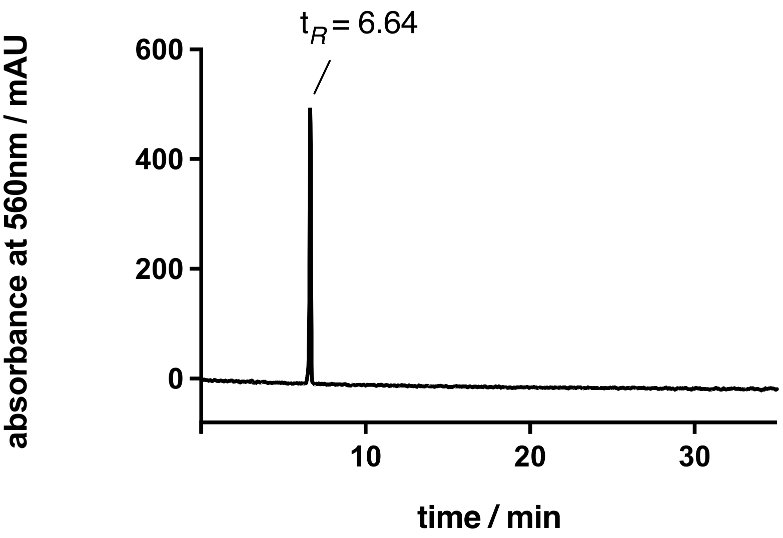

Figure S31. RP-HPLC analysis (purity control) of compound **52** (> 99%, 560 nm).

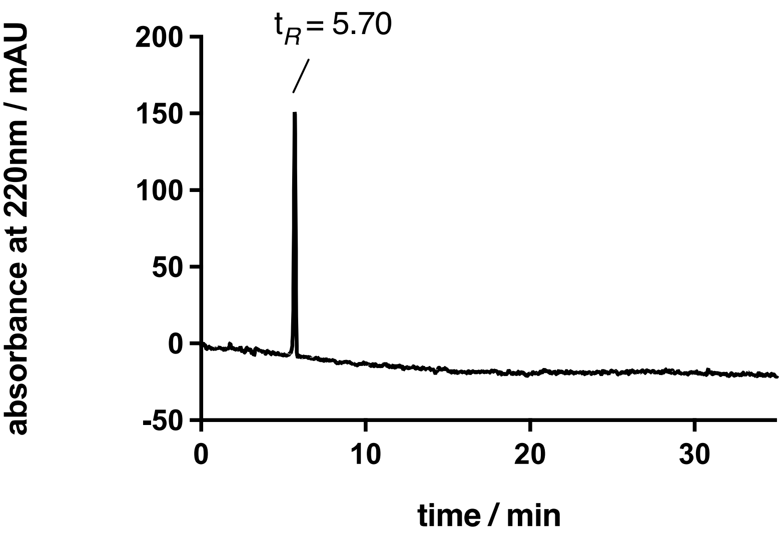

Figure S32. RP-HPLC analysis (purity control) of compound **53** (> 99%, 220 nm).

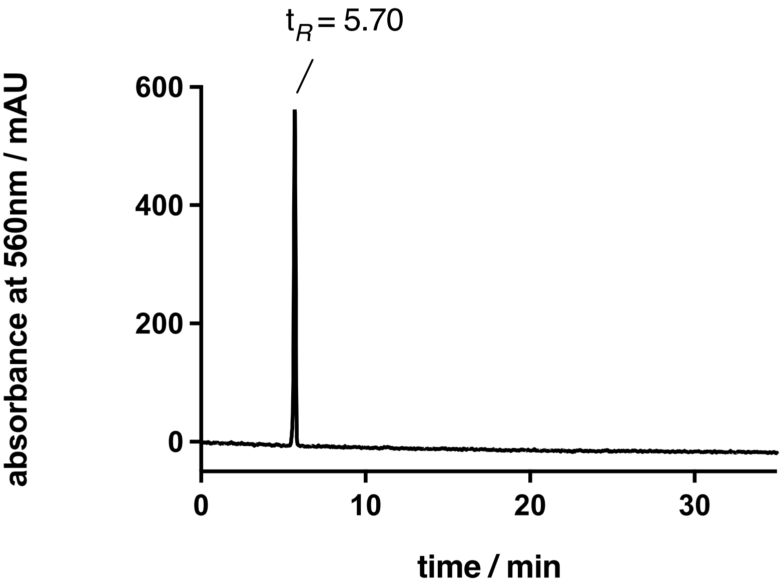

Figure S33. RP-HPLC analysis (purity control) of compound **53** (> 99%, 560 nm).

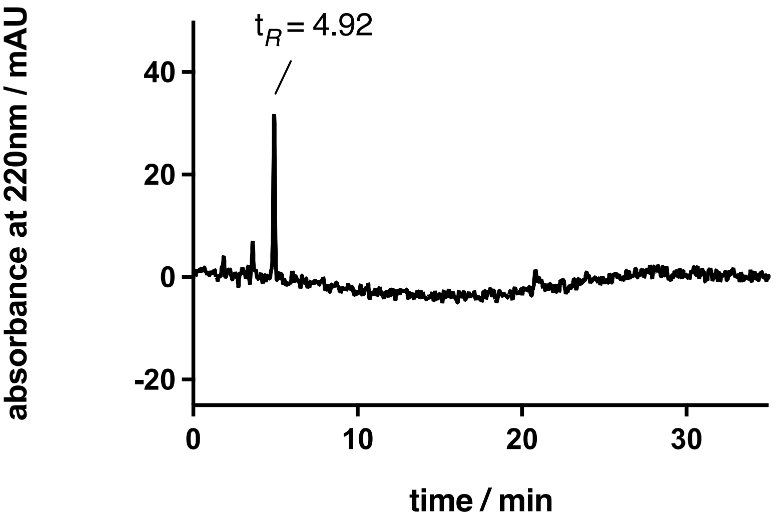

Figure S34. RP-HPLC analysis (purity control) of compound **54** (> 99%, 220 nm).

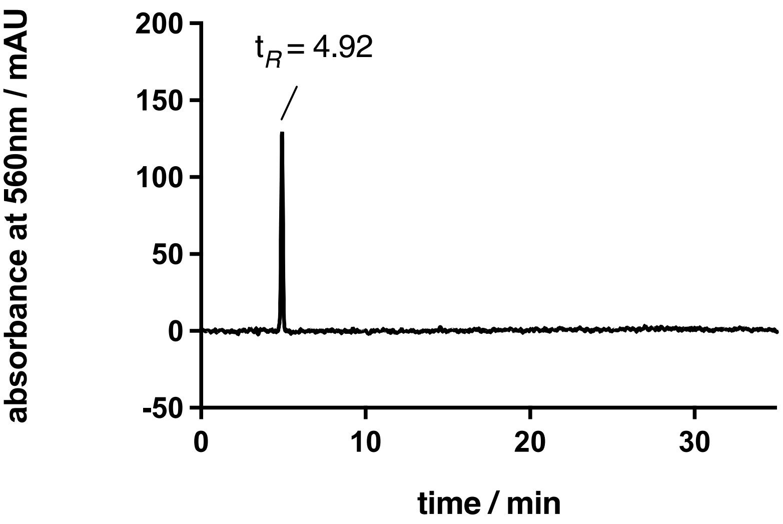

Figure S35. RP-HPLC analysis (purity control) of compound **54** (> 99%, 560 nm).

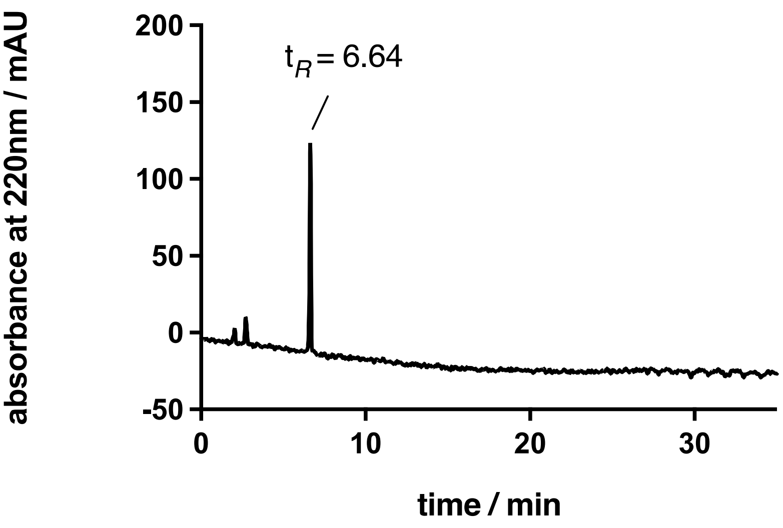

Figure S36. RP-HPLC analysis (purity control) of compound **55** (> 99%, 220 nm).

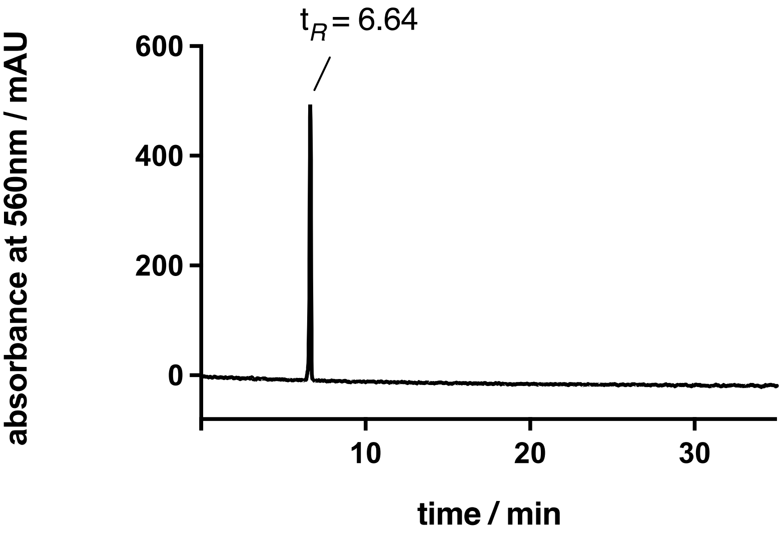

Figure S37. RP-HPLC analysis (purity control) of compound **55** (> 99%, 560 nm).

6. RP-HPLC stability control of compound 46 and 48

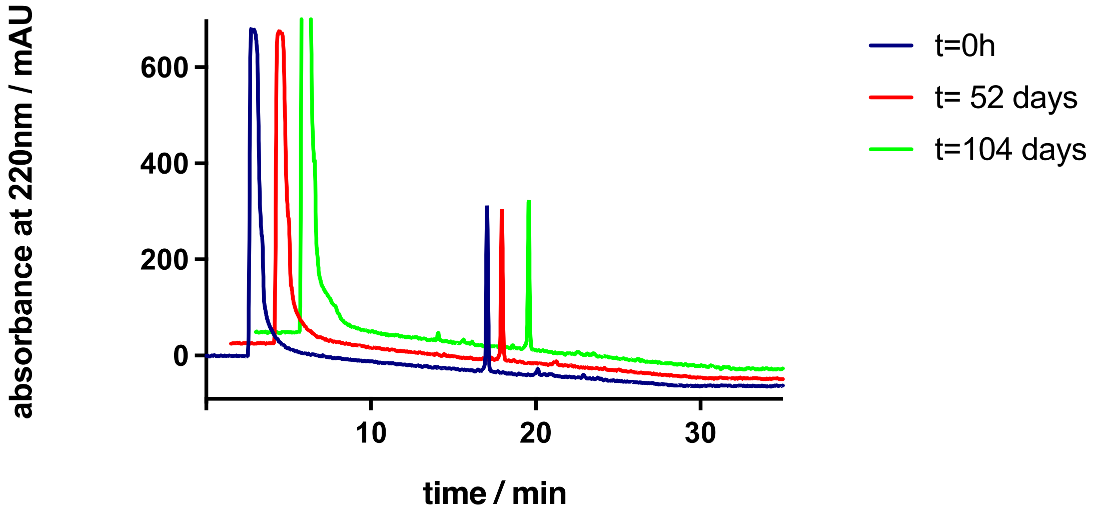

Figure S38. RP-HPLC analysis (stability control, 220 nm) of **46** at rt for 104 days in DMSO.

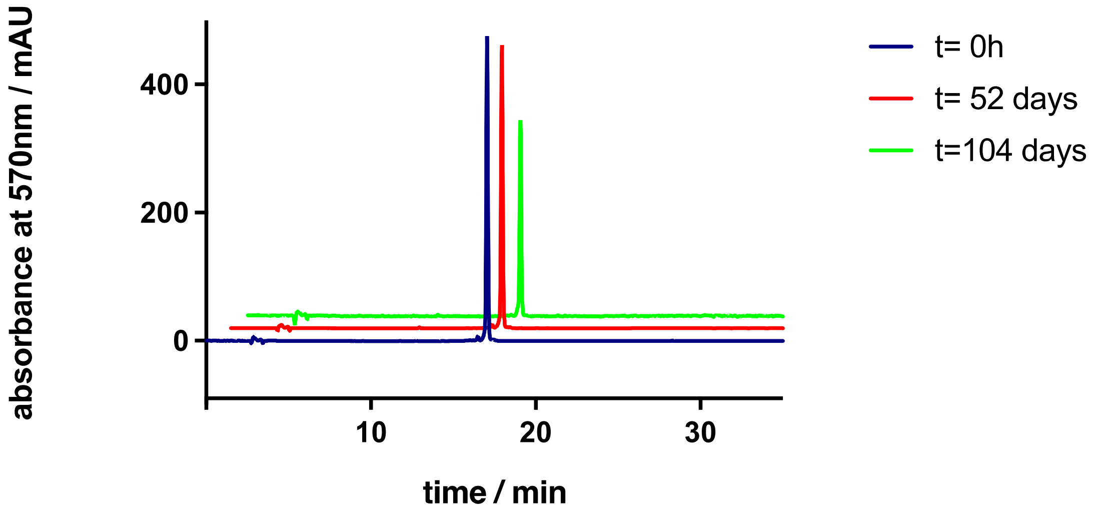

Figure S39. RP-HPLC analysis (stability control, 540 nm) of **46** at rt for 104 days in DMSO.

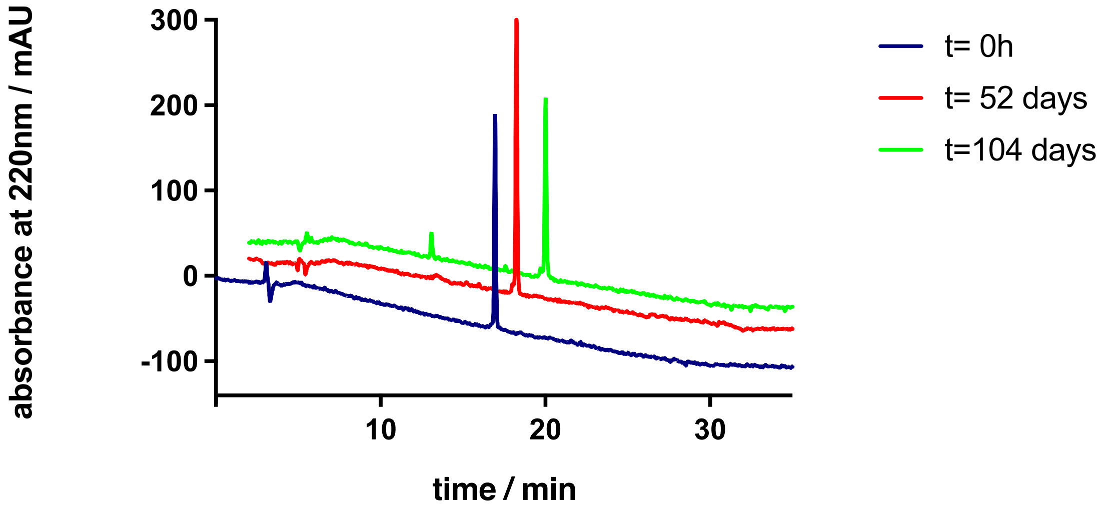

Figure S40. RP-HPLC analysis (stability control, 220 nm) of **48** at rt for 104 days in aqueous solution.

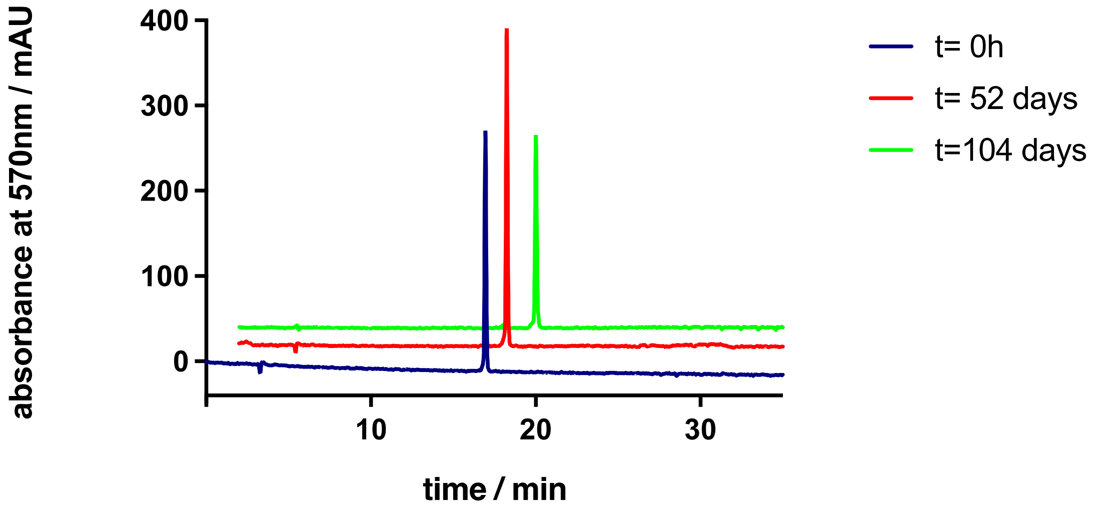

Figure S41. RP-HPLC analysis (stability control, 540 nm) of **48** at rt for 104 days in aqueous solution.

7. Fluorescence properties

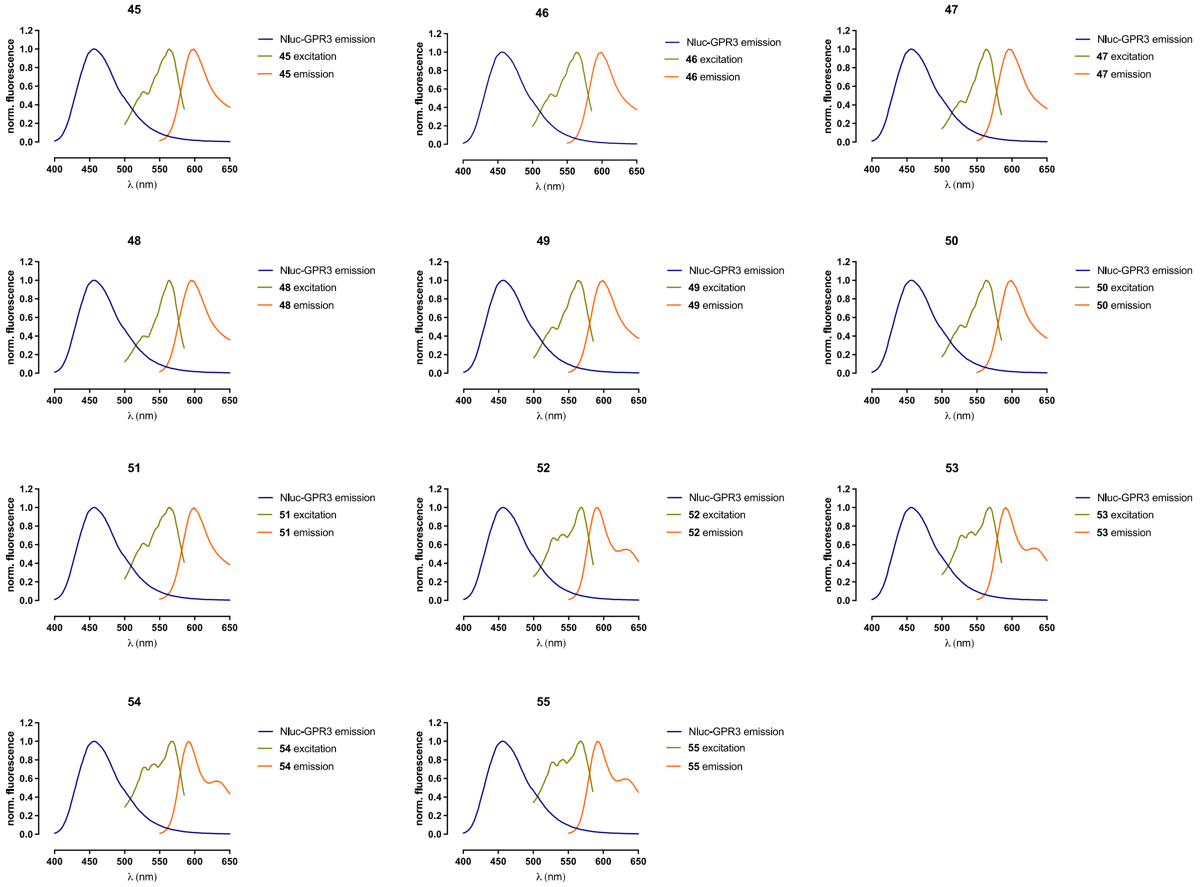

Figure S42. Corrected excitation and emission spectra of **45-55** overlayed with the bioluminescence emission spectrum of NLuc-GPR3 ($\lambda_{em,max}$ = 456 nm). Spectra of **45-55** were recorded in DMSO.

Table S1. Excitation/emission maxima of **45-55** determined in DMSO.

cmpd	$\lambda_{exc,max}/\lambda_{em,max}$ (nm)	cmpd	$\lambda_{exc,max}/\lambda_{em,max}$ (nm)
**45**	564 / 599	**51**	564 / 599
**46**	564 / 599	**52**	568 / 591
**47**	563 / 596	**53**	569 / 591
**48**	564 / 596	**54**	568 / 591
**49**	564 / 599	**55**	568 / 592
**50**	564 / 599		

8. Activity of 45 in a CRE reporter gene assay with GPR3, GPR6 and GPR12

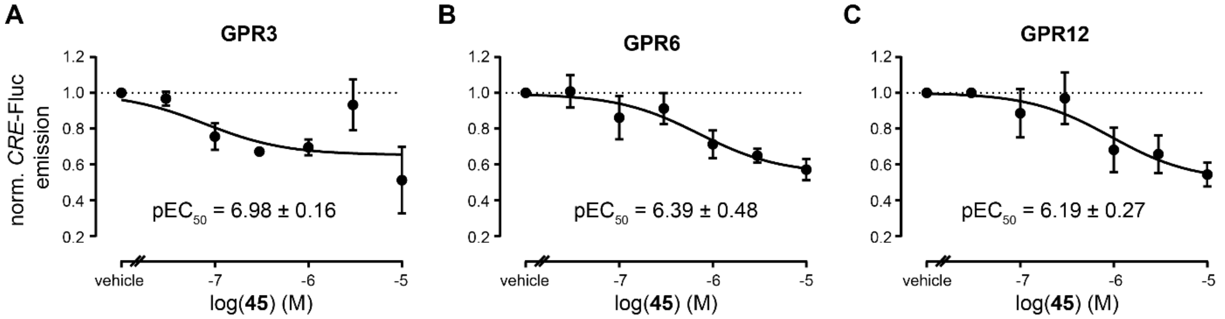

Figure S43. Normalized (to vehicle) *CRE* reporter gene response induced by **45** in cells co-transfected with GPR3 (**A**), GPR6 (**B**) or GPR12 (**C**). Data show mean ± SEM of three (A), four (B) or six (C) independent experiments conducted in transiently transfected HEK293 cells. No statistical difference was evident for the pEC_{50} values of compound **45** according to Extra-sum-of-squares F-test ($p < 0.05$).

9. Comparison of dissociation rates of *ortho*-labeled fluorescent AF64394 analogs

A

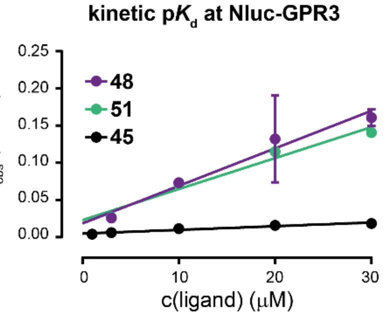

B

compound	linker type	k_{off} (min^{-1})
**45**	alkyl	0.005 ± 0.001
**48**	PEG4	0.019 ± 0.013
**51**	PEG6	0.023 ± 0.011

Figure S44. Effect of linker type on dissociation rates of *ortho*-labeled fluorescent AF64394 analogs. **A)** k_{obs} vs. ligand concentration graphs used for the determination of dissociation constants for compounds **45**, **48** and **51**. Data show mean ± SEM of three independent experiments. **B)** Dissocation rates (k_{off}) of **45**, **48** and **51**, which only differ in the linker type between the AF64394 scaffold and 5-TAMRA.

10. Physicochemical properties of 45 and UR-MN212

Table S2. Comparison of physicochemical properties of compound **45** and UR-MN212.

compound	molecular weight (g/mol)	TPSA (A^2)	csp^3	iLogP	number of H-bond donors	number of H-bond acceptors
**45**	919	137	0.28	4.1	1	8
**UR-MN212**	1012	162	0.30	2.1	2	9

Physicochemical properties were calculated using the SwissAdme webserver (http://www.swissadme.ch/)[14].

11. Basal BRET values recorded by different instruments

Table S3. Comparison of basal BRET values across instruments.

Figure	dataset	Plate reader*	mean raw basal BRET
3B	all concentrations	Tecan Spark® - machine A	0.024
3C	all concentrations	Tecan Spark® - machine A	0.024
3D	all concentrations	Tecan Spark® - machine A	0.024
3E	all concentrations	Tecan Spark® - machine A	0.024
3F	all concentrations	Tecan Spark® - machine A	0.024
3G	all concentrations	Tecan Spark® - machine A	0.024
3H	all concentrations	Tecan Spark® - machine A	0.017
3I	all concentrations	Tecan Spark® - machine A	0.024
3J	all concentrations	Tecan Spark® - machine A	0.017
3K	all concentrations	Tecan Spark® - machine A	0.017
3L	all concentrations	Tecan Spark® - machine A	0.017
4A	**45**	Tecan Spark® - machine A	0.017
4A	**46**	Tecan Spark® - machine A	0.024
4A	**47**	Tecan Spark® - machine A	0.024
4A	**48**	Tecan Spark® - machine A	0.024
4A	**51**	Tecan Spark® - machine A	0.017
4C	Nluc-β_1AR	Tecan Spark® - machine B	0.014
4C	Nluc-β_2AR	Tecan Spark® - machine B	0.015
4C	Nluc-AT_1R	Tecan Spark® - machine B	0.015
4C	Nluc-M_1R	Tecan Spark® - machine B	0.014
4C	Nluc-GPR3	Tecan Spark® - machine B	0.015
5A	Nluc-GPR3	Tecan Spark® - machine B	0.015
5A	Nluc-GPR6	Tecan Spark® - machine B	0.015
5A	Nluc-GPR12	Tecan Spark® - machine B	0.015
5B	1 μM **45** / 30 nM AF64394	Tecan Spark® - machine B	0.028
5C	1 μM **45** / 10 nM CVN424	Tecan Spark® - machine B	0.021
5D	1 μM **45** / Nluc-GPR3	Tecan Spark® - machine B	0.028
5D	1 μM **45** / Nluc-β_1AR	Tecan Spark® - machine B	0.028
5E	vehicle / vehicle	Tecan Spark® - machine B	0.015
5E	vehicle / 10 μM DPI	Tecan Spark® - machine B	0.015
6D	10 nM **45** / wt	BMG Labtech CLARIOstar Plus	0.029
6D	10 nM **45** / Y188W	BMG Labtech CLARIOstar Plus	0.026
6D	10 nM **45** / V187W/Y188W	BMG Labtech CLARIOstar Plus	0.025
6D	10 nM **45** / V186W/V187W/Y188W	BMG Labtech CLARIOstar Plus	0.027
6D	10 nM **45** / T279$^{7.35}$A/Y280$^{7.36}$A	BMG Labtech CLARIOstar Plus	0.027
6E	**45**	BMG Labtech CLARIOstar Plus	0.029
6E	UR-MN212	BMG Labtech CLARIOstar Plus	0.026

*: “machine A” and “machine B” refers to two different Tecan Spark instruments installed at distinct institutes.

12. Computational chemistry

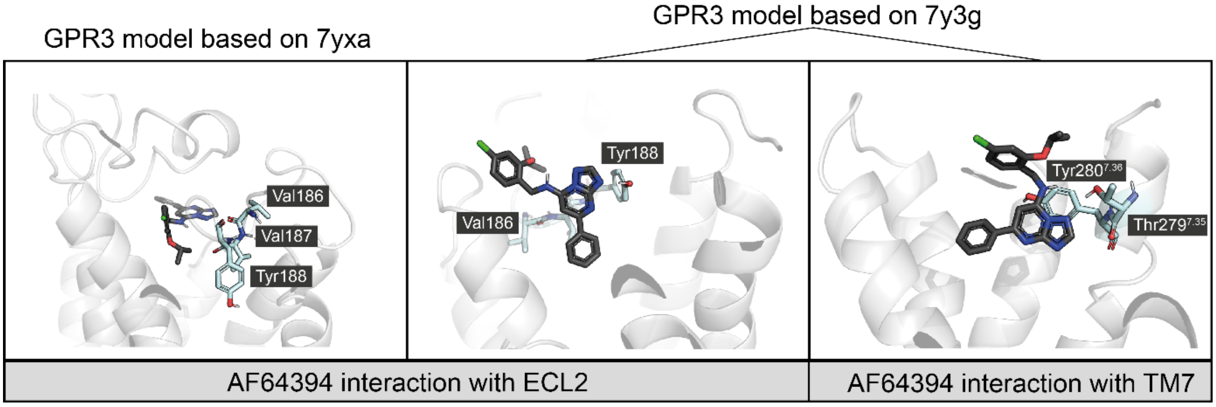

Figure S45. Depiction of two distinct putative binding modes of AF64394 in GPR3 homology models based on PDB IDs 7YXA (left) or 7Y3G (middle and right).

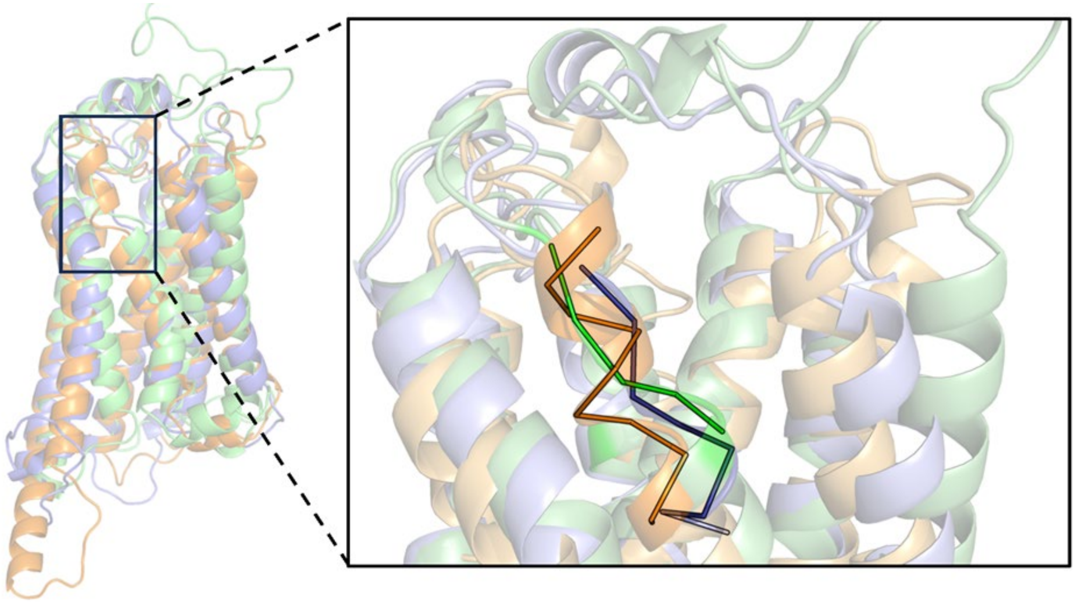

Figure S46. Superimposition of the three receptor models for GPR3. Color guide: purple: GPR3 homology model based on PDB ID 7EW1; green: GPR3 homology model based on PDB ID 7YXA; orange: GPR3 homology model based on PDB ID 7Y3G.

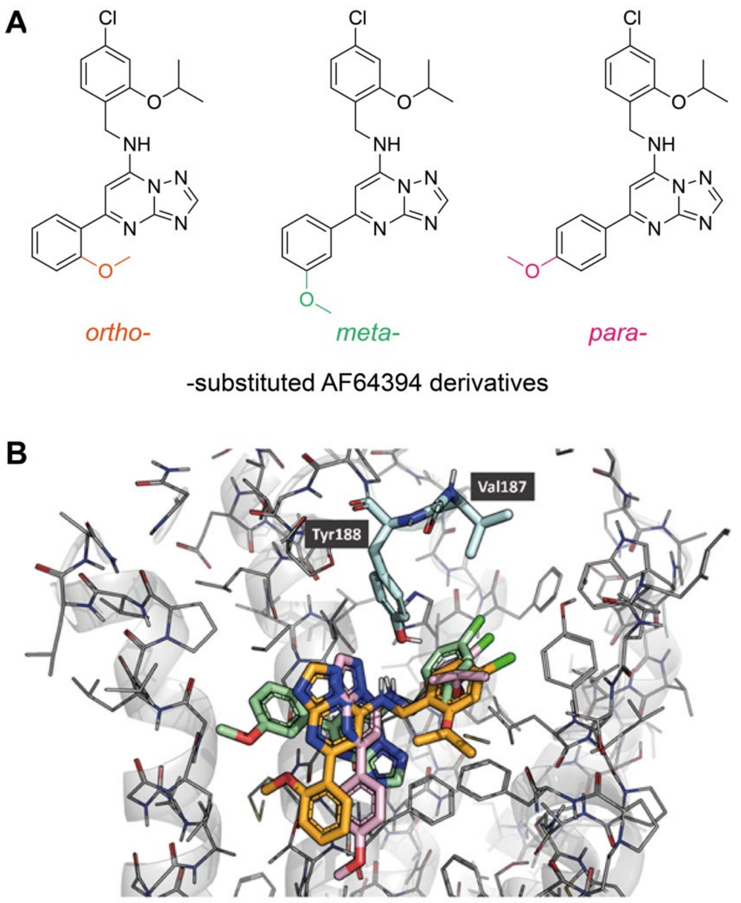

Figure S47. Docking poses of AF64394 derivatives to the active-state model of GPR3. **A)** Chemical 2D structures of docked AF64394 derivatives with methoxy substituents in *ortho*-, *meta*- or *para*-position of the phenyl ring. **B)** Side view of the docking poses of the three molecules when docked to the GPR3 model developed based on PDB ID 7EW1. Orange: ortho-, green: meta- and pink: para-substituted AF64394 derivative.

Table S4. Model statistics. Model representative colored in gray were used in the docking calculations.

Template PDB ID	#Cluster	Representative model	#Models	DOPE	MODELLER objective function	Normalized z-score	RMSD
7EW1							
	0	B99990353.pdb	221	-30928.842	2148.2905	0.987	1.15
	1	B99990068.pdb	41	-30854.664	1603.4868	1.006	1.17
	2	B99990011.pdb	34	-31040.699	1660.6511	0.958	1.21
	3	B99990373.pdb	31	-30775.529	1785.2535	1.026	1.13
	4	B99990157.pdb	22	-30620.879	1741.6594	1.066	1.2
7YXA							
	0	B99990364.pdb	155	-35546.75	1798.2273	0.287	1.95
	1	B99990146.pdb	65	-35827.797	1795.4095	0.219	1.39
	2	B99990473.pdb	47	-35724.945	1794.1129	0.244	1.88
	3	B99990165.pdb	25	-35637.68	1760.5212	0.265	1.79
	4	B99990427.pdb	25	-35887.918	1844.0547	0.205	1.97
7Y3G							
	0	B99990284.pdb	53	-31762.92	1209.4812	0.677	0.307
	1	B99990170.pdb	37	-31987.080	1204.8276	0.617	0.380
	2	B99990055.pdb	35	-31866.787	1274.3088	0.649	0.337
	3	B99990251.pdb	34	-31715.85	1273.5842	0.689	0.5
	4	B99990405.pdb	31	-21551.460	1200.1917	0.733	1.12

13. References

(1) Mousli, Y.; Rouvière, L.; Traboulsi, I.; Hunel, J.; Buffeteau, T.; Heuzé, K.; Vellutini, L.; Genin, E. Hydrosilylation of Azide-Containing Olefins as a Convenient Access to Azidoorganotrialkoxysilanes for Self-Assembled Monolayer Elaboration onto Silica by Spin Coating. *ChemistrySelect* **2018**, *3* (25), 7333–7339.

(2) Kim, J.; Morozumi, T.; Kurumatani, N.; Nakamura, H. Novel Chemosensor for Alkaline Earth Metal Ion Based on 9-Anthryl Aromatic Amide Using a Naphthalene as a TICT Control Site and Intramolecular Energy Transfer Donor. *Tetrahedron Letters* **2008**, *49* (12), 1984–1987.

(3) Kalyanaraman, B.; Chitambar, C. R. Synergistic Inhibition of Tumor Cell Proliferation Induced by Combined Treatment of Metformin Compounds and Iron Chelators. WO2018/119207A1, 2018.

(4) Kim, J. K.; Park, S.; Yoo, R. J.; Jeong, H. J.; Oh, J.; Lee, Y. J.; Park, S.; Kim, D. W. Thin PEGylated Carbon Nitrides: Water-Dispersible Organic Nanodots as Bioimaging Probes. *Chemistry–A European Journal* **2018**, *24* (14), 3506–3511.

(5) Ji, A.; Marvin, M.; Marks, K.; Anderson, D. Oligonucleotide Synthesis on Solid Support. WO2021/173615A1, 2021.

(6) Nagasawa, S.; Fujiki, S.; Sasano, Y.; Iwabuchi, Y. Chromium–Salen Complex/Nitroxyl Radical Cooperative Catalysis: A Combination for Aerobic Intramolecular Dearomative Coupling of Phenols. *The Journal of Organic Chemistry* **2021**, *86* (9), 6952–6968.

(7) El Bakali, J.; Klupsch, F.; Guédin, A.; Brassart, B.; Fontaine, G.; Farce, A.; Roussel, P.; Houssin, R.; Bernier, J.-L.; Chavatte, P. 2, 6-Diphenylthiazolo [3, 2-b][1, 2, 4] Triazoles as Telomeric G-Quadruplex Stabilizers. *Bioorganic & medicinal chemistry letters* **2009**, *19* (13), 3434–3438.

(8) Ahn, J. H.; Shin, M. S.; Jung, S. H.; Kim, J. A.; Kim, H. M.; Kim, S. H.; Kang, S. K.; Kim, K. R.; Dal Rhee, S.; Park, S. D.; Lee, J. M.; Lee, J. H.; Cheon, H. G.; Kim, S. S. Synthesis and Structure–Activity Relationship of Novel Indene N-Oxide Derivatives as Potent Peroxisome Proliferator Activated Receptor γ (PPARγ) Agonists. *Bioorganic & medicinal chemistry letters* **2007**, *17* (18), 5239–5244.

(9) Jiang, Y.; Chen, X.; Zheng, Y.; Xue, Z.; Shu, C.; Yuan, W.; Zhang, X. Highly Diastereoselective and Enantioselective Synthesis of A-Hydroxy B-Amino Acid Derivatives: Lewis Base Catalyzed Hydrosilylation of A-Acetoxy B-Enamino Esters. *Angewandte Chemie* **2011**, *123* (32), 7442–7445.

(10) Ding, C.; Liu, Y.; Wang, T.; Fu, J. Triple-Stimuli-Responsive Nanocontainers Assembled by Water-Soluble Pillar [5] Arene-Based Pseudorotaxanes for Controlled Release. *Journal of Materials Chemistry B* **2016**, *4* (16), 2819–2827.

(11) Jensen, T.; Elster, L.; Nielsen, S. M.; Poda, S. B.; Loechel, F.; Volbracht, C.; Klewe, I. V.; David, L.; Watson, S. P. The Identification of GPR3 Inverse Agonist AF64394; the First Small Molecule Inhibitor of GPR3 Receptor Function. *Bioorg Med Chem Lett* **2014**, *24* (22), 5195–5198.

(12) Wu, F.; Bai, R.; Gu, Y. Synthesis of Benzofurans from Ketones and 1, 4-Benzoquinones. *Advanced Synthesis & Catalysis* **2016**, *358* (14), 2307–2316.

(13) Charlton, J. L.; Lypka, G.; Sayeed, V. The Synthesis of 2-methylchromone-3-carboxylic Acid. *Journal of Heterocyclic Chemistry* **1980**, *17* (3), 593–594.

(14) Daina, A.; Michielin, O.; Zoete, V. SwissADME: a free web tool to evaluate pharmacokinetics, drug-likeness and medicinal chemistry friendliness of small molecules. *Sci Rep* **2017**, *7* (42717)
